# Evidence of Blood Stage Efficacy with a Virosomal Malaria Vaccine in a Phase IIa Clinical Trial

**DOI:** 10.1371/journal.pone.0001493

**Published:** 2008-01-30

**Authors:** Fiona M. Thompson, David W. Porter, Shinji L. Okitsu, Nicole Westerfeld, Denise Vogel, Stephen Todryk, Ian Poulton, Simon Correa, Claire Hutchings, Tamara Berthoud, Susanna Dunachie, Laura Andrews, Jack L. Williams, Robert Sinden, Sarah C. Gilbert, Gerd Pluschke, Rinaldo Zurbriggen, Adrian V. S. Hill

**Affiliations:** 1 Centre for Clinical Vaccinology and Tropical Medicine, University of Oxford, Oxford, United Kingdom; 2 Swiss Tropical Institute, Basel, Switzerland; 3 Pevion Biotech Ltd., Bern, Switzerland; 4 Medical Research Council (MRC) Research Laboratories, Fajara, The Gambia; 5 Division of Communicable Diseases and Immunology, Walter Reed Army Institute of Research, Silver Spring, Maryland, United States of America; 6 Infection and Immunity Section, Biology Department, Imperial College London, United Kingdom; London School of Hygiene & Tropical Medicine, United Kingdom

## Abstract

**Background:**

Previous research indicates that a combination vaccine targeting different stages of the malaria life cycle is likely to provide the most effective malaria vaccine. This trial was the first to combine two existing vaccination strategies to produce a vaccine that induces immune responses to both the pre-erythrocytic and blood stages of the *P. falciparum* life cycle.

**Methods:**

This was a Phase I/IIa study of a new combination malaria vaccine FFM ME-TRAP+PEV3A. PEV3A includes peptides from both the pre-erythrocytic circumsporozoite protein and the blood-stage antigen AMA-1. This study was conducted at the Centre for Clinical Vaccinology and Tropical Medicine, University of Oxford, Oxford, UK. The participants were healthy, malaria naïve volunteers, from Oxford. The interventions were vaccination with PEV3A alone, or PEV3A+FFM ME-TRAP. The main outcome measure was protection from malaria in a sporozoite challenge model. Other outcomes included measures of parasite specific immune responses induced by either vaccine; and safety, assessed by collection of adverse event data.

**Results:**

We observed evidence of blood stage immunity in PEV3A vaccinated volunteers, but no volunteers were completely protected from malaria. PEV3A induced high antibody titres, and antibodies bound parasites in immunofluorescence assays. Moreover, we observed boosting of the vaccine-induced immune response by sporozoite challenge. Immune responses induced by FFM ME-TRAP were unexpectedly low. The vaccines were safe, with comparable side effect profiles to previous trials. Although there was no sterile protection two major observations support an effect of the vaccine-induced response on blood stage parasites: (i) Lower rates of parasite growth were observed in volunteers vaccinated with PEV3A compared to unvaccinated controls (p = 0.012), and this was reflected in the PCR results from PEV3A vaccinated volunteers. These showed early control of parasitaemia by some volunteers in this group. One volunteer, who received PEV3A alone, was diagnosed very late, on day 20 compared to an average of 11.8 days in unvaccinated controls. (ii). Morphologically abnormal parasites were present in the blood of all (n = 24) PEV3A vaccinated volunteers, and in only 2/6 controls (p = 0.001). We describe evidence of vaccine-induced blood stage efficacy for the first time in a sporozoite challenge study.

**Trial Registration:**

ClinicalTrials.Gov NCT00408668

## Introduction

Malaria represents a huge burden of global disease, affecting approximately 40% of the world's population. It is estimated that there were 515 million clinical episodes of *P. falciparum* malaria in 2002 [1] and at least a million people die from the disease annually. An effective vaccine could have an enormous impact on this problem, both for people in the developing world and for those travelling to malaria endemic countries [1], [2]. It has long been recognised that a multi-stage vaccine is likely to provide the greatest level of protection against *P. falciparum* malaria [3], [4].

This trial is the first to evaluate clinically the combined administration of two promising malaria vaccines targeting different life-cycle stages: FP9/MVA ME-TRAP and PEV3A. Recent studies in murine malaria, assessing the combination of an anti-sporozoite antibody-inducing vaccine with an anti-liver-stage T cell-inducing vaccine [5], suggested that combining these vaccines could be synergistic leading to enhanced protection.

Fowlpox strain FP9 and modified vaccinia virus Ankara (MVA) vectors expressing the pre-erythrocytic antigen thrombospondin-related adhesion protein (TRAP), fused to a multi-epitope (ME) string were developed by the University of Oxford [6]. When used in a heterologous prime-boost regimen in Oxford these vaccines induced strong T cell responses, and significantly reduced parasite numbers emerging from the liver by about 90% [7], with some individuals completely protected from malaria challenge. This protection persisted in one individual on two further challenges at 14 and 20 months after vaccination [8]. These encouraging data led to the assessment of FP9-MVA ME-TRAP in a series of phase I/II studies in adults and in children in Gambia [9] and in Kenya, where lower T cell immunogenicity has been observed [10].

PEV3A was developed by Pevion Biotech in collaboration with the Swiss Tropical Institute [11], [12]. This vaccine uses an influenza virosome-based technology, which has been approved for human use in more than 40 countries. Initially developed as an influenza vaccine itself, it can also be used as an antigen delivery system (e.g. hepatitis A vaccine) [13]. PEV3A is a virosomal formulation of two malaria antigens. These are peptides derived from the circumsporozoite (CS) protein and apical membrane antigen-1 (AMA-1) of the K1 isolate of *P. falciparum*. The peptide from the CS protein is an internally cyclised double loop of 5 NPNA repeats, the major B cell epitope of the CS protein [12]. Antibodies against this peptide inhibit sporozoite motility and invasion capability. The peptide from AMA-1 mimics the semi-conserved loop I of domain III and has been found capable of inducing antibodies that, as monoclonal antibodies, impair the growth of blood stage *P. falciparum* parasites [11]. Both peptides are linked to phosphatidylethanolamine (PE), which intercalates into the virosomal membrane, thereby displaying the attached peptides on the surface of the virosomes. Each peptide was incorporated into virosomes separately. Both vaccine components applied alone and in combination have been previously used in a Phase I study in Switzerland and found to be safe and immunogenic [12], [14]. We report a Phase I/IIa sporozoite challenge study that indicates that this bivalent peptide vaccine induces immune responses that have an inhibitory effect on blood stage parasites.

## Methods

### Participants

The protocol for this trial and supporting CONSORT checklist are available as supporting information; see Checklist S1 and Protocol S1 and Addendum S1. Healthy malaria naïve adult subjects aged 18–50 years were recruited in the Oxford area from August 2005 and underwent medical screening as previously described [15]. Exclusion criteria included a prior history of malaria, immunosuppression, epilepsy, infection with hepatitis B, hepatitis C or HIV, pregnancy, drug or alcohol abuse, significant psychiatric disorder or other significant illness. All vaccinations and follow up visits took place in the outpatients unit at the Centre for Clinical Vaccinology and Tropical Medicine, part of the University of Oxford at the Churchill Hospital. The malaria challenge was performed in the insectary in the Alexander Fleming Building, Imperial College London.

### Ethics

**Figure 1:**
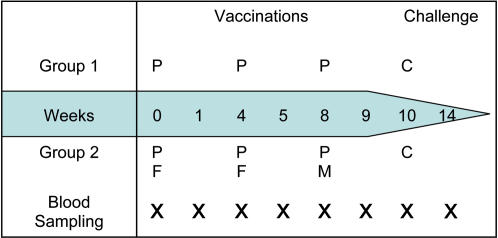
Study Design. P = Vaccination with PEV3A, F = Vaccination with FP9 ME-TRAP, M = Vaccination with MVA ME-TRAP, C = Sporozoite challenge

The study received ethical approval from the Oxfordshire Research Ethics Committee A, the approval is available as supporting information Ethics Approval Letter S1; and was conducted under a Clinical Trial Authorisation from the MHRA. An Independent Local Safety Monitor was appointed in Oxford. All volunteers provided fully informed consent to participate in this study by signing a written consent form, prior to any study procedures. A copy of the consent form is available as supporting document Consent Form S1. The trial was conducted according to GCP and the principles of The Declaration of Helsinki, and was externally monitored by Appledown Clinical Research Ltd. The study design is represented pictorially in Figure 1.

### Interventions

PEV3A is a virosomal vaccine preparation manufactured by Pevion Biotech Ltd., Switzerland. The vaccine carries two synthetic *P. falciparum* peptide-PE conjugates derived from the circumsporozoite protein (UK-39) and the apical membrane antigen-1 (AMA49-C1). The sequences of both of these peptides were derived from the K1 isolate of *P. falciparum*. PEV3A was imported from Switzerland in vials of 0.5 mL containing 50 µg of virosomally-formulated AMA49-C1 (PEV301) and 10 µg of virosomally-formulated UK-39 (PEV302) in phosphate buffered saline (pH 7.4), and stored at +2 to +8°C. This dose was selected following a preliminary phase I study [14] and was shown to be immunogenic. ME-TRAP is a multiple epitope string including fourteen CD8+ T cell epitopes, one CD4+ T cell epitope, and two B cell epitopes from six pre-erythrocytic *P. falciparum* antigens fused to the N-terminus of TRAP as previously described [6]. The ME string includes copies of the B cell epitope NANP sequence derived from the circumsporozoite protein, also used in UK39 in PEV3A, as well as 3 CD8 and one CD4 T cell epitopes, but no AMA-1 derived epitopes. For more detail see [6]. FP9 and MVA ME-TRAP were manufactured by a contract manufacturer (IDT, Germany). MVA and FP9 ME-TRAP were stored at −20°C and allowed to thaw prior to administration. The potency of these vaccines was tested prior to the trial in a standardised assay on Balb/c mice, 14 days following administration of the vaccine; peptide specific ELISPOT responses were measured in splenic lymphocytes. 24 volunteers were enrolled into the study, with an additional 6 unvaccinated controls for the malaria challenge. Group 1 volunteers received three doses of PEV3A 0.5 mL (P) given intramuscularly at baseline, 4 weeks later and 8 weeks. Group 2 volunteers received a combination of PEV3A 0.5 mL given intramuscularly (P) and FP9 ME-TRAP 1×10^8^ plaque forming units (pfu) given intradermally (F), at baseline; (P) and (F) 4 weeks later; and (P) and MVA ME-TRAP 1.5×10^8^ pfu given intradermally (M) at 8 weeks. Intramuscular injections were given into the left deltoid; intradermal injections were administered into the skin over the right deltoid. Up to 80 ml of blood was drawn at day 0, 7, 28, 35, 56, 63, day of challenge (day 70), challenge+7 days (day 77), challenge+35 days (day 105) and challenge+90 days (day 160) for safety assessment and measurement of immunogenicity.

### Objectives

This trial had 3 objectives; firstly, to assess protection against *P. falciparum* sporozoite challenge following immunisation with the virosomal vaccine PEV3A alone or in combination with FP9-MVA ME-TRAP; secondly, to evaluate the immunogenicity of these regimes with measures of anti parasite immunity and, thirdly, to assess the safety of these vaccination regimes.

### Outcomes

#### 1. Vaccine efficacy

The primary outcome was protection against malaria infection in a *P. falciparum* sporozoite challenge model. To assess the efficacy of the vaccines the 24 vaccinated subjects and 6 unvaccinated infectivity control subjects underwent experimental challenge with *Plasmodium falciparum*, fourteen days after the final vaccination. Laboratory-reared *Anopheles stephensi* mosquitoes were infected with the chloroquine-sensitive 3D7 strain of *P. falciparum* parasites in an adapted model [16] as described before [6], to assess the efficacy of the vaccines. From the evening of day 6 subjects attended clinic twice daily for review of symptoms, vital signs monitoring (pulse, blood pressure and oral temperature) and withdrawal of 3 mL of blood for thick film and PCR analysis. Field's stain thick films were examined immediately by experienced microscopists for the appearance of viable parasites. A minimum of 200 high power fields were examined before a subject was declared slide negative. Subjects who reached day 15 without blood film evidence of malaria infection were followed up daily until day 21. All subjects were treated immediately with Riamet (artemether 20 mg, lumefantrine 120 mg, *Novartis*) on diagnosis of malaria by the identification of a viable parasite on thick film. Subjects returned to clinic on two consecutive days for negative blood films post treatment. During the challenge follow-up period blood samples were analysed by PCR in real time (method discussed in [17]), the clinicians assessing the subjects were blinded to the results. Efficacy was assessed by measuring the number of subjects who developed malaria infection and the time between exposure and parasitaemia as detected by thick-film blood smear, as well as measurement of parasite growth rates by PCR. Comparisons were made between the two vaccine groups and between all vaccinated volunteers and unvaccinated controls. Parasite growth rates were calculated using a method previously described [18]. This method is based on a statistical model of parasite distribution, using a convolution of two probability density functions to estimate the numbers of parasites present in the blood and being sequestered at any time. The model was coded into an Excel ™ spreadsheet, and the in-built Solver minimization routine was used to estimate the best solution by minimization of the squared difference between calculated and predicted values.

#### 2. Immunogenicity

Vaccine immunogenicity was assessed by IFN-γ ELISPOT and ELISA for vaccine specific antibodies. ELISPOT was performed on PBMCs obtained from each volunteer at the above-described time points as reported previously [19]. Anti-UK-39 and anti-AMA49-C1 antibodies were measured by ELISA. ELISA polysorp microtitre plates (Nunc, Dr. Grogg, Stetten-Deiswill, Switzerland) were coated at 4°C overnight with 10 µg/ml AMA49-C1 (for PEV301) or UK-39 (for PEV302) in PBS, pH 7.4. Wells were then blocked with 5% milk powder in PBS for 2 h at 37°C followed by three washes with PBS containing 0.05% Tween-20. Plates were then incubated with two-fold serial dilutions of human serum starting with 1∶50 in PBS containing 0.05% Tween-20 and 0.5% milk powder for 2 h at 37°C. After washing, the plates were incubated with horseradish-peroxidase-conjugated goat anti-human IgG antibodies (KPL, Socochim, Lausanne, Switzerland) (1∶2000 in PBS containing 0.05% Tween-20) for 1 h at 37°C and then washed. 1, 2-Diaminobezene substrate (OPD) (20 mg/tablet (Fluka, Sigma, Buchs, Switzerland)) in citrate-buffer (4 mg/ml OPD)+0.01% H_2_O_2_ was added and incubated at room temperature. After 10 minutes the reaction was stopped by addition of sulphuric acid (final concentration 0.5M (Merck, Darmstadt, Germany)). The optical density (OD) of the reaction product was recorded at 492 nm using a microplate reader (SpectraMax plus, Bucher Biotech, Basel, Switzerland). Titration curves were registered using Softmax PRO software. Endpoint titres were calculated by comparing the ELISA OD of the test serum with the ELISA OD of a negative serum pool. The endpoint titre is the last serum dilution where the OD_test sera_≥2×OD_negative serum_.

Avidity index (concentration of thiocyanate leading to the dissociation of 50% of the bound IgG in ELISA) was measured by adding serial dilutions of ammonium-thiocyanate after serum incubation (triplicates at half max saturation), leading to partial dissociation of bound antibodies [20]. Western blotting with a lysate of *P. falciparum* (strain NF54) infected *A. stephensi* salivary glands, or a lysate of *in vitro* cultivated *P. falciparum* (strain K1; schizont stage) blood stage parasites, was performed to measure anti-CS protein or anti-AMA-1 IgG seroconversion at a serum dilution of 1∶100.

Immunofluorescent antibody assays (IFA) were used to measure anti-parasite IgG endpoint titres (defined as last serum dilution where a staining of the parasite is visible). IFA with a suspension of *P. falciparum* (strain NF54) infected *A. stephensi* salivary glands and IFA with a suspension of synchronised *P. falciparum* (strain K1; schizont stage) infected red blood cells, was used to assess anti-sporozoite and anti-blood stage endpoint titres, respectively. Western blotting and IFA are described in more detail in [11], [12].

Positive and negative sera from a phase 1 trial with PEV301 and PEV302 [14] were used as controls for ELISA, IFA and Western blotting.

#### 3. Safety

The outcome of vaccine safety was assessed by collection of local and systemic adverse events. Each subject was observed for at least 30 minutes after vaccination, and underwent clinical review 2, 7 and 28 days after each vaccination for reporting of solicited and unsolicited adverse events. Subjects also completed a diary card every day for the first 7 days after each vaccination. Full blood count and biochemistry (urea, electrolytes, alanine aminotransferase, bilirubin, alkaline phosphatase, albumin) was performed at day 0, 7, 28, 35, 56, 63, day of challenge (day 77), challenge+7 days (day 84), challenge+35 days (day 112) and challenge+90 days (day 167).

#### Inhibition assays

Plasma samples were shipped to Carole Long at the NIH for parasite Growth Inhibition Assay (GIA) analysis as described elsewhere [21]. Briefly, polyclonal IgG was purified from plasma, adjusted to a concentration of 30.0mg/mL in incomplete RPMI 1640, and tested for biological activity against both the 3D7 and FVO strains of *Plasmodium falciparum*. Both strains are heterologous to those used in the construction of the vaccines–T9/96 in the ME TRAP construct, and K1 for PEV3A. A standardized GIA assay with samples from all volunteers on the day of challenge was performed and was compared with activity in samples from day 0.

#### Crisis Forms

Further analysis of blood films from each volunteer was undertaken. The blood film from the time of diagnosis, and the preceding five films were selected. One of the two microscopists examined each film in its entirety (1000 high power fields), recorded the number of parasites seen, and commented on their morphology. The microscopists were blinded to the group allocation of the volunteers during this study.

#### Sequencing

Parasite DNA was extracted from blood sample from volunteer 525 on day 20 as described [17]. Primers were designed to give a PCR product of 517 base pairs, which included the region coding for the AMA-1 peptide in PEV3A. The PCR products were purified and cloned into *E. coli* cells. DNA purified from five of these clones was sequenced.

### Sample size

This study aimed to provide both an initial estimate of the efficacy of the vaccines used in combination and to compare this with the single vaccine PEV3A. Prior to this trial, it was difficult to estimate the potential size of any beneficial effect of PEV3A. The analysis of overall efficacy in the 24 vaccinated volunteers had 80% power to detect a significant difference from the six controls in rate of sterile protection if 60% efficacy was achieved. Volunteers were allocated to groups by the investigators. Neither volunteers nor the investigators performing the study were blinded to the intervention given.

### Statistical methods

The main analysis for the primary objective was based on the number of hours between infectious challenge and blood stage parasitaemia. Each of the groups was compared with the other and with the control group using the Kaplan Meier method. Statistical significance of any differences observed was then assessed by the log rank test. Further analysis of parasite growth rates was undertaken using Mann Whitney U tests to compare groups. The secondary objective, safety and tolerability, is descriptive. Immunogenicity was assessed by comparing the geometric means of summed interferon-γ ELISPOT responses to malaria peptides. The geometric mean and 95% confidence intervals of the antibody titres determined by ELISA and IFA in the two different vaccination groups were calculated separately for each time point and study group. The statistical significance of the effect of combined vaccine delivery was determined using a Mann-Whitney U test to compare ELISA and IFA titres between the two vaccination groups. The impact of co-administration of vaccines on antibody avidity was calculated with a two-tailed unpaired t test comparing avidities after first, second, and third immunisation. The statistical significance of the difference in number of responders after three vaccinations in the two groups was calculated with a Fisher's exact test. To assess the statistical significance of titre increases (ELISA, IFA) after sporozoite challenge we used a Wilcoxon signed-rank test to compare titres before and after sporozoite challenge. Results were analysed for correlation to any delay in parasitaemia. All data, including adverse event data collected on StudyBuilder, were imported into and analysed using Microsoft Excel or SPSS statistics packages. All statistical analyses and graphs of ELISA, IFA and avidity results were made using GraphPad Prism version 4.03 for Windows, GraphPad Software, San Diego California USA.

## Results

### Participant flow

**Figure 2:**
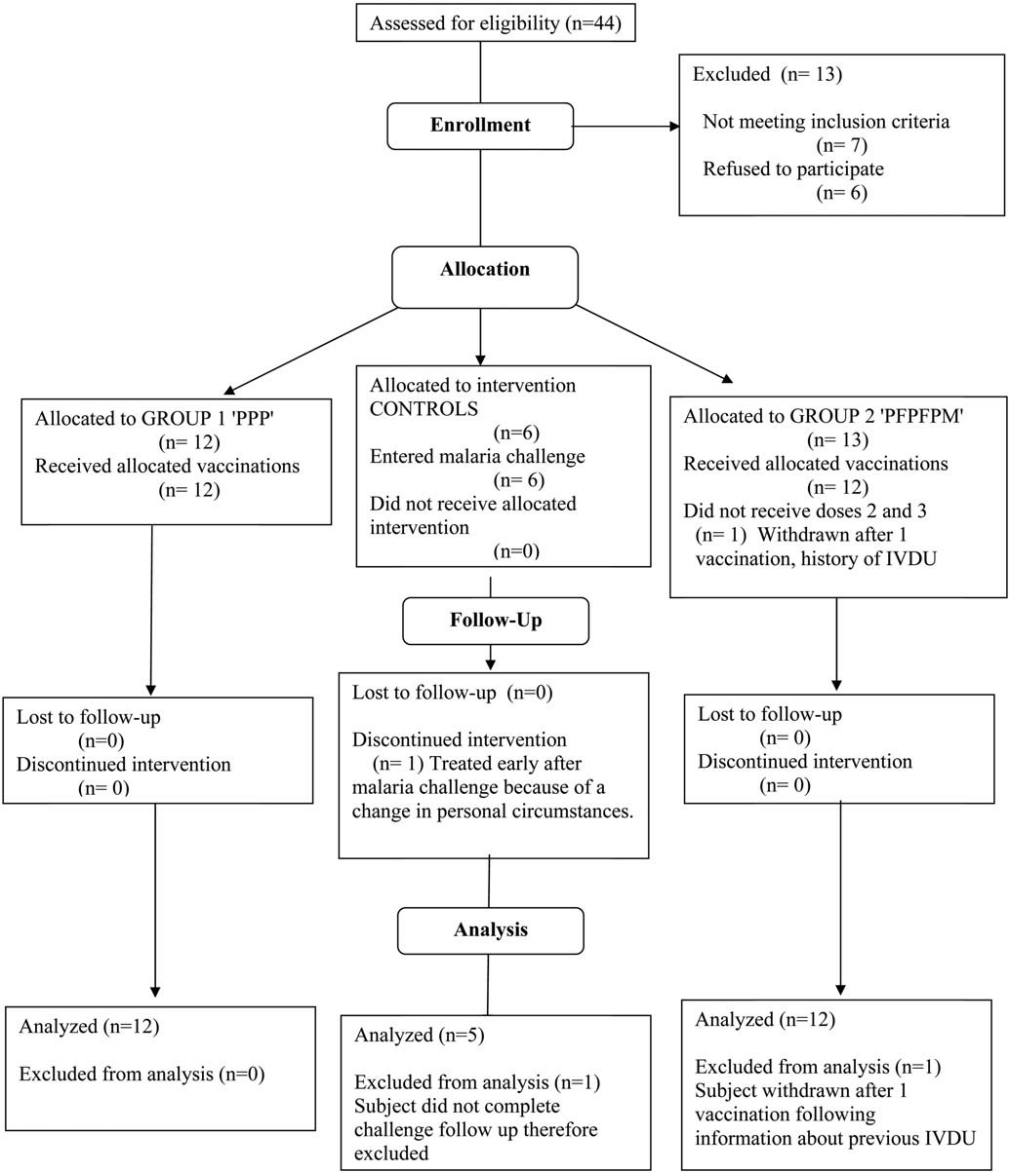
CONSORT Flow Chart

The participant flow is shown in the CONSORT flowchart in Figure 2. In total, 44 subjects were screened, of whom 13 were excluded, 7 because they were ineligible (previous history of intravenous drug use (IVDU), history of psychiatric illness, a new finding of heart murmur, recent travel to endemic areas), and 6 withdrew consent after screening. 30 volunteers were initially enrolled into the study. One of those enrolled into group 2 had an undisclosed history of IVDU; this subject was withdrawn as soon as this was revealed to the investigator, shortly after the first vaccination. Data concerning the safety of one dose of vaccine was collected from this volunteer, but they were otherwise excluded from the analysis. One extra volunteer who had been screened but initially not enrolled was then allocated to group 2 to replace this subject.

### Recruitment

Recruitment began in August 2005, and the final visit took place on 22^nd^ February 2006.

### Baseline data

Baseline demographic and clinical characteristics for all intervention groups at entry into the trial are shown in Table 1.

**Table 1:** Demographic characteristics

Group	N	Mean AGE (SD)	Min AGE	Max AGE	No. FEMALE (%)	No. MALE (%)
**Group 1**	12	25.8 (4.3)	21	33	6 (50)	6 (50)
**Group 2**	13	29.5 (5.3)	23	44	5 (38)	8 (62)
**Controls**	6	30 (7.5)	23	40	3 (50)	3 (50)
**TOTAL COHORT**	**31**		**21**	**44**	**14 (45)**	**17 (55)**

### Numbers analyzed

In total, 24 volunteers received all three vaccinations in groups 1 and 2. All of these volunteers subsequently took part in the malaria challenge, and completed all follow up. 6 of the enrolled volunteers were recruited to act as unvaccinated controls for the challenge phase. One of these subjects was withdrawn from the challenge early and treated; this was a result of an unforeseen change in personal circumstances unrelated to the trial. This volunteer subsequently completed all follow up. This subject, and the one withdrawn after the first vaccination, accounted for the two protocol deviations recorded during the trial.

### Outcomes and estimation

#### Vaccine Potency and Safety

The results of pre-trial potency testing revealed that both FP9 and MVA ME-TRAP were as immunogenic as expected based on previous assays of these vaccines, producing on ELISPOT a mean number of 125 spot forming units (sfu) per 10^6^ splenocytes (standard deviation, SD 59.7), and 554 sfu/10^6^ splenocytes (SD 118) respectively (mean of results from 4 individual mice for each vaccine).

There were no clinically significant changes in haematological (full blood count) or biochemical (sodium, potassium, urea, creatinine, ALT, alkaline phosphatase, albumin, bilirubin) parameters throughout the study. No serious adverse events occurred during this study. One volunteer developed an enlarged right supraclavicular lymph node following the first vaccination with FP9 ME-TRAP. The maximum recorded size of this lymph node was 1.3cm. It increased in size again following subsequent vaccinations in the right arm. This node was still present at study close out. The volunteer was referred to their General Practitioner for assessment and investigation, who has indicated that no further follow up is required. Adverse events following vaccination followed a profile similar to that seen before with these vaccines [15]. The frequency of vaccination site pain, swelling, redness, warmth, itch and scaling was, as expected, higher following the intradermal administration of FP9 and MVA ME-TRAP than with the intramuscular administration of PEV3A. One volunteer (N514, in group 1) reported severe pain following the third vaccination with PEV3A. This was recorded as starting on the evening of the third vaccination, and lasting 6 days. It was recorded as intensity 3 (severe pain at rest) for two days, and then subsided to intensity 2 (pain on movement) for a further two days. For the final two days it was recorded with a score of 1 (pain on touch). After this period it had completely resolved, with no long term effects. The frequency of general symptoms in group 1 was also lower than that in group 2. In group 1, 50% of volunteers experienced at least one systemic side effect (5/12 = 41% after dose 1, 1/12 = 8% after dose 2, and 2/12 = 17% after dose 3), whilst 83% (10/12) in group 2 experienced at least one (6/13 = 46% after dose 1, 7/12 = 58% after dose 2, 9/12 = 75% after dose 3). This is slightly different to previously reported studies using similar viral vectored vaccines, where general symptoms seemed to be attenuated following second and subsequent doses [15]. See tables 2 and 3 for details of adverse events considered related to vaccination.

**Table 2:** Frequency of solicited local symptoms after each vaccine dose

VACCINE DOSE 1	Group 1, n = 12		Group 2, n = 13			
	PEV3A i.m.		PEV3A i.m.		FP9 ME-TRAP i.d.	
	n	%	n	%	n	%
Pain	3	25	3	23	6	46
Redness	2	17	2	15	13	100
Swelling	2	17	1	8	11	85
Warmth	0	0	0	0	3	23
Itch	0	0	1	8	4	31
Scaling	0	0	0	0	7	54
**VACCINE DOSE 2**	**Group 1, n = 12**		**Group 2, n = 12**			
	**PEV3A i.m.**		**PEV3A i.m.**		**FP9 ME-TRAP i.d.**	
	**n**	**%**	**n**	**%**	**n**	**%**
Pain	5	42	7	58	9	75
Redness	2	17	0	0	12	100
Swelling	1	8	0	0	11	92
Warmth	0	0	0	0	1	8
Itch	0	0	0	0	5	42
Scaling	0	0	0	0	7	58
**VACCINE DOSE 3**	**Group 1, n = 12**		**Group 2, n = 12**			
	**PEV3A i.m.**		**PEV3A i.m.**		**MVA ME-TRAP i.d.**	
	**n**	**%**	**n**	**%**	**n**	**%**
Pain	8	67	8	67	12	100
Redness	2	17	2	17	12	100
Swelling	1	8	0	-	12	100
Warmth	0	-	0	-	10	83
Itch	0	-	0	-	8	67
Scaling	0	-	0	-	12	100

**Table 3:** Frequency of solicited general symptoms after each vaccine dose

VACCINE DOSE 1	Group 1, n = 12		Group 2, n = 13	
	n	%	n	%
Documented Fever >37.5	1	8	0	-
Symptoms of feverish	2	17	4	31
Malaise	0	-	4	31
Arthralgia	2	17	2	15
Headache	0	-	4	31
Myalgia	5	83	5	38
Nausea/vomiting	0	-	0	-
**VACCINE DOSE 2**	**Group 1, n = 12**		**Group 2, n = 12**	
	**n**	**%**	**n**	**%**
Document. Fever >37.5	0	-	0	-
Symptoms of feverish	1	8	4	33
Malaise	1	8	5	42
Arthralgia	1	8	1	8
Headache	0	-	3	25
Myalgia	3	25	6	50
Nausea/vomiting	0	-	1	8
**VACCINE DOSE 3**	**Group 1, n = 12**		**Group 2, n = 12**	
	**n**	**%**	**n**	**%**
Documented Fever >37.5	1	8	1	8
Symptoms of feverish	1	8	4	33
Malaise	1	8	5	42
Arthralgia	1	8	3	25
Headache	0	-	3	25
Myalgia	2	17	5	42
Nausea/vomiting	0	-	1	8

### Immunogenicity

#### 1. Antibody responses

**Figure 3:**
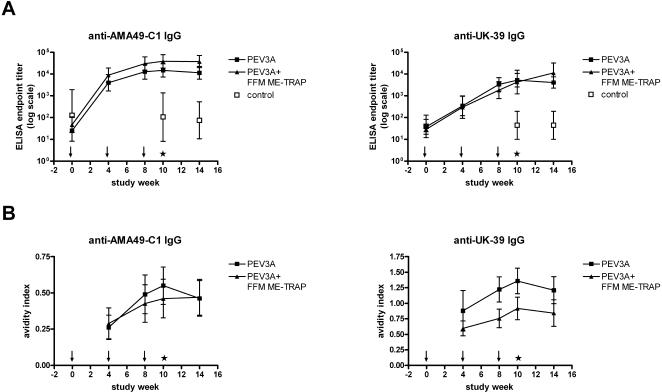
Anti-peptide responses. A. (Upper panel) Geometric mean anti-AMA49-C1 (PEV301) and anti UK-39 (PEV302) endpoint-titres (log10) with 95% confidence intervals for group 1 (PEV3A), group 2 (PEV3A+ME-TRAP) and unvaccinated controls. B. (Lower panel) Mean avidity increase (relative to avidity index after first immunisation) with 95% confidence intervals. Arrows along the x axis represent the timing of each vaccination, and the asterisk denotes the challenge.

**Figure 4:**
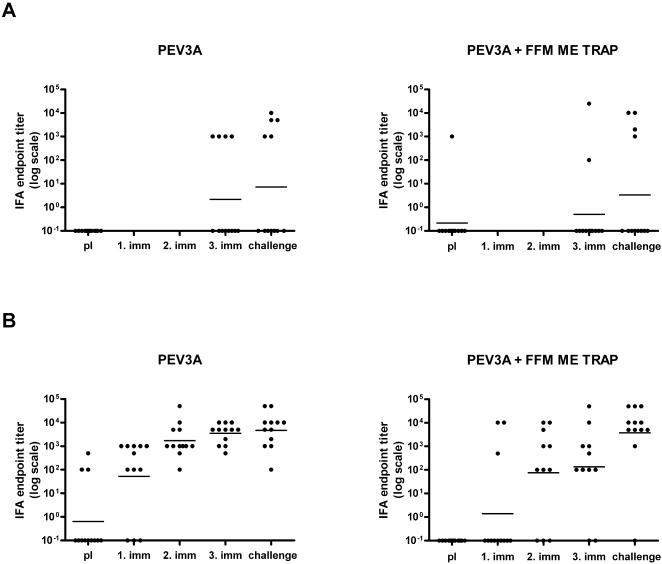
Antibody reactivity with *P. falciparum.* A. (Upper panel) IgG endpoint titres measured in IFA with *P. falciparum* (K1) blood stage parasites. B. (Lower panel) IgG endpoint titres measured in IFA with *P. falciparum* (NF54) sporozoites. Sera were tested before vaccination, after every vaccination and after sporozoite challenge. Individual titres and the geometric mean for every time point are shown on a log scale.

Anti-AMA49-C1 antibodies were induced at high levels in all volunteers following immunisation with PEV3A (Figure 3A). One immunisation was sufficient to produce 100% seroconversion in both groups. No increase in anti-peptide titres was observed after sporozoite infection. Unvaccinated controls did not show any increase in anti-AMA49-C1 antibody titres. Co-administration of FFM ME-TRAP led to an increase of anti-peptide IgG titres, which became significant after the third immunisation (Mann Whitney U; two-tailed p = 0.03). The avidity of this anti-peptide response increased following every vaccination (Figure 3B). The mean avidity index did not differ between the two vaccination groups at any time point, however the increase of avidity after three immunisations was significantly higher in the PEV3A group (two-tailed unpaired t test p = 0.03). Sporozoite challenge led to a decrease of avidity in group 1 (two-tailed paired t test p = 0.004), whereas no significant change was observed in the group receiving a combination of the two vaccines (two-tailed paired t test p = 0.6). Antibody reactivity with blood stage parasites was assessed by IFA with *P. falciparum* (strain K1) blood stage parasites (Figure 4A). Three immunisations with PEV3A led to an increase in parasite reactive IgG titres in 4/12 volunteers in group 1 and in 2/12 volunteers in group 2. Mean IgG titres in IFA after three immunisations did not significantly differ between the two groups (Mann-Whitney U two-tailed p = 0.49). There was no evidence of a difference in number of responders after three vaccinations between group 1 and 2 (Fisher's exact test p = 0.64). Although not statistically significant, we observed an increase in blood stage parasite-binding antibodies in IFA after sporozoite challenge. 78% (7/9) of all volunteers IFA positive after immunisation (both groups) had an increased endpoint titre after sporozoite infection. Unvaccinated controls did not produce detectable levels of blood stage parasite-binding antibodies (data not shown). Generally, interpretation of IFA results with blood stage parasites was very difficult due to non-specific background staining. As we considered only clearly positive results, we may have missed parasite-binding antibody responses in some volunteers. Western blot analysis with sera from immunised volunteers (after three vaccinations) showed specific recognition of parasite-derived AMA-1 in 16 out of 24 volunteers at a serum dilution of 1∶100 (data not shown) supporting the notion that we may have missed some parasite cross-reactive responses in IFA.

High levels of anti-UK-39 antibodies were detected after vaccination with PEV3A (Figure 3A). Two immunisations were required to achieve 100% seroconversion in both vaccination groups. Co-administration of FFM ME-TRAP had no impact on the magnitude of mean anti-UK-39 titres at any time-point (Mann-Whitney U two-tailed p = 0.95 after 3^rd^ vaccination). Sporozoite challenge led to an increase in anti-peptide titres in the combination group (Wilcoxon sign-rank p = 0.03), whereas no change in titre was seen in the PEV3A group after infection (Wilcoxon sign-rank p = 0.64), (Figure 3A). No anti-UK-39 IgG was detected in unvaccinated controls. The avidity of anti-UK-39 IgG increased following every immunisation (Figure 3B). Although there was a higher mean avidity index in group 1 compared to group 2 after second and third immunisation (two-tailed unpaired t test p = 0.001 after second and p = 0.008 after third vaccination) we did not observe any difference in avidity increase between the two vaccination groups (after 3^rd^ vaccine; two-tailed unpaired t test p = 0.6), or a significant change in avidity after sporozoite challenge (two-tailed paired t test group 1 p = 0.64; group 2 p = 0.44). Immunisation with PEV3A alone induced parasite cross-reactive antibodies in all volunteers after two vaccinations, as observed in IFA with *P. falciparum* sporozoites (Figure 4B). The number of volunteers with parasite-binding antibodies in the combination group increased with every immunisation reaching 82% (9/11) after the third immunisation. There was no significant difference in the number of responders after three vaccinations between group 1 and 2 (Fisher's exact test p = 0.22). Higher mean IFA titres induced by immunisation with PEV3A alone compared to combined vaccination became significant after three vaccinations (Mann-Whitney U two-tailed p = 0.02). A boost in vaccine-induced responses after infection was observed in 33% (4/12; group 1) and 75% (9/12; group 2) of the volunteers as detected by increased IFA titres after sporozoite challenge. The observed increase of IFA titres after infection was significant only for the group receiving both vaccines (Wilcoxon sign-rank p = 0.04). No sporozoite-binding antibodies were detected in unvaccinated controls after sporozoite challenge (data not shown). Western blot staining of a CSP characteristic double band was seen in 13 out of 23 sera following vaccinations at a dilution of 1∶100 (data not shown). There was no correlation in the level of any induced antibodies with time to infection.

#### 2. T cell responses

There were low level background responses to ME-TRAP in group 1 (summed response at peak time point was 13.3 SFU/10^6^ PBMC). Responses in group 2 were significantly higher than those in group 1. The geometric mean summed responses to ME and T996 TRAP pools at peak time point was 50 SFU/10^6^ PBMC (two-tailed t test on log converted data p = 0.001). However these responses were low compared to those seen in previous trials with this vaccine (previously, a geometric mean summed response to ME-TRAP of 454 SFU/10^6^ PBMC was observed at the peak time point).

### Efficacy

#### 1. Number of infected hepatocytes

**Figure 5:**
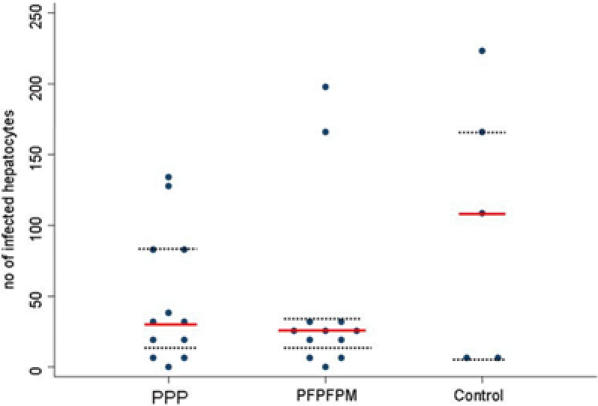
Number of infected hepatocytes. Estimated numbers of infected hepatocytes for individual volunteers are shown. Lines represent the geometric mean for each group with IQ ranges.

A model based on Hermsen et al. [18] allows the number of infected hepatocytes for each individual to be estimated. Compared to the control group the estimated number of infected hepatocytes was 1.8 times lower in the PEV3A group and 2.3 times lower in the combination group respectively (Figure 5). Statistical analysis of these estimates showed no significant difference between group 1 and group 2 (Mann Whitney U test, p = 0.6), or between either group of vaccinated volunteers and controls (group 1 versus controls, Mann Whitney U test, p = 0.3, group 2 versus controls, p = 0.4; all vaccinated volunteers versus controls, Mann Whitney U, p = 0.3).

#### 2. Development of parasitaemia

**Figure 6:**
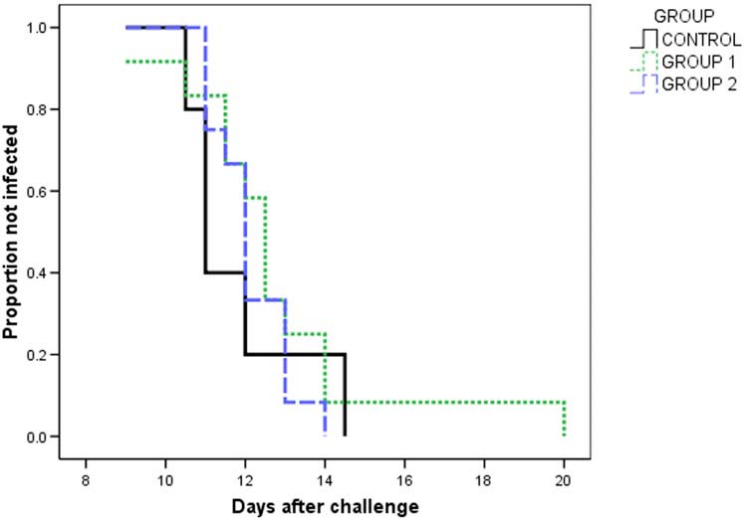
Kaplan Meier Survival curve. A Kaplan Meier plot showing time to diagnosis of malaria for each group of volunteers after the challenge.

Mean time to parasitaemia for controls (n = 5) was 11.8 days (S.D. 1.6 days), compared to 12.75 days for group 1 (n = 12, S.D. 2.68 days), and 12.1 days for group 2 (n = 12, (S.D. 0.96 days). A Kaplan Meier plot of survival is shown in Figure 6. One volunteer in group 1 was not diagnosed until day 20, however, there is no significant difference in time to parasitaemia for volunteers in either vaccination group compared to controls (Log rank = 0.87, p = 0.65).

**Figure 7:**
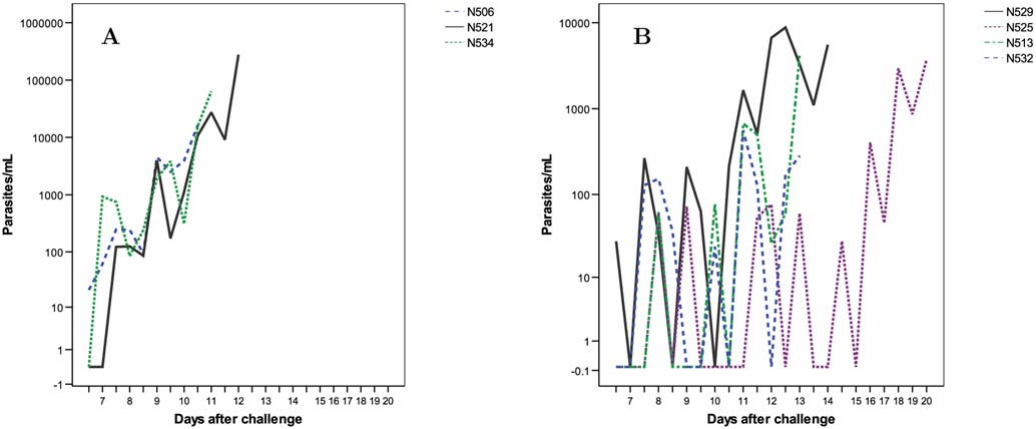
PCR data. Number of parasites per mL, estimated using a calibration curve, plotted by day post sporozoite challenge. A. Three volunteers selected to demonstrate expected pattern of PCR results: an exponential increase in the numbers of parasites over time, with some cycling seen as parasites are sequestered and released. B. This figure shows data from all 4 volunteers who had unusual PCR results. Up to 150 parasites/mL are detected, and are subsequently undetectable for one or more time points, before they are measured again. This pattern occurs several times, up to a maximum of 5 times in volunteer N525.

Examples of estimated parasite densities based on PCR data are shown in Figure 7. Most volunteers showed an exponential increase in parasite densities starting at day 7 after challenge (Figure 7A). However, a number of subjects (N525 and N529 in group 1 and N513 and N532 in group 2), had unusual PCR results (Figure 7B). In these volunteers PCR for parasite DNA was positive at low levels (known to be undetectable by microscopy) and was then negative before becoming positive again, at least once, before rising up to the point of diagnosis.

#### 3. Growth rates

**Figure 8:**
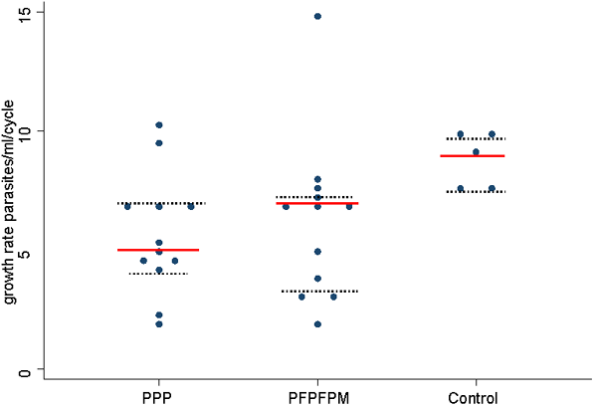
Growth rates, mean and IQ ranges, by group. Growth rate (parasites per mL per cycle) for each individual is shown; the lines represent the group means and inter quartile ranges.

The mean rate of blood stage parasite growth in volunteers in group 1 was 5.7 parasites per mL per cycle (95% confidence intervals 4.1–7.3; standard deviation 2.6), for group 2 this was 6.3 (95% CI 4.0–8.5; SD 3.5) and for controls it was 8.7 (95% CI 7.2–10.2; SD 1.2) (Figure 8). Comparing the growth rates of vaccinated volunteers, those in group 1 are not significantly different to those of volunteers in group 2 (Mann Whitney U test; two-tailed p = 0.63). Comparing group 1 to controls, growth rates are significantly lower in group 1 volunteers than in controls (Mann Whitney U; two-tailed p = 0.02). For group 2 versus controls, this is a significant reduction (Mann Whitney U; two-tailed p = 0.02). Grouping all vaccinated volunteers together, and comparing them to controls, there is again a significant reduction in parasite growth rates; (Mann Whitney U; two-tailed p = 0.012).

#### 4. Crisis forms

**Figure 9:**
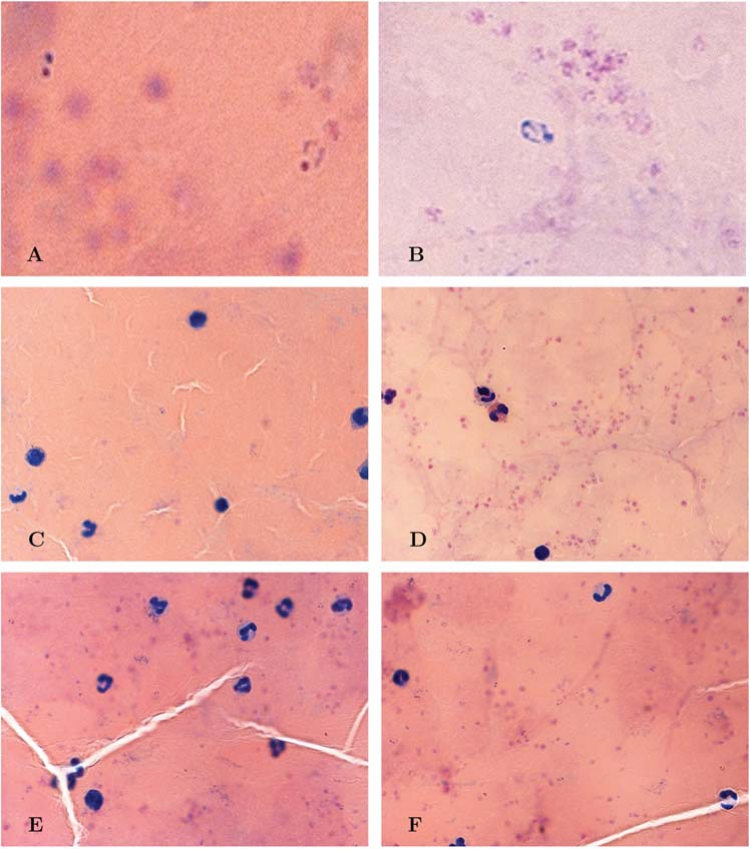
Normal parasites and crisis forms. Crisis forms differ from normal parasites in their slightly more ‘ragged’ morphology and altered staining of the nuclear material-live parasites have nuclei that stain red/blue, these abnormal parasites lose all red chromatin staining. Magnified high power images are shown in A&B. Normal parasites are seen in Figure 9A, whilst 9B shows a film containing a crisis form. The remaining films are unmagnified and white blood cell nuclei are clearly visible in blue, with smaller headphone shaped parasites. Figure 9C & D show single crisis forms, whilst 9E & F show multiple normal parasites, with the characteristic red/blue nuclear staining.

During the challenge phase, microscopists detected the presence of morphologically abnormal parasites in films from volunteers pre-diagnosis. These differ from normal parasites in the staining of the nuclear material. While live parasites have nuclei that stain red/blue, these abnormal parasites stained only blue (see Figure 9). We hypothesised that these were ‘crisis forms’ [22] and that they might represent an effect of vaccine-induced blood stage immunity. Morphologically abnormal parasites were seen in films from every vaccinated volunteer (n = 24) in this study. They were identified in the films of significantly fewer (2/6) controls (Fisher's exact test, p = 0.001). As a proportion of crisis forms among all parasites detected, in group 1, the average was 66% (95% CI, 54–78, SD 19), for group 2, the proportion was 55% (95% CI, 41–69, SD 22) and for controls the proportion was 13% (95% CI −11–37, SD 23). The proportion of crisis forms among all detected parasites in vaccinated volunteers was significantly greater than in controls (Mann Whitney U, p = 0.001).

### Ancillary analyses

#### 1. Growth Inhibition Assay

No significant GIA activity was seen in any sample relative to day 0 activity.

#### 2. AMA-1 sequencing

Volunteer 525 remained undiagnosed until day 20 after the challenge and then became blood film positive. The possibility that this could result from parasite immune escape was considered. Vaccination-induced mutations in another *Plasmodium* antigen have been described in monkeys after relatively short periods of infection [23]. We sequenced the AMA-1 gene from DNA extracted from a blood sample from volunteer 525 on the day of diagnosis (day 20) but found no sequence changes.

## Discussion

This trial is the first to provide evidence of vaccine-induced blood stage anti-parasite efficacy and partial protection in a healthy volunteer challenge study. Two observations are indicative of a parasite inhibitory effect.

The first of these may be divided into three components. (I.i) One volunteer was not diagnosed until day 20 after infection, this being a substantial delay in time to diagnosis. In 52 non-vaccinated control volunteers involved in identical challenge studies at the centre over the last six years, none have remained parasite free on microscopy beyond day 14 post challenge. (I.ii) The same volunteer, along with three others, had fluctuations in PCR-measured parasite densities that are consistent with a blood stage inhibitory effect on parasite growth. (I.iii) There was a significant difference in the parasite growth rates in vaccinated volunteers versus controls. This is observed in both groups of vaccinated volunteers, and is therefore likely to be related to vaccination with PEV3A.

The second observation relates to the presence of morphologically abnormal parasites suggests a vaccine-induced immune response against the parasites. Malaria parasite crisis forms were first described in *P. brasilianum* infection of Cebus monkeys 1944 by Taliaferro, WH et al. [22]. They have since been described in mouse [24] and human malarias [25]. A study by Jensen et al. [26] demonstrated induction of crisis forms in cultured *P. falciparum* with human immune serum from Sudan. Historically, these abnormal parasites have not been observed in studies at this centre before. However, as they were also observed in the blood of two of six control volunteers, their presence is not necessarily vaccine related.

The volunteer in whom diagnosis was delayed until day 20 developed high levels of anti AMA49 and UK39 antibodies (peak AMA49 response end point titre 33779, UK39 endpoint titre 18102). Positive IFA results were obtained with sporozoites, but not with blood stage parasites. ELISPOT responses were negligible, with AMA-1 and NANP responses all remaining <10 sfu/10^6^ PBMC throughout the study. No detectable GIA activity was measured.

Taken together these results suggest that PEV3A has induced a protective immune response against blood stage parasites. However this was not sufficient to prevent patent infection in malaria naïve individuals in the stringent challenge model used that involves high numbers of sporozoites for infection. Blood stage protection might be enhanced by targeting several blood stage antigens with a multivalent subunit vaccine and this is technically feasible with the virosomal vaccine approach.

This study was designed to search for a possible synergistic effect of combining two potentially complimentary vaccine strategies. The idea that combining induction of anti-sporozoite antibodies by the CS component of PEV3A and anti-liver-stage T cell responses by recombinant viruses would lead to a synergistic enhancement of protection was suggested by experiments in a mouse model [5]. In this model, viral vectors appeared to act as an adjuvant to a protein vaccine, enhancing the magnitude of the antibody response. The best results (i.e. highest levels of protection from a subsequent malaria challenge) were obtained when the vaccines were mixed physically in the same syringe and administered at the same site. Both T cells and antibodies were shown to be important in mediating this protection. In this first phase I/IIa trial, we elected to administer the vaccines at the same time, but at separate sites rather than mixed in the same syringe. This was in order to reduce the potential risk of interference of the two vaccines. The vaccines used were safe when administered concurrently, in opposite arms, and the PEV3A virosomes were immunogenic. High levels of anti-AMA49-C1 and anti-UK-39 antibodies were measured post vaccination in all volunteers. A high proportion of these antibodies were able to bind to the native parasite proteins *in vitro* in IFA suggesting they are likely to be functional. There was some discrepancy between IFA positivity and the results of Western blot analysis. This appears to be related to high levels of background staining in the IFA with blood stage parasites in many of the volunteers on day 0, probably caused by cross-reactive antibodies. The observed ELISA titres were comparable with those from a previous Phase I study using this vaccine [14]; (Okitsu et al. submitted). Co-administration of FFM ME-TRAP did boost the magnitude of the anti-AMA49-C1 antibody response but not the anti-UK-39 response. However, whereas in the animal model, good T cell induction was achieved by the viral vectored vaccines this was reduced about 10-fold in this study compared to previous trials. Co-administration of vaccines could have led to antigenic competition or interference, which might account for this low immunogenicity. Both FP9 and MVA ME-TRAP were tested for potency in mice prior to the study, and were found to be potent, and slightly more immunogenic than previously used batches of this vaccine (data not shown). Estimated numbers of *P. falciparum* infected hepatocytes were similar between both groups of vaccinated volunteers, leading to the conclusion that there was no clear evidence of any liver stage effect by FFM ME-TRAP, in keeping with the low T cell immunogenicity observed.

Another important observation in this study was an increase in parasite reactive antibodies after sporozoite challenge. While anti-peptide titres did not markedly change, titres measured in IFA with *P. falciparum* blood stage parasites and sporozoites were boosted by infection. This indicates that PEV3A-induced immune responses can be boosted and skewed towards parasite-binding antibody populations by sporozoite infection. Results from *in vitro* sporozoite inhibition assays in a phase 1 trial with the same vaccine have shown inhibition of sporozoite migration and invasion in the presence of anti-UK-39 IgG (Okitsu et al., submitted). Although statistically not significant, we found a trend towards reduced numbers of infected hepatocytes in both vaccination groups compared to unvaccinated controls, possibly suggesting a role for anti-UK39 antibodies in the reduction of infected hepatocytes, but there was little power in this inter-group comparison.

Reduced parasite growth rates and the presence of crisis forms in the blood of all vaccinated volunteers who took part in this trial provide evidence that the immune response to the vaccine PEV3A is exerting an anti-blood stage protective effect. Murine monoclonal antibodies against AMA49-C1 are capable of inhibiting blood stage parasite growth in vitro [11], which is at least in part due to inhibition of intra-erythrocytic parasite development (unpublished observation).

The interpretation of these results is complicated by the fact that AMA-1 is expressed by both sporozoites and merozoites [27], and so it is possible that antibodies to this antigen induced by the virosome might have contributed to any pre-erythrocytic effect. The non significant trend to reduced numbers of parasites emerging from the liver of all vaccinated volunteers may reflect this.

It might be argued that these observations of blood stage immunity (reduction in growth rates, presence of crisis forms etc.) may rather be related to the potential pre-erythrocytic action of PEV3A. However, in previous challenge studies, the number of parasites emerging from the liver of unvaccinated control volunteers has been shown to vary as much as five fold [7]. Despite this variation, rates of parasite growth in these volunteers were similar. Equally, crisis forms have never been observed historically in our studies of pre-erythrocytic vaccine candidates. Indeed, following their observation in this study, the same slide reader went back to examine a selection of blood films from vaccinated and control volunteers in two previous studies where some evidence of pre-erythrocytic efficacy has been observed (VAC021 [28] and VAC023 [29]). The slide reader was blinded to the group allocation of the volunteers. In total 72 slides were selected for re-examination (6 slides each from 6 volunteers from each study, 3 vaccinees plus 3 controls) and no crisis forms were observed. It seems likely therefore, that the differences observed here are indeed related to vaccine induced blood stage immunity.

The current challenge was a heterologous one with 3D7 parasites, rather than the K1, or T9/96 strains, used in the generation of PEV3A or the ME-TRAP vaccines respectively. PEV301 targets the semi-conserved sequence AMA-1^446-490^, which contains only three dimorphic positions (D/N^448^, M/K^451^ and K/I^485^) at its C and N terminus, respectively. The elicited antibody responses are focussed on the conserved central portion of this sequence stretch and all murine vaccine-induced monoclonal antibodies tested were cross-reactive with *P. falciparum* strains expressing natural sequence variants of AMA-1^446-490^ [11]. In contrast to results with monoclonal antibodies, growth inhibition assays performed with sera of the volunteers immunised in this trial were negative. Further data will be required to assess the utility and sensitivity of this assay for predicting blood stage vaccine efficacy, but these trial results suggest caution in the exclusive use of this assay as an *in vitro* predictor of blood-stage vaccine efficacy.

An effective malaria vaccine may need to target multiple parasite antigens from different stages of the life cycle. Several vaccine candidates have previously provided evidence of pre-erythrocytic stage efficacy in phase IIa trials [8], [30], [31], [32]. Here we have shown evidence for a vaccine-induced blood stage protection for the first time in a challenge study. This study also shows that protective efficacy at the blood-stage level can be observed using sporozoites rather than blood stage parasites [33] for challenge studies. Moreover, 100% seroconversion induced by virosomally-formulated peptides and the observed boost of this response by sporozoite infection support the potential of further development of the virosome system as a malaria vaccine.

## Supporting Information

Consent Form S1 (0.03 MB PDF)

Ethics Approval Letter S1 (0.45 MB PDF)

Checklist S1 CONSORT Checklist
(0.05 MB DOC)

Protocol S1 Trial Protocol
(0.78 MB PDF)

Addendum S1 Trial Protocol Addendum
(0.02 MB PDF)

## References

[1] Snow RW, Guerra CA, Noor AM, Myint HY, Hay SI (2005). The global distribution of clinical episodes of Plasmodium falciparum malaria.. Nature.

[2] Richie TL, Saul A (2002). Progress and challenges for malaria vaccines.. Nature.

[3] Miller LH, Howard RJ, Carter R, Good MF, Nussenzweig V (1986). Research toward malaria vaccines.. Science.

[4] Wang R, Charoenvit Y, Daly TM, Long CA, Corradin G (1996). Protective efficacy against malaria of a combination sporozoite and erythrocytic stage vaccine.. Immunol Lett.

[5] Hutchings CL, Birkett AJ, Moore AC, Hill AV (2007). Combination of protein and viral vaccines induces potent cellular and humoral immune responses and enhanced protection from murine malaria challenge.. Infect Immun.

[6] McConkey SJ, Reece WH, Moorthy VS, Webster D, Dunachie S (2003). Enhanced T-cell immunogenicity of plasmid DNA vaccines boosted by recombinant modified vaccinia virus Ankara in humans.. Nat Med.

[7] Bejon P, Andrews L, Andersen RF, Dunachie S, Webster D (2005). Calculation of liver-to-blood inocula, parasite growth rates, and preerythrocytic vaccine efficacy, from serial quantitative polymerase chain reaction studies of volunteers challenged with malaria sporozoites.. J Infect Dis.

[8] Webster DP, Dunachie S, Vuola JM, Berthoud T, Keating S (2005). Enhanced T cell-mediated protection against malaria in human challenges by using the recombinant poxviruses FP9 and modified vaccinia virus Ankara.. Proc Natl Acad Sci U S A.

[9] Moorthy VS, Imoukhuede EB, Keating S, Pinder M, Webster D (2004). Phase 1 evaluation of 3 highly immunogenic prime-boost regimens, including a 12-month reboosting vaccination, for malaria vaccination in Gambian men.. J Infect Dis.

[10] Bejon P, Mwacharo J, Kai O, Mwangi T, Milligan P (2006). A Phase 2b Randomised Trial of the Candidate Malaria Vaccines FP9 ME-TRAP and MVA ME-TRAP among Children in Kenya.. PLoS Clin Trials.

[11] Mueller MS, Renard A, Boato F, Vogel D, Naegeli M (2003). Induction of parasite growth-inhibitory antibodies by a virosomal formulation of a peptidomimetic of loop I from domain III of Plasmodium falciparum apical membrane antigen 1.. Infect Immun.

[12] Okitsu SL, Kienzl U, Moehle K, Silvie O, Peduzzi E (2007). Structure-activity-based design of a synthetic malaria peptide eliciting sporozoite inhibitory antibodies in a virosomal formulation.. Chem Biol.

[13] Gluck R, Mischler R, Brantschen S, Just M, Althaus B (1992). Immunopotentiating reconstituted influenza virus virosome vaccine delivery system for immunization against hepatitis A.. J Clin Invest.

[14] Genton B, Pluschke G, Degen L, Kammer AR, Westerfeld N (2007). A randomized placebo-controlled phase Ia malaria vaccine trial of two virosome-formulated synthetic peptides in healthy adult volunteers.. PLoS ONE.

[15] Webster DP, Dunachie S, McConkey S, Poulton I, Moore AC (2006). Safety of recombinant fowlpox strain FP9 and modified vaccinia virus Ankara vaccines against liver-stage P. falciparum malaria in non-immune volunteers.. Vaccine.

[16] Chulay JD, Schneider I, Cosgriff TM, Hoffman SL, Ballou WR (1986). Malaria transmitted to humans by mosquitoes infected from cultured Plasmodium falciparum.. Am J Trop Med Hyg.

[17] Andrews L, Andersen RF, Webster D, Dunachie S, Walther RM (2005). Quantitative real-time polymerase chain reaction for malaria diagnosis and its use in malaria vaccine clinical trials.. Am J Trop Med Hyg.

[18] Hermsen CC, de Vlas SJ, van Gemert GJ, Telgt DS, Verhage DF (2004). Testing vaccines in human experimental malaria: statistical analysis of parasitemia measured by a quantitative real-time polymerase chain reaction.. Am J Trop Med Hyg.

[19] Vuola JM, Keating S, Webster DP, Berthoud T, Dunachie S (2005). Differential immunogenicity of various heterologous prime-boost vaccine regimens using DNA and viral vectors in healthy volunteers.. J Immunol.

[20] Ferreira MU, Katzin AM (1995). The assessment of antibody affinity distribution by thiocyanate elution: a simple dose-response approach.. J Immunol Methods.

[21] Malkin EM, Diemert DJ, McArthur JH, Perreault JR, Miles AP (2005). Phase 1 clinical trial of apical membrane antigen 1: an asexual blood-stage vaccine for Plasmodium falciparum malaria.. Infect Immun.

[22] Taliaferro WT, LG (1944). The effect of immunity on the asexual reproduction of *Plasmodium brasilianum*.. The Journal of Infectious Diseases.

[23] Klotz FW, Hudson DE, Coon HG, Miller LH (1987). Vaccination-induced variation in the 140 kD merozoite surface antigen of Plasmodium knowlesi malaria.. J Exp Med.

[24] Clark IA, Virelizier JL, Carswell EA, Wood PR (1981). Possible importance of macrophage-derived mediators in acute malaria.. Infect Immun.

[25] Karunaweera ND, Carter R, Grau GE, Kwiatkowski D, Del Giudice G (1992). Tumour necrosis factor-dependent parasite-killing effects during paroxysms in non-immune Plasmodium vivax malaria patients.. Clin Exp Immunol.

[26] Jensen JB, Boland MT, Akood M (1982). Induction of crisis forms in cultured Plasmodium falciparum with human immune serum from Sudan.. Science.

[27] Florens L, Washburn MP, Raine JD, Anthony RM, Grainger M (2002). A proteomic view of the Plasmodium falciparum life cycle.. Nature.

[28] Dunachie SJ, Walther M, Epstein JE, Keating S, Berthoud T (2006). A DNA prime-modified vaccinia virus Ankara boost vaccine encoding thrombospondin-related adhesion protein but not circumsporozoite protein partially protects healthy malaria-naive adults against Plasmodium falciparum sporozoite challenge.. Infect Immun.

[29] Walther M, Thompson FM, Dunachie S, Keating S, Todryk S (2006). Safety, immunogenicity, and efficacy of prime-boost immunization with recombinant poxvirus FP9 and modified vaccinia virus Ankara encoding the full-length Plasmodium falciparum circumsporozoite protein.. Infect Immun.

[30] Dunachie SJ, Walther M, Vuola JM, Webster DP, Keating SM (2006). A clinical trial of prime-boost immunisation with the candidate malaria vaccines RTS,S/AS02A and MVA-CS.. Vaccine.

[31] Stoute JA, Slaoui M, Heppner DG, Momin P, Kester KE (1997). A preliminary evaluation of a recombinant circumsporozoite protein vaccine against Plasmodium falciparum malaria. RTS,S Malaria Vaccine Evaluation Group.. N Engl J Med.

[32] Kester KE, McKinney DA, Tornieporth N, Ockenhouse CF, Heppner DG (2001). Efficacy of recombinant circumsporozoite protein vaccine regimens against experimental Plasmodium falciparum malaria.. J Infect Dis.

[33] Pombo DJ, Lawrence G, Hirunpetcharat C, Rzepczyk C, Bryden M (2002). Immunity to malaria after administration of ultra-low doses of red cells infected with Plasmodium falciparum.. Lancet.

