# Correction: Evidence of Blood Stage Efficacy with a Virosomal Malaria Vaccine in a Phase IIa Clinical Trial

**DOI:** 10.1371/annotation/419b2f36-d46f-4f8a-8ffb-7fd2bfb33aa6

**Published:** 2011-02-26

**Authors:** Fiona M. Thompson, David W. Porter, Shinji L. Okitsu, Nicole Westerfeld, Denise Vogel, Stephen Todryk, Ian Poulton, Simon Correa, Claire Hutchings, Tamara Berthoud, Susanna Dunachie, Laura Andrews, Jack L. Williams, Robert Sinden, Sarah C. Gilbert, Gerd Pluschke, Rinaldo Zurbriggen, Adrian V. S. Hill

The following information was missing from the Funding section: "The study was supported by funding from the NIHR Oxford Biomedical Research Centre programme."

