# Supplementary material for: Evidence of Blood Stage Efficacy with a Virosomal Malaria Vaccine in a Phase IIa Clinical Trial
Source: PLoS One. 2008 Jan 30;3(1):e1493. doi: 10.1371/journal.pone.0001493 (PMC2204057; doi:10.1371/journal.pone.0001493)
Supplement: Consent Form S1 — (0.03 MB PDF) [file pone.0001493.s001.pdf]

CONSENT FORM

Title of Project: "Assessment of protection against malaria by sporozoite challenge of healthy adults vaccinated with the virosomal vaccine PEV3A and FP9-MVA ME-TRAP". VAC 030.

Names of Researchers: Adrian Hill, Fiona Thompson, David Porter

Oxfordshire REC number: 05/Q1604/69

Please circle answer

1. Have you read the Information Sheet Version 2, dated 9th May 2005? Yes/No
2. Have you had an opportunity to ask questions and discuss this study? Yes/No
3. Have you received satisfactory answers to your questions? Yes/No
4. Have you received enough information about the study? Yes/No
5. Who have you spoken to? Dr/Mr/Ms ___________________________
6. Do you understand that you are free to withdraw at any time, without giving any reason, without your medical care being affected? Yes/No
7. Do you agree to medical screening including testing for HIV, Hepatitis B and C? Yes/No
8. Do you give permission for regulatory authorities to have access to your records for the purposes of audit? Yes/No
9. Do you agree that we may have permission to contact your GP? Yes/No
10. Do you agree that some of your leftover blood samples will be stored and may be used for further studies of the body's immune response to malaria vaccination and malaria? Any such tests will have appropriate ethics committee approval. Samples will be stored for up to fifteen years and you have the right to ask us to dispose of your samples at any time. Your ability to participate in this study will not be affected by your decision to allow or not allow storage and future use of your leftover blood samples Yes/No
11. Do you understand that you may be given live, viral vaccines during this study? Yes/No/NA
12. If you wish to withdraw after you have been infected with malaria, do you understand that you must take a course of anti-malarial medication, and you will be asked to attend for 2 further days for safety reasons? Yes/No
13. Do you understand that should you fail to return for review as stated above, you may become seriously ill or die? Yes/No
14. Have you received enough information about the treatment schedule and potential side effects of Riamet®? Yes/No
15. Do you understand that participation in the trial will not allow you to donate blood in the UK for a period of at least 6 months? Yes/No
16. Women only: Do you understand the crucial need to use an effective method of birth control for the whole study **and** that you will be required to have pregnancy tests at regular intervals during this trial? Yes/No/NA

I HAVE READ THE INFORMATION SHEET AND HAVE UNDERSTOOD AND ANSWERED THE ABOVE QUESTIONS AND I CONSENT TO BEING A VOLUNTEER IN THIS STUDY

Signed: ____ **Date:** ___________________

Name: ____ **(in block letters)**

Signature of Investigator: ______________________________ **Date:** ___________________

Name of Investigator: ________________________________ **(in block letters)**
