# Supplementary material for: Evidence of Blood Stage Efficacy with a Virosomal Malaria Vaccine in a Phase IIa Clinical Trial
Source: PLoS One. 2008 Jan 30;3(1):e1493. doi: 10.1371/journal.pone.0001493 (PMC2204057; doi:10.1371/journal.pone.0001493)
Supplement: Protocol S1 — Trial Protocol (0.78 MB PDF) [file pone.0001493.s004.pdf]

CLINICAL TRIAL PROTOCOL

"Assessment of protection against malaria by sporozoite challenge of healthy adults vaccinated with the virosomal vaccine PEV3A and FP9-MVA ME-TRAP"

A Phase I / IIa controlled challenge trial

Oxford University Identifier: **VAC030**

Products: PEV3A, FP9 ME-TRAP and MVA ME-TRAP
Form: Liquid
Route: PEV3A – Intramuscular
FP9 ME-TRAP and MVA ME-TRAP – Intradermal
Dosages: PEV3A 0.5 mL (PEV301 50 μg, PEV302 10 μg)
FP9 ME-TRAP 1 × 10^8 pfu
MVA ME-TRAP 1.5 × 10^8 pfu

Trial Centre: Centre for Clinical Vaccinology and Tropical Medicine (CCVTM)
Old Road, Headington, Oxford, OX3 7LJ, UK

Principal Investigator: **Adrian V. S. Hill**
Professor of Human Genetics
University of Oxford
Wellcome Trust Centre for Human Genetics
Roosevelt Drive, Oxford, OX3 7BN, UK
Tel: +44 1865 287759
Fax: +44 1865 287686
Email: adrian.hill@well.ox.ac.uk

Lead Investigator(s): **Fiona Thompson**
University of Oxford
CCVTM
Old Road, Headington, Oxford, OX3 7LJ, UK
Tel: +44 1865 857418
Fax : +44 1865 857471
Email: fiona.thompson@ndm.ox.ac.uk

Co-Investigator(s): **David Porter**
University of Oxford
Centre for Clinical Vaccinology & Tropical Medicine (CCVTM)
Old Road, Headington, Oxford OX3 7LJ, UK
Tel: +44 1865 857419
Fax: +44 1865 857471
Email: david.porter@ndm.ox.ac.uk

Susie Dunachie
University of Oxford
Centre for Clinical Vaccinology & Tropical Medicine (CCVTM)
Old Road, Headington, Oxford OX3 7LJ, UK
Tel +44 1865 857444
Email : Susie.dunachie@ndm.ox.ac.uk

Rinaldo Zurbriggen
Chief Scientific Officer
Pevion Biotech Ltd.,
Rehhagstrasse 79, CH-3018 Bern, Switzerland.
Tel: +41 31 9806417
Email: rinaldo.zurbriggen@pevion.com

Gerd Pluschke
Professor of Molecular Immunology
Swiss Tropical Institute
Socinstrasse 57, PO Box CH-4002, Basle, Switzerland
Tel +41 81 2848235
Email: gerd.pluschke@unibas.ch

Clinical Research Nurse:

Ian Poulton
University of Oxford
CCVTM
Old Road, Headington, OX3 7LJ, UK
E-mail: ian.poulton@well.ox.ac.uk
Tel : +44 1865 857401
Fax : +44 1865 857471

Project Manager:

Trudie Lang
University of Oxford
CCVTM
Old Road, Headington, Oxford, OX3 7LJ, UK
Tel: +44 1865 857382
Email: trudie.lang@ndm.ox.ac.uk

Local Safety Monitor:

David A Warrell
Nuffield Department of Clinical Medicine, University of Oxford
John Radcliffe Hospital, Oxford, OX3 9DU, UK
Tel: +44 1865 221332 / 220968
Fax: +44 1865 220984
E-mail: david.warrell@ndm.ox.ac.uk

Biological Evaluator:

Stephen Todryk
University of Oxford
CCVTM
Old Road, Headington, Oxford, OX3 7LJ

Research Scientist:

Sheila Keating
University of Oxford
CCVTM
Old Road, Headington, Oxford, OX3 7LJ, UK
E-mail: sheila.keating@ndm.ox.ac.uk

Sponsor:

University of Oxford
Named contact: Prof. Peter Ratcliffe
Nuffield Department of Medicine
Level 7, John Radcliffe Hospital, Oxford, OX3 9DU
Tel: +44 1865 857621
Fax: +44 1865 287533
E-mail: peter.ratcliffe@ndm.ox.ac.uk

Protocol Pharmacist:

Rhoda Walsh
The Pharmacy
John Radcliffe Hospital, Oxford, OX3 9DU, UK
Tel: +44 1865 221934
Pager: 4069

Collaborators (Challenge strain):

Walter Reed Army Institute of Research
Jackie L. Williams, PhD
Chief, Malaria Transmission Laboratory
Department of Entomology
Division of Communicable Diseases and Immunology
503 Robert Grant Avenue, Room 1W30, Bldg. 503
Silver Spring MD 20910-7500, U.S.A
Tel: +1 301 319 9557
E-mail: jackie.williams@na.amedd.army.mil

Collaborators

Imperial College
Robert Sinden
Professor of Parasite Cell Biology
Infection and Immunity Section
Biology Department
Imperial College, London
Tel: +44 20 7594 5425
E-mail: r.sinden@ic.ac.uk

Monitor:

Ceri McKenna
Appledown Clinical Research Ltd.
67, Gregories Road, Beaconsfield, Bucks. HP9 1HL.
Tel/Fax: +44 1494 677447
E-mail: ceri.appledown@virgin.net

Version : 3.2
Date : 08/11/2006~~01/06/2006~~~~15/09/2005~~

PROTOCOL SIGNATURE SHEET

Principal Investigator: ________________________________ Date: _____________

ADRIAN HILL
Professor of Human Genetics, University of Oxford

Pevion Biotech: ________________________________ Date: _____________

RINALDO ZURBRIGGEN
Chief Scientific Officer, Pevion Biotech Ltd.

PROTOCOL AMMENDMENTS

VERSION 2

1. 4/5/2005 CO-INVESTIGATORS Dr. Rinaldo Zurbriggen and Professor Gerd Pluschke added as co-investigators for study.

2. 4/5/2005 SECTION 9.2.2 Solicited local and general adverse events listed changed. Limitation of arm movement and rash deleted from solicited local adverse event list. Following evaluation of recent study (VAC028) these adverse events do not occur commonly after vaccination, and will therefore not be routinely asked about. They will be recorded as adverse events if they occur.

3. 4/5/2005 SECTION 9.2.3 and APPENDIX C Safety criteria assessment will take place 30 minutes following vaccination as stated elsewhere in the protocol.

VERSION 3

4. 29/6/2005 Vaccination flow chart page xiv Post dose follow up at visit 11, day 77 is 21 days after the final vaccination, not 28 as was stated. This error has been corrected. The window for this visit has been changed to ±10 days rather than 7.

5. 29/6/2005 Vaccination flow chart page xiv. Blood taken at visit 10, D63 for gene expression. It is planned to take 10mL of blood for gene expression on this day. The flow chart was incorrect and has been amended. This brings the total volume of blood taken to 766 mL for group 1 and 3, and 774 mL for group 3. Where blood volume is mentioned elsewhere in the text, it has also been updated.

6. 29/6/2005 Challenge flow chart page xvi. The wording of the additional text below has been amended for clarification.

7. 29/6/2005 SECTION 5.5.1 The template label provided has been amended.

8. 29/6/2005 Challenge follow-up. The time window of the safety follow up visit on day 35 post challenge has been extended to ±10 days. This is because of the timing of the planned challenge, as some of the day 35 visits would fall over the Christmas holiday period, and volunteers would be unlikely to attend. As this is primarily a safety visit, the exact timing is less important than ensuring the maximum number of volunteers are able to attend.

9. 4/7/2005 Page x The name for MVA is Modified Virus Ankara, not Modified vaccinia virus Ankara as previously written. This has been corrected.

10. 4/7/2005 Page xi the batch number of PEV3A is now available – this has been added

11. 4/7/2005 Vaccines stored in pharmacy will be stored at less than -18°C, not -20 as previously stated.

VERSION 3.1

12. 3/9/2005 Where possible, PEV3A vaccines will be given into the left arm, and FP9/MVA ME-TRAP vaccines will be given into the right arm, as stated in the protocol. However, if volunteers have a specific reason, they may request vaccines be given in the other arm – ie PEV3A into the right arm and FP9/MVA ME-TRAP into the left. Subsequent doses of the same vaccine will be given at the same site.

VERSION 3.2

13. 15/9/2005 Appendix C Investigator SOPs, Page 48. The procedure for the visits following the challenge has been amended to state explicitly the intention to visit volunteers at home if they are unwell as a result of malaria infection.

VERSION 3.3

14. 1/6/2006 SECTION 9.2.7 page 34. Gene expression studies. The wording in the second sentence has been changed, 'will' has been corrected to 'may'. The amended sentence reads 'Whole genome high-density arrays **may** be used to compare gene expression at baseline and after vaccination…

Formatted: Bullets and Numbering

Formatted: Font: Bold

TABLE OF CONTENTS

Table of Contents vii

Synopsis x

Flow Charts: 1. Vaccination Attendances xiv

Flow Charts: 2. Challenge Visits xvi

Abbreviations and Symbols Used xvii

1 Introduction 1

1.1 Background 1
1.2 Vaccine vectors 3
1.3 Summary of previous clinical trials 6

2 Trial Objectives 9

2.1 Primary Objective 9
2.2 Secondary Objectives 9
2.3 Tertiary Objectives 9

3 Trial Design and Methodology 10

3.1 Trial design 10
3.2 Trial Plan 11
3.3 Case Report Forms and Data Collection 15
3.4 Procedures for Obtaining, Handling and Shipment of Biological Samples 15

4 Selection and Withdrawal of Subjects 17

4.1 Inclusion Criteria 17
4.2 Exclusion Criteria 17
4.3 Re-vaccination exclusion criteria 18

5 Vaccines 19

5.1 Investigational Vaccine Characteristics: FP9 and MVA ME-TRAP 19
5.2 Investigational Vaccine Characteristics: PEV3A 19
5.3 Vaccination 20
5.4 Prior and Concomitant Therapy 20
5.5 Management of Vaccines 21

6 Challenge Phase 22

6.1 Biting Procedure with *P. falciparum*-Infected Anopheles Mosquitoes 22
6.2 Anopheles Strain, Origin and Culture Conditions 22
6.3 *Plasmodium* Strain, Origin and Culture Conditions 22
6.4 Anopheles Infection and Storage 23
6.5 Toxicity Management 23

7 Management of Malaria Cases 24

7.1 Malaria Assessment 24
7.2 Treatment 24

7.3 Criteria for Hospital Admission to the John Warin (Infectious Diseases) Ward 24

8 Adverse event Management and reporting 26

8.1 Definitions 26
8.2 Safety Data Collection and Management Procedures 27
8.3 Reporting of Serious Adverse Events (SAE) 28
8.4 Post-trial Evaluations 29
8.5 Pregnancy 29

9 Evaluation Criteria 30

9.1 Primary Evaluation Criteria 30
9.2 Secondary Evaluation Criteria 31
9.3 Tertiary Evaluation Criteria 34

10 Biometry 35

10.1 Determination of the Sample Size 35
10.2 Data Set to be analysed 35

11 Ethical Consideration 37

11.1 Informed Consent 37
11.2 Subject Benefit and Risks 37
11.3 Ethical Review 37
11.4 Good Clinical Practice 37

12 Regulatory 38

13 Quality Control and Quality Assurance 39

13.1 Direct Access to Source Data/Documents 39
13.2 Modification of the Protocol 39
13.3 Termination of the Trial 39
13.4 Investigator Procedures 40
13.5 Monitoring 40
13.6 Audits and Inspections 40
13.7 Archiving 41

14 Financing and Insurance 42

14.1 Compensation for participation 42
14.2 Insurance 43

APPENDIX A : Laboratory Values for Exclusion 44

APPENDIX B : Text for radio advertisement 45

APPENDIX C : Investigator SOPs 46

C.1 Visit Procedures 46
C.2 Investigator Archiving 50
C.3 Communication with Ethics Committee and National Agency 50
C.4 Screening and recruitment of study subjects 50
C.5 SOP for obtaining informed consent 51
C.6 SOP on Suspected drug users 51
C.7 SOP on data handling 51
C.8 SOP for Preparation and Feeding of Infected Mosquitoes at Imperial College 52
C.9 SOP for Thick Film Preparation 53
C.10 SOP for work involving genetically modified organisms 53
C.11 SOP on Vaccine Handling 55

C.12 SOP Title Procedures in the event of accidental exposure to GMOs 57
C.13 SOP on decontamination 59
C.14 SOP on reporting of incidents 60
C.15 SOP Title Deactivation of GMO waste by autoclaving 61
C.16 SOP title Multiple use of vaccine vials containing more than a single dose 62
C.17 SOP on protocol compliance 62

Bibliographical References 64

SYNOPSIS

**Finished products**	PEV3A<br>MVA ME-TRAP<br>FP9 ME-TRAP
**Active ingredient**	PEV3A: Immunopotentiating reconstituted influenza virosome (IRIV) with malarial circumsporozoite protein (CSP) and apical membrane antigen-1 (AMA-1) synthetic peptides.<br>Fowlpox 9 (FP9) and Modified Virus Ankara (MVA) encoding Multi-Epitope (ME) string plus Thrombospondin-Related Adhesion Protein (TRAP)
**Trial Title**	"Assessment of protection against malaria by sporozoite challenge of healthy adults vaccinated with the virosomal vaccine PEV3A and FP9-MVA ME-TRAP"
**Trial Identifier**	VAC030
**Clinical phase**	I / IIa
**Principal Investigator**	Prof. Adrian V. S. Hill<br>University of Oxford
**Lead Investigator**	Dr. Fiona Thompson<br>University of Oxford
**Trial Centre**	Centre for Clinical Vaccinology and Tropical Medicine (CCVTM)<br>Old Road, Headington, Oxford, OX3 7LJ, UK
**Planned Trial Period**	May 2005 to November 2006
**Primary Objective**	To examine the efficacy of PEV3A alone and PEV3A combined with FP9-MVA ME-TRAP in a malaria challenge
**Secondary Objectives**	To confirm the safety and immunogenicity of administration of PEV3A and FP9-MVA ME-TRAP to healthy volunteers
**Tertiary Objectives**	To assess long term efficacy of PEV3A alone and PEV3A with FP9-MVA ME-TRAP in a late malaria challenge of volunteers protected at initial challenge.
**Trial Design**	Controlled clinical trial
**Planned Sample Size**	Group 1: 12 subjects – 3 doses of PEV3A at 0, 4 and 8 weeks<br>Group 2: 12 subjects – As group 1 **and** 2 doses of FP9 ME-TRAP at 0 and 4 weeks, followed by 1 dose of MVA ME-TRAP at 8 weeks<br>Group 3: 12 subjects – As group 1 **and** 2 doses of FP9 ME-TRAP at 1 and 5 weeks, followed by 1 dose of MVA ME-TRAP at 9 weeks<br>Control: 6 subjects for each challenge phase – These volunteers will not receive any vaccinations, but will act as controls to prove the infective efficacy of the challenge system.<br>**Total:** **48 volunteers.** All groups will subsequently undergo malaria challenge.
**Inclusion Criteria**	Healthy adults aged 18 to 50 years<br>Resident in or near Oxford for the duration of the vaccination study<br>For females only, willingness to practice continuous effective contraception during the study and, if participating, during the subsequent challenge study.<br>Agreement to refrain from blood donation during the course of the study<br>Written informed consent<br>Willingness to undergo an HIV test
**Exclusion Criteria**	Any deviation from the normal range in biochemistry or haematology blood tests or in urine analysis as defined in Appendix A.

**Exclusion Criteria (cont'd)**	Prior receipt of an investigational malaria vaccine<br>Use of any investigational or non-registered drug, vaccine or medical device other than the study vaccine within 30 days preceding dosing of study vaccine, or planned use during the study period<br>Administration of chronic (defined as more than 14 days) immunosuppressive drugs or other immune modifying drugs within six months of vaccination. (For corticosteroids this will mean prednisolone (or equivalent) ≥ 0.5 mg/kg/day. Inhaled and topical steroids are allowed.)<br>History of malaria chemoprophylaxis with chloroquine within 5 months prior to the planned challenge, with Lariam within 6 weeks prior to the challenge and Riamet® within 2 weeks prior to the challenge<br>Any history of malaria<br>Travel to a malaria endemic country within the previous 6 months<br>Planned travel to malarious areas during active vaccination and challenge follow up (ie from D0 to D 167)<br>Any confirmed or suspected immunosuppressive or immunodeficient condition, including human immunodeficiency virus (HIV) infection and asplenia<br>History of allergic disease or reactions likely to be exacerbated by any component of the vaccine, *e.g.* egg products<br>History of cancer (except basal cell carcinoma of the skin and cervical carcinoma in situ)<br>History of haemoglobinopathies: sickle cell disease, thalassaemia, G6PD deficiency<br>History of diabetes mellitus<br>Chronic or active neurological disease including seizures<br>History of ≥ 2 hospitalisations for invasive bacterial infections (pneumonia, meningitis)<br>Suspected or known current alcohol abuse as defined by an alcohol intake of greater than 42 units every week<br>Seropositive for hepatitis B surface antigen (HBsAg)<br>Seropositive for hepatitis C virus (antibodies to HCV)<br>Hepatomegaly, right upper quadrant abdominal pain or tenderness<br>Evidence of serious psychiatric condition<br>Any on-going chronic illness requiring hospital specialist supervision<br>Administration of immunoglobulins and/or any blood products within the three months preceding the planned administration of the vaccine candidate<br>Pregnant or lactating woman<br>Woman who is willing or intends to become pregnant during the study<br>Any history of anaphylaxis in reaction to vaccination<br>Lead Investigator assessment of lack of willingness to comply with all requirements of the protocol<br>History or clinical evidence of intravenous drug abuse see Appendix C.6.<br>Any other finding which in the opinion of the investigator would significantly increase the risk of having an adverse outcome from participating in this protocol
**Investigational Product**	PEV3A
Form	Liquid
Dose	PEV3A 0.5 mL (PEV301 50 μg, PEV302 10 μg)
Route	Intramuscular injection in the deltoid region of the upper arm
Lot Number	05PEV01
**Investigational Product**	MVA ME-TRAP
Form	Liquid
Dose	1.5×10^8 pfu
Route	Intradermal injection over the deltoid region of the arm
Lot Number	05 12 04

**Investigational Product**	FP9 ME-TRAP
Form	Liquid
Dose	1 × 10^8 pfu
Route	Intradermal injection over the deltoid region of the arm
Lot Number	05 12 04
**Vaccination Schedule**	Group 1: 3 doses of PEV3A at 0, 4 and 8 weeks<br>Group 2: As group 1 **and** 2 doses of FP9 ME-TRAP at 0 and 4 weeks, followed by 1 dose of MVA ME-TRAP at 8 weeks<br>Group 3: As group 1 **and** 2 doses of FP9 ME-TRAP at 1 and 5 weeks, followed by 1 dose of MVA ME-TRAP at 9 weeks<br>Control: No vaccinations
**Follow-up duration**	Vaccination groups: 167 days – with possible extension to 1 year 6 months if protected volunteers choose to participate in a late challenge (see Section 3.1.1)<br>Control group: 90 days (challenge phase alone)<br>*Note:* follow up duration is an estimate and may vary in accordance with the specified time windows for each attendance.
**Blood Sampling Schedule**	Groups 1 & 2: S, D0, D7, D28, D35, D56, D63, D77, D83, twice daily from D84 – D91, once daily from D92 - D100, D112, and D167. Total of 766 mL.<br>Group 3: S, D0, D7, D14, D28, D35, D42, D56, D63, D70, D77, D83, twice daily from D84 – D91, once daily from D92 - D100, D112, and D167. Total of 784 mL.<br>Control group: S, D77, D83, twice daily from D84 – D91, once daily from D92 - D100, D112, and D167. Total of 380 mL.
**Primary Evaluation Criteria**	• The number of subjects who develop malaria infection<br>• The time in hours between exposure and parasitaemia as detected by thick-film blood smear
**Secondary Evaluation Criteria**	The safety profile of the vaccines will be assessed on the following criteria<br>• Immediate reactogenicity (reactions within 30 minutes after each injection, with emphasis on allergic reaction),<br>• Local and systemic reactogenicity measured from day 0 to day 28 after each dose<br>• Any unsolicited adverse event resulting in a visit to a physician between each injection and one month after the third dose.<br>• Any Serious Adverse Event (SAE) occurring from the inclusion through out the study. The relationship of the adverse event to the study vaccine will be established by the investigator, using the following definitions : definitely related, probably related, possibly related or not related<br>• Biological safety, one week and four weeks after each vaccination, in reference with the baseline before the first dose, by measuring the following : Full blood count (FBC), Urea, Potassium, sodium, ALT, total bilirubin, alkaline phosphatase, albumin, creatinine<br>Immunogenicity of PEV3A and FP9-MVA ME-TRAP will be assessed by<br>• IFN-γ ELISPOT assays<br>• Antibodies against CS and AMA-1<br>• Gene expression studies
**Tertiary Evaluation Criteria**	Long term efficacy will be assessed by a second malaria challenge in protected volunteers as before by:<br>• The number of subjects who develop malaria infection<br>• The time in hours between exposure and parasitaemia as detected by thick-film blood smear

**Statistical Methods**	The main analysis for the primary objective will be based on the number of hours between infectious challenge and blood stage parasitaemia. The groups will be compared using the Kaplan Meier Method with log rank test of significance.

FLOW CHARTS: 1. VACCINATION ATTENDANCES

GROUPS ONE AND TWO

**Attendance number**	**1**	**2**	**3**	**4**	**5**	**6**	**7**	**8**	**9**	**10**	**11**
**Timeline (days)***	S	D0	D2	D7	D28	D30	D35	D56	D58	D63	D77
**Time windows (days)**			±1	±3	±7	±1	±3	+28/ -14†	±1	±3	±10
**Vaccinations (groups 1 AND 2)** (P=PEV3A)		P			P			P			
**Vaccinations (group 2 ONLY)** (F=FP9, M=MVA)		F			F			M			
**Attendance ref.**	**S**	**P1**	**P1A**	**P1B**	**P2**	**P2A**	**P2B**	**P3**	**P3A**	**P3B**	**P3C**
**Inclusion/Exclusion criteria**	X										
**Informed consent**	X										
**Medical history**	X										
**Phys. exam.**	X										
**Urinalysis**	X										
**β-HCG urine test**	X	X			X			X			
**Review contra-indications**	X	X			X			X			
**Post-dose follow-up (days)**			2	7	28	2	7	28	2	7	21
**Vital signs**	X	X	X	X	X	X	X	X	X	X	X
**Local & systemic events/reactions**		X	X	X	X	X	X	X	X	X	X
**Diary cards provided**		1			2			3			
**Diary cards collected**				1			2			3	
**HLA typing (mL)**		3									
**HBV, HCV, HIV (mL)**	5										
**Biochemistry (mL)**	4			4	4		4	4		4	4
**Haematology (mL)**	2			2	2		2	2		2	2
**Antibody assays (mL)**		10			10			10			10
**Gene expression (mL)**		10								10	10
**Exploratory immunology (incl ELISPOT) (mL)**		50		50	50		50	50		50	50
**Blood volume (mL)**	**11**	**73**		**56**	**66**		**56**	**66**		**66**	**76**
**Cumulative blood volume (mL)**	**11**	**84**		**140**	**206**		**262**	**328**		**394**	**470**

* Timeline is approximate only. Exact timings of visits relate to the previous visit – *i.e.* each visit must fall the specified number of days after the last visit ± time window

† Timing of the subsequent malaria challenge is not precise, due to variability in parasite culture. The time window for this last vaccination is therefore wider to ensure that this vaccination will precede the day of challenge by 21 ± 7 days

Grey columns highlight vaccination days

Number of visits : 11. Number of vaccinations : 3 (group 1) or 6 (group 2).

GROUP THREE

**Attendance number**	**1**	**2**	**3**	**4**	**5**	**6**	**7**	**8**	**9**	**10**	**11**	**12**	**13**	**14**	**15**	**16**	**17**
**Timeline (days)***	S	D0	D2	D7	D9	D14	D28	D30	D35	D37	D42	D56	D58	D63	D65	D70	D77
**Time windows (days)**			±1	±3	±1	±3	±7	±1	±3	±1	±3	+28/ -14†	±1	±3	±1	±3	±7
**Vaccinations** (P=PEV3A, F=FP9, M=MVA)		P		F			P		F			P		M			
**Attendance ref.**	**S**	**P1**	**P1A**	**F1**	**F1A**	**F1B**	**P2**	**P2A**	**F2**	**F2A**	**F2B**	**P3**	**P3A**	**M1**	**M1A**	**M1B**	**M1C**
**Inclusion/Exclusion criteria**	X																
**Informed consent**	X																
**Medical history**	X																
**Phys. exam.**	X																
**Urinalysis**	X																
**β-HCG urine test**	X	X		X			X		X			X		X			
**Review contra-indications**	X	X		X			X		X			X		X			
**Post-dose follow-up (days)**			2	7	2	7	28	2	7 28	2	7	28	2	7 28	2	7	21 14
**Vital signs**	X	X	X	X	X	X	X	X	X	X	X	X	X	X	X	X	X
**Local & systemic events/reactions**		X	X	X	X	X	X	X	X	X	X	X	X	X	X	X	X
**Diary cards provided**		1		2			3		4			5		6			
**Diary cards collected**				1		2			3		4			5		6	
**HLA typing (mL)**		3															
**HBV, HCV, HIV (mL)**	5																
**Biochemistry (mL)**	4			4		4	4		4		4	4		4		4	4
**Haematology (mL)**	2			2		2	2		2		2	2		2		2	2
**Antibody assays (mL)**		10					10					10					10
**Gene expression (mL)**		10												10			10
**Exploratory immunology (incl ELISPOT) (mL)**		50				50			50		50			50		50	50
**Blood volume (mL)**	**11**	**73**	**0**	**6**	**0**	**56**	**16**	**0**	**56**	**0**	**56**	**16**	**0**	**66**	**0**	**56**	**76**
**Cumulative blood volume (mL)**	**11**	**84**	**84**	**90**	**90**	**146**	**162**	**162**	**218**	**218**	**274**	**290**	**290**	**356**	**356**	**412**	**488**

* Timeline is approximate only. Exact timings of visits relate to the previous visit – *i.e.* each visit must fall the specified number of days after the last visit ± time window

† Timing of the subsequent malaria challenge is not precise, due to variability in parasite culture. The time window for this vaccination is therefore wider to ensure that this vaccination will precede the day of challenge by 21 ± 7 days

Grey columns highlight vaccination days

Number of visits : 17. Number of vaccinations : 6.

FLOW CHARTS: 2. CHALLENGE VISITS

Challenge attendance no.	1	2†	3	4	5 - 19	20 – 28	29	30
Trial timeline (days)*	D77	D77	D83.5	D84	D84.5 – D91	D92 – D100	D112	D167
Time window (days)	±7	±7					±10	±14
Attendance reference		C	C+6.5	C+7	C+7 – C+14.5	C+15 – C+23	C+35	C+90
Malaria challenge		X						
Physical examination								
β-HCG urine test		X						X
Review contraindications		X						
Vital signs	X		X	X	X	X	X	X
Local & systemic events/reactions	X		X	X	X	X	X	X
Thick smear / PCR (mL)		2	2	2	2 × 15	2 × 9		
Biochemistry (mL)	4						4	4
Haematology (mL)	2						2	2
Antibody assays (mL)	10							10
Gene expression (mL)	10			10				10
Exploratory immunology (incl ELISPOT) (mL)	50	50†		50			50	50
**Blood sampling (mL)**	**76**	**52**	**2**	**62**	**30**	**18**	**56**	**76**
**Cumulative blood taken – groups 1 & 2 (mL)**	**470**	**522**	**524**	**586**	**616**	**634**	**690**	**766**
**Cumulative blood taken – group 3 (mL)**	**488**	**540**	**542**	**604**	**634**	**652**	**708**	**784**

* Timeline is approximate only. Exact timings of visits relate to the previous visit – *i.e.* each visit must fall the specified number of days after the last visit ± time window

† Visit 2 will only be necessary if the mosquitos are not available on the date of visit 1, in which case it will be necessary to take the additional blood samples listed. See Section 3.1 for details.

Grey column highlights day of challenge (DOC)

Abbreviations and Symbols Used

**3D7**	Clone of NF54 strain of *Plasmodium falciparum*
**AE**	Adverse event
**ALT**	Alanine aminotransferase
**ALP**	Alkaline phosphatase
**AMA-1**	Apical membrane antigen-1
**CCVTM**	Clinical Centre for Vaccinology and Tropical Medicine
**CMI**	Cell-mediated immunity
**CRF**	Case report form
**CSP**	Circumsporozoite (**CS**) protein
**CTL**	Cytotoxic T lymphocytes
**ELISA**	Enzyme-linked immunosorbant assay
**ELISPOT**	Enzyme-linked immunospot
**FBC**	Full blood count
**FDA**	Food and Drug Administration
**FFM ME-TRAP**	The sequence of vaccines FP9, FP9, MVA all containing the ME-TRAP insert
**FP9**	Fowlpox 9
**FP9 ME-TRAP**	Fowlpox 9 vaccine containing ME-TRAP insert
**GCP**	Good Clinical Practice
**GMP**	Good Manufacturing Practice
**GP**	General Practitioner (Family Doctor)
**HA**	Hemagglutinin influenza glycoprotein
**IDT**	Impfstoffwerk Dessau-Tornau GmbH
**IEC**	Independent Ethics Committee
**IFN-γ**	Gamma interferon
**IRB**	Independent Review Board
**HBV**	Hepatitis B virus
**HCV**	Hepatitis C virus
**HIV**	Human immunodeficiency virus
**HLA**	Human leukocyte antigen
**LFT**	Liver function test
**LSM**	Local safety monitor
**ME-TRAP**	Multi-Epitope string and Thrombospondin Related Adhesion Protein
**MHRA**	Medicines and Healthcare products Regulatory Agency
**MVA**	Modified vaccinia Virus Ankara
**MVA ME-TRAP**	Modified vaccinia Virus Ankara vaccine containing the ME-TRAP insert
**NA**	Neuraminidase influenza glycoprotein
**NANP**	Asparagine-alanine-asparagine-proline
**NHS**	National Health Service
**NYVAC**	New York Vaccinia - a highly attenuated vaccinia virus
**OxREC**	Oxford Research Ethics Committee
**PBMC**	Peripheral blood mononuclear cells
**PCR**	Polymerase chain reaction
**PEV3A**	Virosomal vaccine containing mixture of PEV301 and PEV302
**PEV301**	Virosomal vaccine containing synthetic peptides from AMA-1
**PEV302**	Virosomal vaccine containing synthetic peptides from CSP
**SAE**	Serious adverse event

**SoAE**	Solicited Adverse event
**SOP**	Standard Operating Procedures
**TRAP**	Thrombospondin Related Adhesion Protein

1 INTRODUCTION

1.1 Background

In most tropical and many subtropical developing countries, malaria is a major cause of morbidity, especially among young children and pregnant women, and mortality, principally among infants and toddlers. In Africa, a child dies from malaria infection every 30 seconds [1]. Malaria also represents a serious health hazard for travellers who visit malaria-endemic areas. Worldwide, it is estimated that there are 500 million infections [2] and 1 million deaths caused by falciparum malaria each year. The widespread resistance of Anopheles mosquitoes to insecticides has crippled national and regional malaria control programs based on vector control interventions. Furthermore, the increasing prevalence of chloroquine and anti-folate resistant strains of *P. falciparum* in Asia, Africa and South America has created a crisis in the clinical treatment of malaria in many countries. These drug-resistant strains have made devising well-tolerated and effective prophylactic regimens challenging. Given these facts, there is an urgent need for new, effective interventions that can be applied to the control of malaria. One such approach involves immunoprophylaxis by means of malaria vaccines. The novel combination of the candidate vaccines to be studied could be useful in protecting residents of malaria-endemic countries against malaria infection and disease. Prime boost regimes using recombinant Modified vaccinia Virus Ankara (MVA) and Fowlpox 9 (FP9) with Multi-Epitope and Thrombospondin Related Adhesion Protein (ME-TRAP) appear safe, produce high levels of malaria specific T lymphocytes and demonstrate some protection against malaria [3-5]. PEV3A is a new virosomal vaccine combining two *P. falciparum* antigens recently tested in a Phase I clinical trial (Pevion Biotech Ltd., unpublished data). The results showed excellent tolerability and induction of strong cell-mediated and humoral responses. By administering these vaccines alone and in combination we hope to show protection against malaria infection that is enhanced when both vaccines are used together.

1.1.1 Lifecycle of the Malaria Parasite

Malaria parasites have a complex life cycle. Infected mosquitoes transmit the malaria sporozoites in their saliva to humans while taking a blood meal. The sporozoites travel through the blood stream and invade hepatocytes where they mature into merozoites. After 6 to 10 days, the infected hepatocytes rupture, releasing a large number of merozoites into the bloodstream. These merozoites then invade erythrocytes where they multiply and, after two days, release progeny merozoites that invade new erythrocytes to perpetuate the erythrocytic cycle. This cycle is responsible for clinical illness in man. A small percentage of the merozoites do not multiply after invading erythrocytes, but instead differentiate into sexual forms (gametocytes). When ingested by a mosquito, male and female sexual

forms can unite, creating a zygote. The zygote matures and releases sporozoites, which migrate to the mosquito's salivary glands, thus completing the lifecycle.

1.1.2 T Lymphocytes and protection against liver-stage malaria

There are several lines of evidence that T lymphocytes are involved in protection against malaria. Immunisation of mice with irradiated sporozoites induces protection against malaria challenge [6]. This protective immunity to *Plasmodium berghei* sporozoite challenge can be transferred to other mice by transferring the T lymphocyte clones that were induced by irradiated sporozoites. T lymphocyte recognition of circumsporozoite protein-derived peptide on infected hepatocytes led to lysis of the infected cell and parasite death [7]. T cell immunity against blood-stage antigens can induce protection against *P. yoelii* murine malaria [8]. In humans there are several pieces of indirect evidence for a T lymphocyte role in protection. Firstly, human leukocyte antigen (HLA) class-I restricted T lymphocyte responses have been demonstrated in both residents of endemic areas repeatedly exposed to *P. falciparum* and in malaria-naïve volunteers protected form sporozoite challenge by irradiated sporozoite immunization [9-11]. T cells specific to *P. falciparum* epitopes were shown to be present in low levels in adult Gambians [12, 13], raising the possibility that boosting these by immunization may be beneficial. Secondly, HLA-B*53, which restricts the immune response of CD8+ T lymphocytes, and HLA-DRB1*1302 which restricts the immune response of CD4+ T lymphocytes, were associated with resistance to severe malaria in a large case-control study of Gambian children [14]. Cytotoxic T lymphocytes to a conserved *P. falciparum* epitope restricted by HLA-B*53 have been identified and these derive from the LSA1 and LSA3 antigens [10, 15]. Thirdly, some *P. falciparum* T lymphocyte epitopes are in highly polymorphic regions in which all nucleotide substitutions encode amino acid changes. The observation that no synonymous changes occur argues strongly that these regions are subject to selection pressure from T lymphocytes. This is supported by the identification of altered peptide ligand antagonism of T lymphocytes as an immune escape mechanism [16].

1.1.3 Development of a T lymphocyte inducing vaccine against *P. falciparum*

Following the discovery that in some Gambians HLA-B*53 restricted T cells recognise a conserved nonamer peptide from liver-stage antigen-1, a peptide-based approach using allele-specific motifs was applied to several other class I HLA molecules. In total fourteen class I epitopes were identified in six pre-erythrocytic *P. falciparum* antigens [9]. In a series of immunisation studies in mice various potential vaccine types (recombinant particles, peptides, plasmid DNA, numerous adjuvants and recombinant vectors such as recombinant BCG, Salmonella, adenovirus, MVA and FP9), encoding malaria epitopes and antigens, were compared [17, 18]. Most of these approaches induced only modest levels of T cell immunogenicity that invariably failed to protect against sporozoite challenge. However, a priming immunisation with plasmid DNA encoding an entire *P. berghei* pre-erythrocytic antigen followed by a booster immunisation with a MVA vector carrying the same antigen induced complete protection [18] in strains of mice highly susceptible to sporozoite challenge [19]. Protection was associated with very high frequencies of splenic peptide-specific interferon-γ (IFN-γ) secreting CD8+ T cells, and the level of these cells measured in IFN-γ enzyme-linked immunospot (ELISPOT) assays correlated well with protection. The US Navy group have reported similar results for *P. yoelii* malaria using NYVAC (a highly attenuated vaccinia virus, New York Vaccinia) as the boosting vector [20]. Furthermore in chimpanzee studies, priming with DNA and boosting with MVA has yielded very high levels of T cells

to a *P. falciparum* epitope [21]. This vaccination approach, in which heterologous immunisations are given with two carriers encoding the same antigen, epitope or combination of antigens and minimal epitopes, has become known as prime-boost. Since this technique has induced several fold higher levels of antigen-specific T cells than previous subunit vaccines, the hope is that effective vaccination against diseases depending on cellular responses for protection may be achievable. DNA vaccines can prime but are not good boosting agents. Recombinant viruses such as MVA, FP9 and non-replicating strains of adenovirus can probably both prime and boost (although two different carriers need to be used in each regime, heterologous recombinant viral prime-boost).

Studies by various groups have now found that prime-boost is highly immunogenic for CD4+ and CD8+ T cell induction against, tuberculosis [22, 23], HIV [24], and Ebola (DNA/adenovirus) [25]. Recently, DNA/MVA polyprotein vaccinations controlled a mucosal challenge of a highly pathogenic SIV/HIV chimera and prevented AIDS in a macaque model. In this study challenge occurred 7 months after final vaccination [26].

To assess the immunogenicity in clinical studies we use the gamma-interferon enzyme linked immunospot (ELISPOT) assay in two forms; the *ex vivo,* and the short-term culture form. The *ex vivo* form correlated directly with protection in two mouse models of malaria. The short-term cultured form correlated with protection in a field study of the vaccine candidate RTS,S/AS02A in the Gambia [27]. The *ex vivo* ELISPOT will be done on all samples using freshly isolated PBMC whereas the short-term cultured ELISPOT will be done retrospectively using PBMC that have been stored frozen, on selected samples such as those from protected individuals.

1.1.4 Prime-boost clinical studies in Oxford

Since August 1999 a series of Phase I clinical studies in Oxford have assessed the prime-boost immunisation approach in healthy, malaria-naïve human volunteers. These studies, using the vaccines DNA-ME TRAP, MVA ME-TRAP, FP9-ME TRAP and MVA-CSO, DNA-CSO and FP9-CSO have demonstrated safety and immunogenicity, with significant *ex vivo* ELISPOT responses [3, 28, 29]. Following immunisation most subjects then participate in a subsequent study in Oxford where they are challenged with the 3D7 strain of *P. falciparum* under close observation to assess vaccine efficacy. Recent studies have demonstrated promising results [30], including complete protection against heterologous malaria challenge in two out of five vaccinated subjects who had received two doses of FP9 ME-TRAP followed by one dose of MVA ME-TRAP [4] and one subject who received two doses of DNA ME-TRAP and one dose of MVA ME-TRAP. Furthermore, several immunisation regimes using the ME-TRAP insert have led to highly statistically significant delays in time to patient parasitaemia. Analysis of this delay shows that it corresponds to a vaccine-induced, greater than 80% reduction in liver stage parasite number on day 6.5 post-sporozoite challenge [5].

1.2 Vaccine vectors

1.2.1 Modified Vaccinia virus Ankara (MVA) vector

MVA is an attractive candidate orthopox vaccine vector for safety and immunogenicity reasons. The successful worldwide eradication of smallpox via vaccination with live vaccinia virus highlighted

vaccinia as a candidate carrier. Although millions of humans have been vaccinated with conventional live vaccinia virus, its small but definite risk to both researchers and future patients led to the development of several attenuated strains of vaccinia during smallpox eradication and more recently. In particular the host-range restricted MVA proved to be extremely attenuated compared to other vaccinia viruses.

MVA was originally derived from the vaccinia strain Ankara by over 500 serial passages in primary chicken embryo fibroblasts. MVA has six major genomic deletions compared to the parental Ankara genome and is severely compromised in its ability to replicate in mammalian cells. No replication has been documented in non-transformed mammalian cells. The viral genome has been proven to be stable through a large series of passages in chicken embryo fibroblasts. Using restriction enzyme analysis virtually no difference was observed between passage 500 – 572 [31]. MVA also showed no cytopathic effect or plaque formation in cells of human origin. In irradiated mice MVA did not elicit any morbidity or lethality even when administered at high doses intra-cerebrally, indicating its safety even in immuno-compromised organisms [31]. Apart from studies in mice, rabbits and elephants [32] MVA has been shown to be safe in humans. From 1972 until 1980 (the end of compulsory smallpox vaccination) MVA was licensed in Germany [32] and was included in the official immunisation schedule [33]. In a large field study carried out in Germany in the late seventies over 120,000 previously unvaccinated individuals were vaccinated with MVA (0.2 mL) administered either intra-dermally or subcutaneously. The study population included high-risk groups (*e.g.* people suffering from allergies, elderly people, alcoholics). Given intra-dermally, a red nodule of up to 4 mm in diameter was observed at the injection site at day 4 or 5. Only a small proportion showed any systemic side effects such as fever over 38.5°C [31]. MVA proved to be non-contagious and avirulent. Viral replication is blocked late during infection of cells but importantly viral and recombinant protein synthesis is unimpaired even during this abortive infection. Replication-deficient recombinant MVA has been viewed as an exceptionally safe viral vector. When tested in animal model studies recombinant MVAs have been shown to be avirulent, yet protectively immunogenic as vaccines against viral diseases and cancer [31]. Recent studies in macaques severely immuno-suppressed by SIV infection have further supported the view that MVA should be safe in immuno-compromised humans [34].

All MVA constructs are made by ligating the selected sequence into the vaccinia shuttle vector pSC11, placing it under the control of the vaccinia P7.5 early/late promoter. This vector also encodes a copy of the β-galactosidase gene to allow plaque picking [35]. The MVA products are manufactured under Good Manufacturing Practice (GMP) conditions by the contract manufacturer IDT (Rosslau, Germany).

1.2.2 Fowlpox 9 (FP9) vector

Fowlpox virus is a member of the avian poxvirus family and is an obligate pathogen of chickens causing cutaneous or mucosal symptoms. FP9 is a highly attenuated form of an avian pox virus derived by 438 serial passages of the wild-type fowlpox virus HP-1 [36]. It was originally developed and used as a vaccine for poultry, but its ability to infect mammalian cells and produce proteins without replicating

[37] led to interest in its use as a recombinant viral vector for mammalian, including human, vaccination. FP9 was shown to be an effective vector, delivering antigen to human dendritic cells and stimulating a class-I restricted T-cell response both *in vitro* and *in vivo* [38]. The FP9 genome has now been sequenced and found to have 118 mutations when compared to the pathogenic US fowlpox strain [39].

FP9 recombinants are constructed using an established protocol [40]. The DNA sequence of interest is ligated into the *Sma*I cloning site of the Fowlpox shuttle vector pEFL29 [40] placing expression of this gene under the control of the vaccinia virus P7.5 promoter. The pEFL29 plasmid also encodes a copy of the β-galactosidase gene under the control of the FP4b fowlpox late promoter, allowing identification of recombinant viruses by X-gal staining as described previously for vaccinia virus [35]. Recombinant viruses are prepared by *in vitro* recombination of the shuttle vector with the FP9 fowlpox strain in primary cultures of chick embryo fibroblasts (CEF). Recombinant viruses are repeatedly plaque purified in CEF monolayers until homogenous. A stock of FP9 containing the insert of interest is then supplied to IDT (Rosslau, Germany) for production of the clinical lot under GMP conditions.

1.2.3 Virosomal antigen carrier system

PEV3A is a virosomal vaccine containing two peptide antigens from the pre-erythrocytic and blood stages of the malaria parasite. Virosomes are essentially reconstituted but 'empty' influenza particles – spherical liposomal structures that do not contain any nuclear material, but do display two influenza surface antigens, neuraminidase (NA) and hemagglutinin (HA). They are formed by treating influenza virus with detergent to cause dissociation of the glycoproteins and phospholipid structure. The NA and HA glycoproteins are purified, nucleocapsid removed and phospholipid added, resulting in the spontaneous formation of the spherical virosomal structures [41]. Other peptides can then be attached to the particles, anchored in the lipid membrane or incorporated into the structure itself.

The NA and HA surface glycoproteins enable virosomes to bind and be internalised by antigen presenting cells, delivering potentially immunogenic peptides for processing in the MHC system. Peptides bound to the virosomal surface are degraded within endosomes for MHC II presentation whereas those incorporated within the virosome are delivered into the cytoplasm for processing via the MHC I system [42]. In mice a specific CTL response to intramuscular virosome-encapsulated peptide can be demonstrated that is not seen when peptide is used alone or simply mixed with virosomes, or carried in fusion-inactivated virosomes. Thus virosomes, through their surface fusion glycoproteins, are capable of stimulating both a humoral and cell-mediated immune response, which is likely to be beneficial in the development of an effective malaria vaccine.

There is also evidence that virosomes can act as potent adjuvants to stimulate high titres of antibodies even when antigen is simply mixed with the virosomal preparation and of a long-lasting 'depot-effect' when used to deliver streptavidin [43]. There is, therefore, the potential to achieve liver and blood-stage protection with this approach.

The first virosomal vaccine was licensed in 1996 (the hepatitis A vaccine *Epaxal*®). It demonstrated excellent safety and tolerability [44] and subsequently the influenza vaccine *Inflexal*® *V* has entered

clinical use. Together these vaccines have been licensed in over 29 countries and administered to over ten million adults and children to date. As they do not contain nuclear material, virosomal vectors are unable to replicate and cannot transform to become replication competent.

1.3 Summary of previous clinical trials

1.3.1 MVA vaccines

Recent studies in healthy volunteers in Oxford and The Gambia have used MVA with two inserts, 'ME-TRAP' and 'CS'. MVA ME-TRAP contains a fusion protein of multiple epitopes (ME) and the *P. falciparum* pre-erythrocytic thrombospondin-related adhesion protein (TRAP). The 'ME' is a string of 20 epitopes fused to the thrombospondin-related adhesion protein, described in [3]. TRAP was selected as it is well characterized and has a protective homologue in rodents [19]. The ME string is fused to the entire sequence of the T9/96 strain of *P. falciparum* TRAP and the ME-TRAP hybrid is a 2398 base-pair insert which encodes for a single polypeptide of 789 amino acids [28, 30].
To date MVA ME-TRAP has been administered to over 350 healthy volunteers in Oxford [3] and The Gambia [45] without any serious adverse events. Volunteers have received one to three doses of from 3 to 15 $\times$ 10^7 pfu per dose of intra-dermal vaccine at three to four-week intervals. All subjects have temporary local redness with typically a 5 – 10 mm central red area with a paler pink surrounding area, difficult to discern on dark skin, that ranges in size from about 1 - 7 cm in diameter and peaks at 48 hours post vaccination. At seven days post vaccination generally only the central red area remains and is usually (in more than 50% of cases) absent or almost indiscernible at one month post vaccination. At four to six months post-vaccination there may be some very minor discolouration or freckling at the vaccination site. Some temporary superficial scaling of the skin generally occurs. Most subjects have local discomfort and tenderness at the vaccination site. The local reactions have been mildly painful in most subjects with moderate local pain in a minority. At higher doses (1.5 $\times$ 10^8 pfu) about two thirds of subjects have had a mild flu-like illness (symptoms of malaise, myalgia and feverishness) after the first dose of MVA ME-TRAP that was short-lived (12 to 24 hours). This occurs within 24 hours of vaccination and was rarer (following fewer than 15% immunisations) with lower doses and is much less frequent with second or third MVA immunisations.
Full dose MVA ME-TRAP (1.5 $\times$ 10^8 pfu) has been administered intra-dermally to over 150 adult Gambian males in a phase IIb study with acceptable short term reactogenicity. More detailed analysis of a phase I study with the same dose of MVA ME-TRAP administered to 29 adult Gambians in early 2002 suggests a somewhat better reactogenicity profile than in UK volunteers [46]. There have been no serious adverse events with the MVA ME-TRAP vaccine.
The other MVA malaria vaccine used in trials in Oxford, MVA CS expresses the entire circumsporozoite protein from the 3D7 strain of *P. falciparum*. MVA CS has been administered to 71 healthy volunteers in Oxford. Over all, it has been well tolerated without causing any serious or severe side effects. There were no significant abnormalities in biochemical or haematological parameters.

Preliminary data suggest MVA CS has a side-effect profile similar to MVA ME-TRAP as described above.
The emerging safety profile of recombinant MVA vaccine is supported by data from clinical studies of three other MVA recombinants made in Oxford and currently in clinical studies. Andrew McMichael's group has studied a MVA for HIV in over 40 individuals in the UK and Kenya, and MVA recombinants for Hepatitis B virus (HBV) and melanoma have been studied in the UK and the Gambia (HBV only) by Oxford University. We understand that there have been no serious vaccine-related adverse events in these studies to date.

1.3.2 FP9 vaccines

Two different FP9 vaccines have been used so far in clinical trials in Oxford, FP9 ME-TRAP and FP9 CS. To date 87 doses of FP9 ME-TRAP have been given to 55 volunteers in Oxford [3] using various regimens in combination with DNA as well as recombinant MVA vaccines. The local and systemic safety profile of FP9 ME-TRAP is comparable to that described for recombinant MVA vaccines. No vaccine related Serious Adverse Event (SAE) has been observed. Pain at the injection site and erythema are the predominant local side effects (Table 1~~Table 1~~ and Table 2~~Table 2~~) with the erythema being maximal within 2 to 3 days post-vaccination before receding.

Vaccine regime (number of subjects)	Pain score				Duration of pain (mean and range in days)
	0	1	2	3	
After DNA/ vaccine naïve (29)	4	18	6	1*	4.03 (1-19)
After MVA (9)	0	5	3	1*	3.56 (3-5)
After FP9 (21)	4	14	3	0	2.10 (0-5)

Table 1: Pain following FP9 ME-TRAP vaccination given
* pain lasted for less than 24 hours and became unnoticeable in 4 to 6 days

	Vaccine regime			
	FF	FFM	DDMF or DDFM	FM or MF
Diameter in mm (range)	70 (25-70)	90 (15-90)	50 (5-50)	70 (0-70)

Table 2: Maximal erythema observed after FP9 used in different vaccination regimen
Regimes are described by sequence of vaccination: F = FP9; M = MVA; D = DNA vaccine.

The commonest systemic side effect after FP9 ME-TRAP is of feeling feverish although this is often not associated with a documented fever. Other solicited side effects were myalgia, arthralgia, headache and nausea. FP9 CS has been used recently in a phase I study in 25 healthy volunteers in Oxford, at doses of 1×10^8 pfu. The construct expresses the entire codon optimised CS protein for the 3D7 strain of *P. falciparum*. Analysis of safety and tolerability suggests a similar side effect profile to FP9 ME-TRAP. No serious adverse events were noted in the study.

In general, it appears that the frequency of systemic side effects in response to MVA and FP9 vaccines is affected by a preceding poxvirus vaccination with the proportion of subjects experiencing any systemic side effects after the first vaccination being 69%, decreasing to 37% after the second and 22% after a third immunisation [3].

A regimen using 1×10^8 pfu of FP9 ME-TRAP given twice 3 weeks apart, boosted by 1.5×10^8 pfu of MVA ME-TRAP induced a good response in IFN-γ ELISPOT when TRAP was used as an antigen and resulted in a significant delay in parasitaemia after sporozoite challenge. In one of the studies using this regimen 2 of 5 volunteers were protected completely [4]. Analysis of a larger group of 19 vaccinees showed on average a 92% reduction in liver-stage parasite numbers in vaccines receiving this regime [4, 5].

1.3.3 Virosomal vaccines

The two malaria antigens that are incorporated in PEV3A are derived from the circumsporozoite (CS) and apical membrane antigen-1 (AMA-1) proteins of *P. falciparum*. This employs novel technology to generate conformational epitopes by using cyclized peptides. This approach overcomes one of the limitations of linear peptides and allows the formation of more immunogenic epitopes. In particular the AMA-1 peptide [47] has been shown to be immunogenic, inducing in mice antibodies that show growth inhibitory activity. AMA-1 is a blood-stage surface antigen present on merozoites that appears to be involved with their invasion of red cells. It can induce immunity to malaria in both mouse and monkey models [48-50]. It's amino acid sequence is as follows:
GGCYKDEIKKEIERESKRIKLNDNDDEGNKKIIAPRIFISDDKDSLKCG [47].

The NANP peptide from the CS antigen is the major B cell epitope known to be presented on malaria sporozoites. Large numbers of anti-sporozoite vaccine candidates have been designed attempting to induce antibodies against this sequence. The most successful of these candidates, RTS,S/AS02 [51], has now completed a phase IIb trial in African children and showed significant protection [52]. This vaccine candidate induces high levels of antibodies against the NANP B cell epitope and there is evidence that these antibodies may contribute to the observed protective efficacy. The PEV3A vaccine displays this epitope in an immunogenic form that leads to induction of antibodies that recognize sporozoites. The sequence of this immunogenic peptide is: NPNANAproNANPNANPNENPNA [53]. A Phase I trial examining safety and immunogenicity of both antigens as virosomal vaccines was recently conducted in Switzerland, involving 40 volunteers receiving 3 vaccinations each and 6 control subjects (Pevion Biotech Ltd., unpublished data). The results were encouraging, with only 6 vaccine related mild adverse events, confirming good safety and tolerability at all doses and high titres of *P. falciparum*-specific antibodies, with a good response when both antigens were given together.

2 Trial Objectives

2.1 Primary Objective

To assess protection against *P. falciparum* malaria infection following the virosomal vaccine PEV3A alone or in combination with FP9-MVA ME-TRAP vaccination.

2.2 Secondary Objectives

To confirm safety and immunogenicity of PEV3A vaccination alone or in combination with FP9-MVA ME-TRAP vaccination.

2.3 Tertiary Objectives

To assess long term efficacy of PEV3A vaccination alone or in combination with FP9-MVA ME-TRAP in a late malaria challenge of volunteers protected at initial challenge.

3 TRIAL DESIGN AND METHODOLOGY

3.1 Trial design

3.1.1 Description and Justification of the Trial Design

This is a Phase I/IIa study in healthy malaria naïve adult subjects aged 18 – 50 years. There will be three study groups, receiving either PEV3A alone, both PEV3A and FFM ME-TRAP administered together, or both vaccinations separated by one week. 36 volunteers will be recruited and, where volunteers do not request allocation to a particular group, randomised into the three groups. There will also be a control group of six volunteers recruited for each challenge phase alone who will not receive any vaccinations. This group is necessary to demonstrate the infective efficacy of the challenge system and to provide a comparison for the vaccinated groups.

The dose of PEV3A will be 0.5 mL (containing 50 μg of PEV301 and 10 μg of PEV302), given as an intramuscular injection in the deltoid region of the upper arm. The doses of FP9 and MVA vaccines will be 1×10^8 pfu and 1.5×10^8 pfu respectively, and these will be administered intradermally into the skin overlying the deltoid.

All volunteers will take part in a sporozoite challenge 3 weeks after receiving the final PEV3A vaccination. All volunteers protected at first challenge will then be invited to take part in a further late challenge at 6 months after their last challenge.

The challenge will occur at the insectary at Imperial College, London. All the volunteers will live in or near Oxford such that they are able to attend the required frequent visits to The Clinical Centre for Vaccinology and Tropical Medicine (CCVTM), Oxford.

3.1.2 Randomisation and Group Allocation

Volunteers for the vaccination phase will be randomised to groups 1, 2 or 3 except where an individual requests allocation to a particular group. To achieve equal numbers of volunteers in each group, restricted randomisation will be performed using random number tables as previously described [54]. Volunteers to act as controls will be recruited after the main study group, and so will not be randomised.

Each participant will be allocated a unique study number when they are invited for screening. These are consecutive numbers that follow on from those used in previous malaria vaccine trials in Oxford. See Appendix C.7 for further details.

3.2 Trial Plan

3.2.1 Trial Calendar/Timelines

Volunteers will be enrolled in this study (including two month screening period, vaccinations and challenge) for up to 18 months. The initial trial period, up to the end of the first challenge, will be 8 months. Those volunteers who are protected from malaria will be invited to participate in a second challenge, approximately 6 months after the first, and so they will be enrolled for a possible maximum of 18 months.

Depending on the MHRA and COREC approval date, the 60 day screening period is expected to commence in May 2005, with vaccinations beginning in July.

3.2.2 Vaccination and Serology schedule

In all groups detailed below there is an expanded window for administering the third vaccination (or pair of vaccinations for groups 2 & 3). This is based on the unpredictability of the parasite culture used in the challenge. Since it is desirable to maintain a fixed interval from last vaccination to time of challenge, this window of time for last vaccination can accommodate the challenge protocol.

3.2.2.1 Group 1 ("PPP")

Up to 12 subjects will receive a single dose of PEV3A (P) on three occasions at the following times: baseline, 4 weeks (± 1 week) later and a further 4 weeks (+4/- 2 weeks) later.

Blood samples (BS) will be collected at screening and then on day 0, day 7, day 28, day 35, day 56, day 63 and day 77 (see page xiv for details).

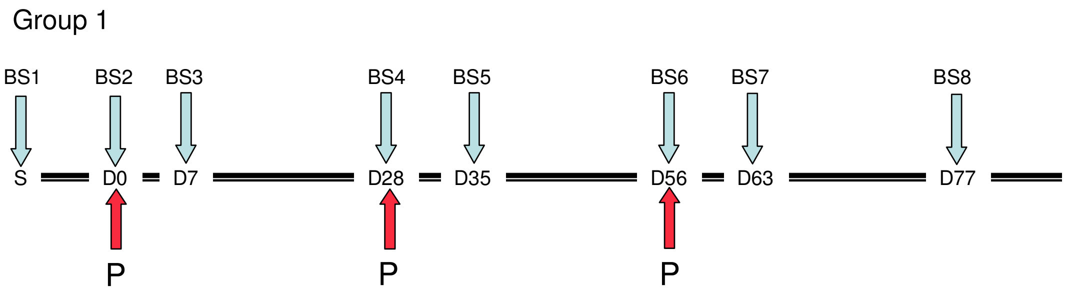

3.2.2.2 Group 2 ("PFPFPM Coadmin")

Up to 12 subjects will receive doses of vaccines at the following times: PEV3A and FP9 ME-TRAP at baseline; PEV3A (P) and FP9 ME-TRAP (F) at 4 weeks (± 7 days) later; and PEV3A and MVA ME-TRAP (M) at 4 weeks (+4/- 2 weeks) later.
Blood samples (BS) will be collected at screening and then on day 0, day 7, day 28, day 35, day 56, day 63 and day 77 (see page xiv for details).

Group 2

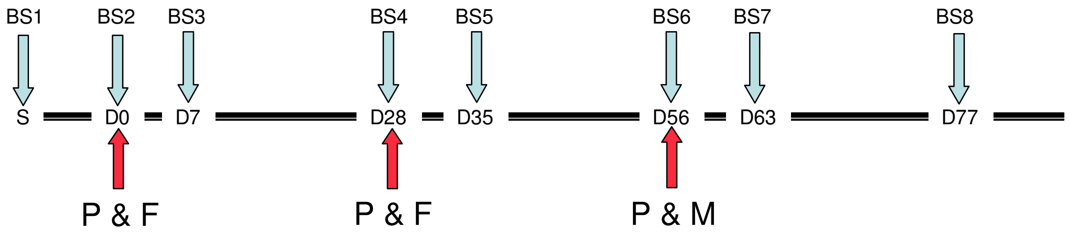

3.2.2.3 Group 3 ("PFPFPM Staggered")

Up to 12 subjects will receive doses of vaccines at the following times: PEV3A at baseline; FP9 ME-TRAP at 1 week (± 3 days) later; PEV3A at 3 weeks (± 7 days) later; FP9 ME-TRAP (F) at 1 week (±3 days) later; PEV3A at 3 weeks (+4/- 2 weeks) later; and MVA ME-TRAP (M) 1 week (±3 days) later.
Blood samples (BS) will be collected at screening and then on day 0, day 7, day 14, day 28, day 35, day 42, day 56, day 63, day 70 and day 77 (see page xv for details).

Group 3

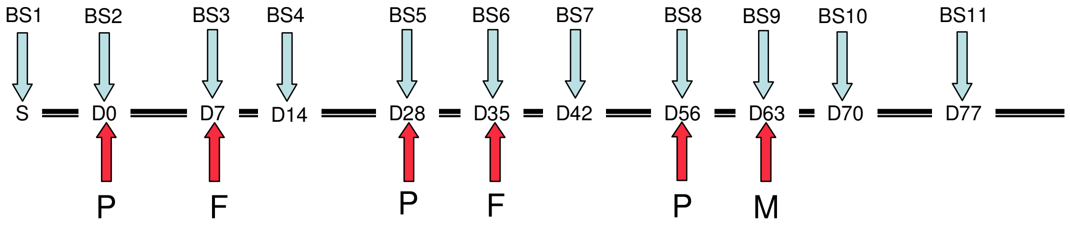

3.2.2.4 Challenge

After the vaccinations, all groups will be challenged (on day 77). To allow for variability of sporozoite culture, we have included an extra visit for the day of challenge. If this occurs on or near day 77 (± 7 days), only one set of blood samples will be taken. After the day of challenge (DOC), blood samples will be taken on day 83, twice daily from day 84 to day 91, once daily from day 92 to day 100 and then on day 112 and day 167 (see page xvi for details).

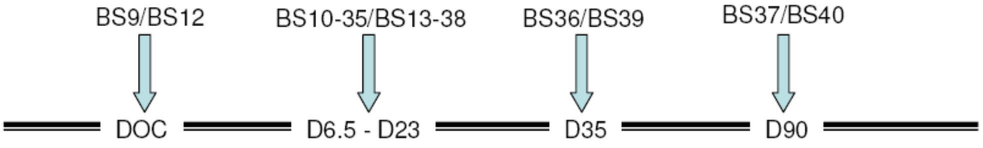

D= Days post challenge (D6.5 = day 84, D23 = day 91, D35 = day 112, D90 = day 167)

3.2.2.5 Late Challenge

All volunteers who are protected from malaria at the first challenge will be invited to participate in a second, late challenge. This will take place approximately 6 months (4-12 months) after the first challenge. Volunteers willing to participate will be asked to attend a 're-screening visit,' where eligibility will be reassessed and safety bloods will be taken. Volunteers will not be excluded if they have travelled to a malaria endemic area since the first challenge, as long it is not within 3 months of the late challenge, and any chemoprophylaxis they may have taken during that time satisfies the relevant exclusion criterion. They will also be asked to sign another consent form to indicate their willingness to take part. This challenge will be conducted according to the same procedure as the first (described in Sections 6 & 7 of this protocol), and the visits will follow the same SOPs outlined in Appendix C. Blood samples will be taken as outlined in flow diagram above. The last visit of this part of the trial will be 35 days after the day of challenge.

3.2.3 Trial Centres

Clinical work and research based laboratory investigations:
Centre for Clinical Vaccinology and Tropical Medicine (CCVTM), Old Road, Headington, Oxford, OX3 7LJ. Adjacent to the wards of the Churchill Hospital.

Malaria sporozoite challenge:
Imperial College, London

Routine laboratory investigations:
The John Radcliffe Hospital, Headley Way, Headington, Oxford, OX3 9DU.
The Churchill Hospital, Old Road, Headington, Oxford, OX3 7LJ.

Both hospitals are part of The Oxford Radcliffe Hospitals NHS Trust and constitute one centre, but are situated approximately half a mile apart, in Headington, Oxford.

3.2.4 Conduct of the Trial

3.2.4.1 Interruption of the Trial

The trial may be discontinued for administrative reasons if new data about the investigational products resulting from this or any other trials become available, or on advice of the sponsor, the investigators, an Independent Review Board (IRB) or Ethics Committee.
If a trial is prematurely terminated or suspended, the investigators, the Regulatory Authorities and the IRBs/Ethics Committee(s) will be promptly informed of the reason for termination or suspension as specified by the applicable regulatory requirements.

3.2.4.2 Safety Monitoring

The study will have an appointed Local Safety Monitor (LSM) who is an on-site Consultant with a special interest in infectious diseases at the Oxford Radcliffe NHS Trust but is independent from the study and can review events in 'real time'. All severe (grade 3) and serious adverse events (as defined in section 8.1.4.) will be reported to him within 24 hours of the investigators' knowledge of the event. The LSM will be empowered to review and evaluate the safety data as needed and make recommendations to the investigators concerning the continuation, modification or termination of the study.
Findings of a serious and immediate nature including any recommendations to modify or discontinue all or part of the study must be reported immediately to the LSM in verbal and written form.

The LSM may suspend the study if:

- One or more volunteers has a serious adverse event related to the challenge
- The LSM believes there are any safety problems with the challenge or follow up that may endanger the volunteers

The LSM may terminate the study if:

- There is a pattern of serious or severe adverse events that may endanger the health and welfare of the volunteers. The LSM will review the pattern, severity, grading, timing and causality of the adverse events in making the decision to terminate the study.
  Where subjects or the investigators discontinue the study, a follow-up visit for safety will be arranged

The LSM's role will also include:

- withdrawal of individual subjects from the study if deemed necessary because of any severe or serious adverse event
- provision of clinical advice on any illness in study subjects, especially in circumstances in which treatment might influence the course of the study

3.3 Case Report Forms and Data Collection

3.3.1 Source Data

All protocol required information will be directly entered into a StudyBuilder™ database designed by the Investigators. Data to be collected will detail every medical interaction in the clinical study including medical history and screening evaluation, records of physical examinations, vaccination details, concomitant medications, interim medical history taken at each visit and any solicited or unsolicited adverse events. Source documents such as laboratory results sheets and diary cards will be stored in paper files, in a locked filing cabinet.

3.3.2 Diary

Volunteers will receive a diary card after each vaccination, distributed on the day of vaccination, along with instructions on how to use it. These will be collected on day 7 following each vaccination. Groups 1 and 2 will therefore receive three diary cards and group 3 will receive six. The diary cards represent source documents and will therefore be stored in paper files in a locked filing cabinet.

3.3.3 Case Report Forms

The Investigator has generated case report forms (CRFs) which will contain source data. These are in electronic format, forming part of a StudyBuilder™ database. Data may only be entered into these electronic forms by one of the investigators or clinical research nurse. Access will be restricted by use of usernames and passwords. If a wrong entry is made into the forms, then these can be changed immediately or at a later date. The database allows for this, and stores the original entry, any subsequent changes and the date and time of those changes. The identity of the person making the changes will be recorded as their username. The names, positions, signatures and initials of these authorized staff members are documented and will be filed in a signature log in the regulatory binder.

3.4 Procedures for Obtaining, Handling and Shipment of Biological Samples

3.4.1 Obtaining Serum Samples

All blood samples will be drawn in the clinic rooms attached to the CCVTM. They will be taken by the investigators or clinical research nurse. The volumes of blood to be drawn at each visit are specified in the Flow Chart on pages xiv - xvi. All samples will be labelled with the subject's unique study number only, ensuring anonymity.
Blood for routine safety analysis will be transported to the laboratory at the John Radcliffe Hospital following the hospital procedures.

3.4.2 Handling Serum Samples

3.4.2.1 Aliquoting and Storage Conditions

Aliquoting will be performed subject by subject to avoid mixing blood tubes. The laboratory standard operating procedure will be followed.

Each aliquot must be frozen and stored at -20°C to -80°C.

4 Selection and Withdrawal of Subjects

4.1 Inclusion Criteria

Healthy adults aged 18 to 50 years
Written informed consent
Resident in or near Oxford for the duration of the vaccination study
For women only, willingness to practice continuous effective contraception during the study and (if participating) during the subsequent challenge study.
Agreement to refrain from blood donation during the course of the study
Willingness to undergo an HIV test

4.2 Exclusion Criteria

Any deviation from the normal range in biochemistry or haematology blood tests or in urine analysis as defined in Appendix A.
Prior receipt of an investigational malaria vaccine
Use of any investigational or non-registered drug, vaccine or medical device other than the study vaccine within 30 days preceding dosing of study vaccine, or planned use during the study period
Administration of chronic (defined as more than 14 days) immunosuppressive drugs or other immune modifying drugs within six months of vaccination. (For corticosteroids, this will mean prednisolone, or equivalent, ≥ 0.5 mg/kg/day. Inhaled and topical steroids are allowed.)
History of malaria chemoprophylaxis with chloroquine within 5 months prior to the planned challenge, with Lariam within 6 weeks prior to the challenge, and Riamet® within 2 weeks prior to the challenge
Any history of malaria
Travel to a malaria endemic area within the previous 6 months
Planned travel to malarious areas during the study period
Any confirmed or suspected immunosuppressive or immunodeficient condition, including human immunodeficiency virus (HIV) infection and asplenia
History of allergic disease or reactions likely to be exacerbated by any component of the vaccine, *e.g.* egg products
History of cancer (except basal cell carcinoma of the skin and cervical carcinoma in situ)
History of haemoglobinopathies: Sickle cell disease, thalassaemia, G6PD deficiency
History of diabetes mellitus
Chronic or active neurological disease including seizures
History of ≥ 2 hospitalisations for invasive bacterial infections (pneumonia, meningitis)
Suspected or known current alcohol abuse as defined by an alcohol intake of greater than 42 units every week
Seropositive for hepatitis B surface antigen (HBsAg) or hepatitis C virus (antibodies to HCV)
Hepatomegaly, right upper quadrant abdominal pain or tenderness
Evidence of serious psychiatric condition
Any on-going chronic illness requiring hospital specialist supervision
Administration of immunoglobulins and/or any blood products within the three months preceding the planned administration of the vaccine candidate
Pregnant or lactating woman
Any woman who is willing or intends to become pregnant during the study
Any history of anaphylaxis in reaction to vaccination
Principal Investigator assessment of lack of willingness to participate and comply with all requirements of the protocol

History or clinical evidence of intravenous drug abuse see Appendix C.6
Any other finding which in the opinion of the investigator would significantly increase the risk of having an adverse outcome from participating in this protocol

4.3 Re-vaccination exclusion criteria

The following adverse events associated with vaccine immunisation constitute absolute contraindications to further administration of vaccine. If any of these events occur during the study, the subject must be withdrawn and followed until resolution of the event, as with any adverse event.

- Anaphylactic reaction following administration of vaccine

Pregnancy

The following adverse events constitute contraindications to administration of vaccine at that point in time; if any one of these adverse events occurs at the time scheduled for vaccination, the subject may be vaccinated at a later date, or withdrawn at the discretion of the investigator. The subject must be followed until resolution of the event as with any adverse event

- Acute disease at the time of vaccination. (Acute disease is defined as the presence of a moderate or severe illness with or without fever.) All vaccines can be administered to persons with a minor illness such as diarrhoea, mild upper respiratory infection with or without low-grade febrile illness, *i.e.*, temperature of <37.5°C/99.5°F).
- Temperature of ≥37.5°C (99.5°F) at the time of vaccination.

5 VACCINES

5.1 Investigational Vaccine Characteristics: FP9 and MVA ME-TRAP

5.1.1 Description

MVA ME-TRAP and FP9 ME-TRAP, manufactured at Impfstoffwerk Dessau-Tornau (IDT), Germany by Oxford University.
Both the Modified Vaccinia Virus Ankara (MVA) ME-TRAP and fowlpox 9 (FP9) vaccines express a fusion protein of multiple epitopes (ME) and the pre-erythrocytic antigen thrombospondin-related adhesion protein (TRAP).

5.1.2 Formulation

FP9 ME-TRAP will be provided in vials of 225 μL volume at a concentration of 1.8×10^9 pfu/mL in 10 mM Tris buffer. The dose of FP9 ME-TRAP to be used in this study will be 1.0×10^8 pfu, to be given intradermally in a volume of 60 μL.
MVA ME-TRAP will be provided in vials of 300 μL volume at a concentration of 5×10^8 pfu/mL in 10 mM Tris buffer. The dose of MVA ME-TRAP to be used in this study will be 1.5×10^8 pfu, to be given intradermally in a volume of 300 μL.

5.1.3 Precautions for use

The skin at the injection site will be prepared with an alcohol wipe before injection. The investigator will use gloves and eye protection, and will advise the subject not to touch or rub eyes, if touched. Subjects will stay in the unit for 30 minutes after each vaccination for observation. During administration of the vaccines, medicines and resuscitation equipment will be immediately available for the management of anaphylaxis.

5.2 Investigational Vaccine Characteristics: PEV3A

5.2.1 Description

PEV3A is a virosomal vaccine preparation manufactured by Pevion Biotech Ltd., Switzerland. The vaccine carries two synthetic *P. falciparum* antigens derived from the circumsporozoite (CS) and apical membrane antigen-1 (AMA-1) proteins.

5.2.2 Formulation

PEV3A will be provided in vials of 0.5 mL containing 50 μg of PEV301 and 10 μg of PEV302 in phosphate buffered saline (pH 7.4). The dose of PEV3A to be used in this study will be 0.5 mL, to be given intramuscularly.

5.2.3 Precautions for use

The skin at the injection site will be prepared with an alcohol wipe before injection. Subjects will stay in the unit for 30 minutes after each vaccination for observation. During administration of the vaccines, medicines and resuscitation equipment will be immediately available for the management of anaphylaxis.

5.3 Vaccination

FP9 and MVA ME-TRAP vaccines will be given by intra-dermal injection into the skin overlying the deltoid muscle in the right arm in preference. However, if volunteers have a specific reason it may be given into the left arm if requested. When more than one injection per arm is necessary due to the volume to be administered, skin puncture sites will be limited to an area of 36 square centimetres.

PEV3A will be given by intramuscular injection into the deltoid muscle in the left arm for all groups in preference. However, if volunteers have a specific reason for requesting it (eg left handedness), it may be given into the right arm.

In order to minimise dissemination of the recombinant vectored vaccine virus into the environment the inoculation site will be covered with a dressing after immunisation. This should absorb any virus that may leak out through the needle track. The dressing will be removed from the injection site at the end of the 30 minutes observation period and will be disposed as GMO Waste by autoclaving. This is in accordance with our GMO Standard Operating Procedures (see Appendix C.8) and current standard UK practice.

5.4 Prior and Concomitant Therapy

The following treatments are exclusion criteria, use of other medications is permitted :

Prior receipt of an investigational malaria vaccine
Use of any investigational or non-registered drug, live vaccine or medical device other than the study vaccine within 30 days preceding dosing of study vaccine, or planned use during the study period
Administration of chronic (defined as more than 14 days) immunosuppressive drugs or other immune modifying drugs within six months of vaccination. (For corticosteroids, this will mean prednisolone, or equivalent, ≥ 0.5 mg/kg/day. Inhaled and topical steroids are allowed.)
History of malaria chemoprophylaxis with chloroquine within 5 months prior to the planned challenge, with Lariam within 6 weeks prior to the challenge, and Riamet® within 2 weeks prior to the challenge

All subjects will be asked about medication at screening and at each subsequent visit. All medication taken will be recorded on the concomitant medications page of the case report forms. The following details will be recorded :

Trade name
Total daily dose
Start and stop dates
Indication

5.5 Management of Vaccines

5.5.1 Labelling and Packaging

Vaccines will be supplied in multi-dose vials. All vaccines are labelled according to national regulations. The labels will be made up using the following template:

**CLINICAL TRIAL: VAC030**<br>Investigator: Prof. Adrian Hill<br>******VACCINE **** mL**<br>(containing*******)<br>For Intradermal/Intramuscular Injection<br>Batch no: ****** Retest Date: ********<br>STORE AT ***** °C<br>Volunteer no: M1030___

VACCINE – either 'MVA ME-TRAP', 'FP9 ME-TRAP' or 'PEV3A'

5.5.2 Storage and Shipment Conditions

Vaccines will be stored in the Churchill Hospital pharmacy. PEV3A will be stored in the pharmacy refrigerator at +2 to +8°C. MVA and FP9 ME-TRAP will be stored at a temperature of -18°C or below in the pharmacy freezer. Temperature will be monitored and documented following the pharmacy guidelines. In case of accidental disruption of the cold chain, vaccines should never be administered and the investigator or the responsible person should report the event to the Monitor. Vaccines will be dispensed from Pharmacy in advance and stored in a secure, alarmed refrigerator or freezer in the Centre for Clinical Vaccinology and Topical Medicine. On vaccination day frozen vaccines will be allowed to thaw to room temperature and administered within 1 hour.

5.5.3 Accountability

Products will be kept in a secure place. The investigator or the person in charge of product management, will maintain records of the product's delivery to the trial site, the inventory at the site and the dose(s) given to each subject.

6 CHALLENGE PHASE

6.1 Biting Procedure with *P. falciparum*-Infected Anopheles Mosquitoes

Up to five mosquitoes are placed in a cardboard carton, covered with netting, for each subject. The subject rests his or her forearm on the screened top of the carton. The mosquitoes are allowed to feed undisturbed for 5 minutes.

Fed mosquitoes, as indicated by the presence of a blood meal in the abdomen, are individually dissected, and the paired salivary glands of each fed mosquito are transferred to a microscope slide. A cover slip is placed on the slide and the salivary glands are gently squashed to release the sporozoites.

Under microscopy, glands are rated 0 to +4 according to the gland rating index for sporozoites: a mosquito with a gland rating of +2 or more qualifies as being infective. The assessment is based on the following scale.

Gland rating	No. sporozoites
0	No sporozoites observed
+1	1 – 10 sporozoites observed
+2	11 – 100 sporozoites observed
+3	101 – 1000 sporozoites observed
+4	>1000 sporozoites observed

If by this method the volunteer is found to have been inoculated by less than five infected mosquitoes, further infected mosquitoes will be allowed to feed on the volunteer until a total of 5 infected mosquitoes have fed. New mosquitoes are added to a carton depending on the number of infective bites still required.

The bite-challenge procedure continues until the subject receives 5 infective bites.

6.2 Anopheles Strain, Origin and Culture Conditions

Anopheles stephensi (originally obtained from Nijmegen-strain SF500) are infected with the cultured material 17 days from the beginning of gametocyte culture when sufficient mature gametocytes are present, as indicated by the ability of the microgametocytes to exflagellate. The mosquitoes are fed via a membrane feeder containing parasite material plus fresh red cells from one of the donors and the AB human serum. The blood products come from volunteers who have been screened as per UK Blood Bank (antibodies for HIV, HBV, HCV and syphilis).

6.3 *Plasmodium* Strain, Origin and Culture Conditions

The *P. falciparum* used at Imperial College is the 3D7 clone of strain NF54, isolated by Prof. D. Walliker. Work carried out at London School of Hygiene and Tropical Medicine has shown that 3D7 is reasonably sensitive *in vitro* to both lumefantrine and artemether and a marked synergism between lumefantrine and artemether has been observed (Prof. Warhurst, personal communication), so use of

the Riamet® combination is considered to be rational. 3D7 is maintained in a continuous culture system in a medium containing 10% v/v human AB serum (from the Blood Transfusion Service, Colindale, UK) heat inactivated at 56°C for 30 min. The blood products come from volunteers who have been screened as per UK Blood Bank (antibodies for HIV, HBV, HCV and syphilis).
Simultaneously, a batch of *P. falciparum*-infected *Anopheles stephensi* mosquitoes will be prepared by the Walter Reed Army Institute of Research, USA and provided as a back up for the challenge. Similarly, mosquitoes will be infected with the 3D7 clone of the NF54 strain isolated by Prof. D. Walliker. A large master seed lot of 3D7 has been prepared for the human studies under GMP conditions and only parasites from this master seed will be used to infect mosquitoes.

6.4 Anopheles Infection and Storage

Twenty-four hours after feeding, the unfed mosquitoes are removed and the remainder maintained on a fructose/paba solution. 7-9 days after the infective feed, samples of the mosquitoes are checked for the presence of oocysts. Several days before challenge of the volunteers, samples are checked again for the presence of sporozoites in the salivary glands; they are then transferred to small pots containing 1-5 mosquitoes for feeding on the volunteers.

6.4.1 Investigational agent accountability

Infected mosquitoes are held in a secure room within the insectary and each pot of mosquitoes is kept in a strong transparent plastic storage box. All manipulations of mosquitoes are done in a secure Perspex cabinet. The small pots of mosquitoes brought out for challenge of the volunteers are covered with netting and held in a second pot to minimise any possibility of escape. The challenge is performed in a part of the insectary that is isolated by vacuum traps**.**

6.5 Toxicity Management

Subjects that develop local itch at the site of the mosquito bites that causes discomfort will be prescribed topical 1% hydrocortisone cream or topical antihistamine cream for use twice daily for 3 days. If the subjects develop headaches, fever, chills, myalgia or backache that is considered possibly due to the experimental malaria infection then paracetamol (1 g orally up to four times a day) will be prescribed for 3 days.
If nausea causes discomfort, then cyclizine (50 mg orally three times a day) will be prescribed for two days. If a subject presents with any of the signs described in Section 8.3 they will be admitted to John Warin Ward for further management

7 Management of Malaria Cases

7.1 Malaria Assessment

When a case is identified, either asymptomatic malaria or clinical malaria, each subject will have a clinical evaluation by one of the investigators (a physician) with appropriate history and physical examination where deemed to be necessary. If they have either no symptoms or mild to moderate symptoms, they will be managed as outpatients. If they have moderate to severe symptoms or signs they may be admitted to the John Warin infectious disease ward at the Churchill Hospital for observation and further medical management under the care of Dr. Tim Peto or Dr. Chris Conlon (Consultants in infectious diseases).

Subjects will have a symptom questionnaire administered by the investigator or the Clinical Research Nurse daily for 2 days after the endpoint is reached. If symptoms are present they will have a clinical evaluation with a history and physical examination.

7.2 Treatment

Volunteers will be treated with oral Riamet®. This is a licensed drug in the UK for treatment of acute uncomplicated malaria caused by *Plasmodium falciparum* and has achieved a cure rate of 97.7% [55]. It is a combination drug consisting of artemether (20 mg) and lumefantrin (120 mg) per tablet. A treatment course of Riamet® consists of 6 times 4 tablets. The first 4 tablets will be given when diagnosis is made, followed by additional doses after 8, 24, 36, 48 and 60 hours. Tablets should be taken together with a meal. Treatment will be observed on three occasions at least and slide reading will be continued until two consecutive slides are negative for parasites.

Volunteers who remain film negative at Day 21 will be considered protected from malaria but will still receive Riamet® treatment as a precaution. The course will be administered and observed as above.

7.3 Criteria for Hospital Admission to the John Warin (Infectious Diseases) Ward

Any one of the following:
Failure of symptoms to improve within 48 hours of starting Riamet® therapy
Unable to tolerate oral Riamet®
Dehydration requiring intravenous fluid therapy
Signs or symptoms suggestive of pulmonary oedema
Signs or symptoms of neurological dysfunction including altered consciousness
Signs, symptoms or laboratory evidence of renal failure
Haemoglobinuria
Severe symptoms of malaria defined as preventing Activities of Daily Living
Unanticipated concern about subject's home circumstances

Ultimately the decision regarding admission will be taken by the investigators in conjunction with the infectious diseases consultants.

Of 164 volunteers who have undergone malaria challenge in previous studies, one volunteer was admitted to the ward for 48 hours due to vomiting, and received intra-venous fluids and anti-emetics as additional therapy; another study subject was admitted for 12 hours with facial and eyelid oedema that resolved spontaneously and was considered to be possibly related to the treatment with chloroquine.

8 Adverse event Management and reporting

8.1 Definitions

Definitions for the terms adverse event (or experience), adverse reaction, and unexpected adverse reaction have previously been agreed to by consensus of the more than 30 Collaborating Centres of the WHO International Drug Monitoring Centre (Uppsala, Sweden) [56]. Although those definitions can pertain to situations involving clinical investigations, some minor modifications are necessary, especially to accommodate the pre-approval, development environment.
The following definitions, with input from the WHO Collaborative Centre, have been agreed:

8.1.1 Adverse Event (or Adverse Experience)

Any untoward medical occurrence in a patient or clinical investigation subject occurring in any phase of the clinical study whether or not considered related to the vaccine or to the malaria challenge. This includes an exacerbation of pre-existing conditions or events, intercurrent illnesses, or vaccine or drug interaction. Anticipated day-to-day fluctuations of pre-existing conditions, including the disease under study, that do not represent a clinically significant exacerbation will not be considered adverse events. Discrete episodes of chronic conditions occurring during a study period will be reported as adverse events in order to assess changes in frequency or severity.

Events that can reasonably be expected as part of this study (symptoms and signs of malaria such as fever, myalgia, malaise, arthralgia, anorexia, nausea and diarrhoea which are temporally related to the diagnosis of malaria), and are mild moderate or severe will only be documented on the symptom questionnaire of the CRF, not on the adverse event form. However, all other adverse events will be reported on the Adverse Event form in the subject's CRF. During the overlap period, their relationship to the vaccine will be assessed. All serious adverse events, including those believed to be secondary to malaria infection, will be reported as indicated below.

Adverse events will be documented in terms of a medical diagnosis(es). When this is not possible, the adverse event will be documented in terms of signs and symptoms observed by the investigator or reported by the subject at each study visit.

Pre-existing conditions or signs and/or symptoms (including any which are not recognised at study entry but are recognised during the study period) present in a subject prior to the start of the study will be recorded on the Medical History form within the subject's CRF.

8.1.2 Adverse Drug Reaction (ADR)

In the pre-approval clinical experience with a new medicinal product or its new usages, particularly as the therapeutic dose(s) may not be established: all noxious and unintended responses to a medicinal product related to any dose should be considered adverse drug reactions.
The phrase "responses to a medicinal product" means that a causal relationship between a medicinal product and an adverse event is at least a reasonable possibility, *i.e.*, the relationship cannot be ruled out.

8.1.3 Unexpected Adverse Drug Reaction

An adverse reaction, the nature or severity of which is not consistent with the applicable product information (*e.g.*, Investigator's Brochure for an unapproved investigational medicinal product) is considered as an unexpected adverse drug reaction.

8.1.4 Serious Adverse Event

A serious adverse event (experience) or reaction is any untoward medical occurrence that at any dose:

- results in death,
- is life-threatening,
  Note: The term "life-threatening" in the definition of "serious" refers to an event in which the patient was at risk of death at the time of the event; it does not refer to an event which hypothetically might have caused death if it were more severe.
- requires inpatient hospitalisation or prolongation of existing hospitalisation,
- results in persistent or significant disability/incapacity, or
- is a congenital anomaly/birth defect.

Medical and scientific judgment should be exercised in deciding whether expedited reporting is appropriate in other situations, such as important medical events that may not be immediately life-threatening or result in death or hospitalisation but may jeopardise the patient or may require intervention to prevent one of the other outcomes listed in the definition above. These should also usually be considered serious.

8.2 Safety Data Collection and Management Procedures

8.2.1 Expected Adverse Vaccine Reactions

See Section 9.2.

8.2.2 Safety Data Collection

See Section 9.2.

8.2.3 Follow-up of Adverse Events

Adverse events will be recorded as indicated in the CRF.
Adverse events likely to be related to the vaccine, whether serious or not, which persist at the end of the trial will be followed up by the investigator until their complete disappearance.
Moreover, any serious adverse event likely to be related to the vaccine and occurring after trial termination should be reported by the investigator according to the procedure described below.

Outcome of any non-serious adverse event occurring within 30 days post-vaccination (*i.e.* unsolicited adverse event) or any SAE reported during the entire study will be assessed as:
Recovered/resolved
Not recovered/not resolved
Recovering/resolving
Recovered with sequelae/resolved with sequelae
Fatal (SAEs only)

8.3 Reporting of Serious Adverse Events (SAE)

Every SAE occurring throughout the trial must be reported to the sponsor by the investigator as **soon as he is alerted of it** and within 24 hours, even if the investigator considers that the adverse event is not related to vaccination, or to the challenge. All SAEs will be reported by telephone, email or fax to the local safety monitor, OxREC, and sponsor within one working day of the investigator becoming aware of the SAE occurrence. The investigator will then complete a SAE report form as soon as possible and within five working days or seven calendar days.
Any relevant information concerning the adverse event that becomes available after the SAE report form has been sent (outcome, precise description of medical history, results of the investigation, copy of hospitalisation report, etc.) will be forwarded to the Sponsor in a timely manner. The anonymity of the subjects shall be respected when forwarding this information.

Suspected unexpected serious adverse reactions will be reported to the MHRA within 7 days. The sponsor pledges to inform the Authorities of any trial discontinuation and specify the reason for discontinuation.

A blood sample to be taken as soon as possible might be requested in case of serious adverse event if it may help in analysing the SAE. Five mL of blood will be taken in a dry tube.

The causal relationship between the SAE and the product will first be evaluated by the investigator with the following scale:

0. No relationship:
- No temporal relationship to study product; *and*
- Alternate aetiology (clinical state, environmental or other interventions); *and*
- Does not follow known pattern of response to study product

1 . Possible relationship:
- Reasonable temporal relationship to study product; *or*
- Event not readily produced by clinical state, environmental or other interventions; *or*
- Similar pattern of response to that seen with other vaccines

2. Probable relationship:
- Reasonable temporal relationship to study product; *and*
- Event not readily produced by clinical state, environment, or other interventions *or*
- Known pattern of response seen with other vaccines

3. Definite relationship:
- Reasonable temporal relationship to study product; *and*
- Event not readily produced by clinical state, environment, or other interventions; *and*
- Known pattern of response seen with other vaccines

8.4 Post-trial Evaluations

Subjects who have moderate or severe on-going adverse events at the completion of the study will be advised to consult their General Practitioner (National Health Service) if the event is not considered to be related to the study vaccine. A follow-up visit will be arranged to manage the problem and to determine the severity and duration of the event, if it is considered to be related to the study vaccine. If appropriate, specialist review within the National Health Service (NHS) will be arranged. In the UK all subjects receive free healthcare under the NHS.

8.5 Pregnancy

Subjects who become pregnant during the study before receiving the last vaccine dose must not receive additional doses of study vaccine but may continue other study procedures at the discretion of the investigator.

The investigator, or his/her designee, will collect pregnancy information on any subject who becomes pregnant while participating in this study. The subject will be followed to determine the outcome of the pregnancy.

9 Evaluation Criteria

9.1 Primary Evaluation Criteria

9.1.1 Definition of the Criteria

The protection against malaria infection in a sporozoite challenge model will be assessed by measuring the number of subjects who develop malaria infection and the time in hours between exposure and parasitaemia as detected by thick-film blood smear. The comparison will be between the three vaccine groups and between vaccinees and unvaccinated controls.

End-points:
Primary: One thick smear positive for one or more parasites during the 21 day observation period,
OR
Secondary: Subject is manifesting typical clinical symptoms or signs of malaria in the opinion of the Principal Investigator (such as fever >37.5°C, rigors, moderate or severe myalgia, in the absence of another obvious cause) and has negative blood smears but has a PCR result that is positive for malaria.

9.1.2 Parameters to be Measured

9.1.2.1 Clinical Symptoms

9.1.2.1.1 Definitions:

No solicited adverse events will be recorded, but the following predictable symptoms of malaria will be enquired about at each visit:

- Symptoms of feverishness
- Chills
- Rigors
- Sweats
- Headache
- Anorexia
- Nausea / vomiting
- Diarrhoea
- Myalgia /Arthralgia
- Low back pain

Subjects will be instructed to record their oral temperature with the thermometer provided should they feel feverish.

The subjects will be instructed to contact the investigator immediately should they manifest any signs or symptoms they perceive as serious.

9.1.2.1.2 Assessment of Severity

Intensity of the general adverse events will be assessed as described:

GRADE 0	None
GRADE 1	Mild: Transient or mild discomfort (< 48 hours); no medical intervention/therapy required
GRADE 2	Moderate: Mild to moderate limitation in activity - some assistance may be needed; no or minimal medical intervention/therapy required
GRADE 3	Severe: Marked limitation in activity, some assistance usually required; medical intervention/therapy required, hospitalisation possible
GRADE 4	Serious, life-threatening: Extreme limitation in activity, significant assistance required; significant medical intervention/therapy required, hospitalisation or hospice care probable (see also Section 8.1.4.)

9.1.2.1.3 Assessment of Outcome

Outcome will be assessed as described for general adverse events (see Section 8.2.3).

9.1.2.2 Parasitological Parameters

The parasitaemia will be assessed by thick smears and PCR. A maximum of 27 blood smears and PCR will be performed for each volunteer. From 6 days after the day of challenge, the samples will be taken twice a day (approximately 12 hourly) for 7 days (evening of D83 – D91). From then onwards, samples will be taken once daily until D100 (23 days after malaria infection).
After a positive blood film (and commencement of treatment) blood samples will be collected daily until two consecutive negative thick smears are obtained. These will be examined by blood smear immediately. PCR analysis will be done until the first positive thick smear.
The procedure for thick film preparation and definition of negativity is described in Appendix C.9.

9.1.3 Timing of Measurement

Clinical assessment and measurement of vital signs will be performed and recorded at each visit.

9.2 Secondary Evaluation Criteria

9.2.1 Safety: Definition of the Criteria

The safety profile of the vaccines will be assessed on the following criteria

- Immediate reactogenicity (reactions within 30 minutes after each injection, with emphasis on allergic reaction),
- Local and systemic reactogenicity measured from day 0 to day 28 after each dose
- Any unsolicited adverse event resulting in a visit to a physician between each injection and one month after the third dose.
- Any Serious Adverse Event (SAE) occurring from the inclusion throughout the study. See Section 8.3.

- Biological safety, one week and four weeks after each vaccination, in reference with the baseline before the first dose, by measuring the following:
  - Full blood count (FBC)
  - Urea, potassium, sodium, ALT, total bilirubin, alkaline phosphatase, albumin, creatinine.

9.2.2 Parameters to be Measured

The solicited clinical signs and symptoms are listed in Table 3~~Table 3~~ below.

Formatted: Font: 12 pt

Table 3: Solicited local and general adverse events documented to the CRF

	**Adverse events**
**Local (injection site)**	Pain at the injection site
	Redness at the injection site
	Swelling at injection site
	Warmth at the injection site
	Itch at the injection site
	Scaling/Pustules at site
**General**	Documented fever (oral temperature > 37.5° C)
	Symptoms of feverishness
	Malaise
	Arthralgia
	Headache
	Myalgia
	Nausea / vomiting
	Other (specify)

9.2.3 Method and Timing of Measurement

The safety criteria will be assessed 30 minutes following the vaccination, and during the 7 following days.
At each visit vital signs will be documented on the CRF together with local reactions at the injection site. The largest diameter through the injection site of any redness will be recorded in millimetres. The largest diameter through the injection site of local swelling, defined as a more generalized swelling of the deltoid muscle will be recorded in millimetres. Severity of these local findings will be graded using the scale given in Table 4~~Table 4~~.

Table 4: Grading a) for swelling

Grade	**Diameter [mm]**
0	0
1	< 20
2	21 – 50
3	> 50

b) for redness

Grade	**Diameter [mm]**
0	0
1	< 50
2	51 – 100
3	> 100

Study subjects will be asked to indicate the maximum degree of pain they experience at the injection site using a scale ranging from 0 to 3 as described in Table 5~~Table 5~~

Table 5: Pain scale

Grade	Description
**0**	No pain at all
**1**	Painful on touch, no restriction in movement of arms, able to work, drive, carry heavy objects as normal
**2**	Painful when limb is moved (*i.e.* restriction in range of movement in arm, difficulty in carrying objects)
**3**	Severe pain at rest (*i.e.* unable to use arm due to pain.)

All local reactions will be considered causally related to the vaccination.

At each visit subjects will be requested to report local and general side effects they might have experienced since they last were seen. The investigator will assess the severity of the solicited signs and symptoms using the key provided in Section 9.1.2.1.2.

The investigator using the guidelines provided in section 8.3 will assess the relationship of the event to the administration of the vaccine. Both severity of the event and its relationship to the vaccine administration will be documented in the CRF.

Further details for any solicited AE (such as start/stop date and any treatment), will also be gathered, no matter whether or not it is considered related to the vaccine. On the CRF space is allocated to document any unsolicited adverse event reported by the volunteer. Serious adverse events (SAE) as defined in section 8.1.4 of the protocol will be documented in the CRF and reported by the lead investigator to the local safety monitor within 24 hours of becoming aware of the event, whether or not this event is considered to be related to the study vaccine.

9.2.4 Immunogenicity: Definition of the criteria

Immunological assays will be conducted according to the procedures established in the test laboratories. With the volunteers' informed consent, any leftover cells and serum will be frozen for future immunological analysis of malaria, including intracellular cytokine staining techniques, proliferation assays and assessment of anti-vector responses. All exploratory immunology carried out at the discretion of the investigator will be to study the body's immune response to malaria vaccination and malaria. These studies will look for markers of response to vaccination or protective immunity to malaria. No studies concerning diseases other than malaria will be undertaken.

9.2.5 Cell Mediated Immunity (CMI)

IFN-γ ELISPOT will be performed on PBMCs obtained from each volunteer. These will be separated from samples taken for exploratory immunology. Some of the ELISPOT tests may be run in batches after the study using thawed PBMCs.

For the ex vivo IFN-γ ELISPOT freshly isolated PBMC are stimulated with malaria peptides, control peptides, recombinant antigens, or recombinant viruses expressing part or all of the ME-TRAP insert in ELISPOT plate (Millipore) wells coated with antibody specific for human IFN-γ (Mabtech). After 18-20 hours incubation the PBMC are washed away and biotinylated anti-IFN-γ added, followed by alkaline phosphatase-conjugated streptavidin (both from Mabtech). Capture of IFN-γ can then be visualized by adding chromogenic substrate (Bio-Rad). The coloured spots are calculated with an ELISPOT reader (AID) and the results are expressed as spot forming units (SFU) per 1×10^6 PBMC.

9.2.6 Antibody Measurements

Anti-CS and anti-AMA-1 antibodies will be measured. Blood for serology will be collected throughout the trial as described on pages xiv - xvi~~xviii~~.

9.2.7 Gene Expression Studies

In addition, with volunteers' express permission, samples will be collected for gene expression studies (see pages xiv - xvi for the schedule of blood collection and volumes drawn). Whole genome high-density arrays may~~will~~ be used to compare gene expression at baseline and after vaccination, looking for important markers of response to vaccination and protection against malaria. No studies concerning diseases or traits not connected with malaria will be done.

9.3 Tertiary Evaluation Criteria

Protected volunteers will be involved in a late challenge, in order to assess the tertiary objective. The protection against malaria infection in a sporozoite challenge model will be assessed by measuring the number of subjects who develop malaria infection and the time in hours between exposure and parasitaemia as detected by thick-film blood smear. The comparison will be between the vaccine groups and unvaccinated controls. The outcome of this will be assessed as described above for the primary evaluation criterion.

10 Biometry

10.1 Determination of the Sample Size

This is primarily a descriptive study and the first, preliminary examination of efficacy of the virosomal vaccine PEV3A against malaria infection. It is difficult to estimate the potential size of any beneficial effect of PEV3A and we have therefore used our past experience of similar trials to decide on numbers in each group. We have included the maximum number of volunteers that we can manage appropriately at one time.

10.2 Data Set to be analysed

10.2.1 Definition of Population

We will enrol 48 subjects, 12 per group for vaccinations, as well as 12 volunteers to act as controls for the challenge (6 for early challenge, 6 for late challenge)

10.2.2 Population used in analyses

All subjects who have been challenged will be included in the analysis. Unvaccinated subjects who are challenged will document the efficacy of the challenge system, and will form the control group for analysis of vaccine efficacy.

10.2.3 Statistical Methods

The main analysis for the primary objective will be based on the number of hours between infectious challenge and blood stage parasitaemia. Each of the three groups will be compared with each other and with the control group using the Kaplan Meier Method. Statistical significance of any differences observed can then be assessed by the log rank test.

If there is complete protection a 95% confidence interval for a single proportion will be calculated using the method recommended by Wilson for small sample sizes. Due to the small sample size testing for significant differences between two proportions will be done using Fisher's exact test and by calculating a confidence interval for the observed difference.

Within the secondary objective, safety and tolerability is only descriptive and will therefore not be analysed. Rates of adverse reactions will be compared with those in previous studies using these vaccines. Immunogenicity will be assessed by comparing the sum of the interferon-γ ELISPOT responses to malaria peptides and anti-AMA-1 and anti-CS antibodies between the three vaccination and one control groups. Results will be analysed for correlation to any delay in parasitaemia. Further sub-analysis of responses to individual peptides may be performed. The number of spots will be

normalized for the number of cells per well, the number of lymphocytes in the peripheral blood and the baseline number of spots.

10.2.4 Data Management

The Lead Investigator will be the data manager with responsibility for receiving, entering, cleaning, querying, analysing and storing all data that accrues from the study. The data will be entered into the subjects' CRFs which will be electronic, stored on a database developed with StudyBuilder™ software. For more details see Appendix C.7.

If any changes to the protocol are necessary during the study a formal amendment will be presented to the sponsor prior to submission to OxREC for approval. Any unforeseen and unavoidable deviations from the protocol will be documented and filed in a protocol deviation folder, with explanation.

11 ETHICAL CONSIDERATION

11.1 Informed Consent

The subject should give written informed consent before being included in the trial, after having been informed of the nature of the trial, the potential risks and their obligations. Informed consent forms will be provided in duplicate (original kept by the investigator, one copy kept by the subject or the subject's legally acceptable representative).

11.2 Subject Benefit and Risks

Volunteers will not benefit directly from participation in this study. However, it is hoped that the information gained from this study will contribute to the development of a safe and effective malaria vaccine.

The general risks to participants in this study are associated with phlebotomy, vaccination and those associated with malaria infection. The volume of blood drawn over the study period (766 mL for groups 1 and 2, 784 mL for group 3 and 380 mL for the control group) should not compromise these otherwise healthy subjects.

11.3 Ethical Review

Before the inclusion of the first subject in the study, the protocol must be approved by OxREC (Oxfordshire Research Ethics Committee).

11.4 Good Clinical Practice

This trial will be conducted in accordance with the Declaration of Helsinki as agreed by the World Medical Association General Assembly (Washington 2002), ICH Good Clinical Practice and local regulatory requirements.

12 REGULATORY

PEV3A has been developed by Pevion Biotech Ltd. and is manufactured under GMP conditions by Berna Biotech Ltd. (Rehhagstrasse 79, CH - 3018 Bern, Switzerland).

The MVA and FP9 vaccines being used were developed by Oxford University and manufactured under GMP conditions by the contract manufacturer Impfstoffwerk Dessau-Tornau GmbH (IDT, Rosslau, Streetzer Weg PSF 214, 06855 Rosslau, Germany).

All vaccines will be used under a CTA (Clinical Trial Authorisation) provided by the MHRA of the United Kingdom.

13 Quality Control and Quality Assurance

13.1 Direct Access to Source Data/Documents

The principal investigator will provide direct access to the source data documents to the Ethics Committee, to the regulatory agency, and to the sponsor, permitting trial-related monitoring, and audits.

13.2 Modification of the Protocol

No amendments to this protocol will be made without consultation with, and agreement of, the sponsor. Any amendments to the trial that appear necessary during the course of the trial must be discussed by the investigator and sponsor concurrently. If agreement is reached concerning the need for an amendment, it will be produced in writing by the sponsor and/or the investigator and will be made a formal part of the protocol. An amendment requires Ethics Committee approval.

All amendments must also be transmitted to Regulatory Authorities, if applicable.

An administrative change to the protocol is one that modifies administrative and logistical aspects of a protocol but does not affect the subjects' safety, the objectives of the trial and its progress. An administrative change does not require Ethics Committee approval. However, the Ethics committee must be notified whenever an administrative change is made.

The investigator is responsible for insuring that changes to an approved trial, during the period for which Ethics Committee approval has already been given, are not initiated without Ethics Committee review and approval except to eliminate apparent immediate hazards to the subject.

13.3 Termination of the Trial

Every reasonable effort should be made to maintain protocol compliance and participation in the study. If a subject is prematurely terminated from the study for any reason, the reason for early study withdrawal will be recorded. If withdrawal is the result of a serious AE, the investigator will offer to arrange for appropriate specialist management of the problem under the National Health Service and OxREC will be informed in a timely manner. The extent of follow up after premature discontinuation will be determined by the investigator but will be at least for the whole study period. Subjects withdrawn prematurely for any reason will not be re-entered into this protocol, although they may be requested to return to the clinic for safety evaluation. A complete safety evaluation will be made for any subject who terminates from the study prematurely.

The following will result in study termination and will not be considered normal protocol completion:

Developed an AE. Applies to a subject who is withdrawn from the study primarily due to a severe or serious adverse event.

Lost to follow-up - applies to a subject who does not return for protocol study visits, is not reachable by telephone or other means of communication and/or is not able to be located.

Research terminated by investigator - applies to the situation where the entire study is terminated by the investigator for any reason.
Withdrawal of Consent - applies to a subject who withdraws consent to participate in the study for any reason.
Protocol violation - applies to a subject who fails to achieve critical endpoints or does not meet entrance criteria.
Non-compliant with protocol - applies to a subject who does not comply with protocol-specific visits or evaluations even though the subject is able to comply.
Other - is a category used when previous categories do not apply and requires an explanation.

13.4 Investigator Procedures

See Appendix C.1.

13.5 Monitoring

13.5.1 Initiation Visit

An initiation visit will be performed before the inclusion of the first subject in the study. The Monitor will verify and document that the material to be used during the trial has been received and that the investigational team has been properly informed about the trial and regulatory requirements.

13.5.2 Follow-up Visits

The Monitor will carry out regular follow-up visits. The investigator commits to being available for these visits and to allow the monitoring staff direct access to subject medical files and CRFs. The Monitor is committed to professional secrecy.
During the visits, the Monitor will:
carry out a quality control of trial progress: respect of protocol and operating guidelines, data collection, signature of consent forms, completion of document and appearance of SAE, sample and product management, cold chain monitoring
collect the CRFs and correspondent correction sheets
assess the inclusions in order to evaluate the number of complete or on-going observations
The Monitor will discuss any problem with the investigator and define with him the actions to be taken.

13.5.3 Close-out Visit

A close-out visit will be performed at the end of the trial. Its goals are to make sure that:
the centre has all the documents necessary for archiving
all unused material has been recovered

13.6 Audits and Inspections

If necessary, a quality assurance audit will be carried out by independent auditors to make sure that the trial has been conducted according to the protocol and the applicable regulations.
An inspection may be conducted by Regulatory Authorities.
The investigator must allow direct access to trial documents.

13.7 Archiving

The investigator must keep all trial documents for at least 15 years after the completion or discontinuation of the trial, whatever the nature of the investigational centre (private practice, hospital, institution).

14 Financing and Insurance

14.1 Compensation for participation

Volunteers will be compensated for their time and travel for participation in the study as detailed below:

Groups 1 & 2 (41 visits):

Compensation per visit (travel + time):	41 x £20	= £820
Compensation for Blood sampling:	37 x £6	= £222
Compensation for challenge day:	1 x £170	=£170
Compensation for 3 days off (malaria)	3 x £100	= £300
**Total:**		**= £1512**

Group 3 (47 visits):

Compensation per visit (travel + time):	47 x £20	=£940
Compensation for blood donation:	40 x £6	=£240
Compensation for challenge day:	1 x £170	=£170
Compensation for 3 days off (malaria)	3 x £100	=£300
**Total:**		**=£1650**

Control group (30 visits):

Compensation per visit (travel + time):	30 x £20	=£600
Compensation for blood donation:	30 x £6	=£180
Compensation for challenge day:	1 x £170	=£170
Compensation for 3 days off (malaria)	3 x £100	=£300
**Total:**		**=£1250**

Late challenge (29 visits)

Compensation per visit (travel + time):	29 x £20	=£580
Compensation for blood donation:	29 x £6	=£174
Compensation for challenge day:	1 x £170	=£170
Compensation for 3 days off (malaria)	3 x £100	=£300
**Total:**		**=£1224**

Volunteers who withdraw from the study early will still be compensated, but compensation will be adjusted using the key outlined above. Subjects that only come for screening will get a compensation of £26. Compensation will be paid within 8 weeks of the subject's final visit.

14.2 Insurance

Oxford University investigators participating in this trial will receive insurance coverage from the University clinical trials insurance policy.

APPENDIX A: LABORATORY VALUES FOR EXCLUSION

PARAMETER	LOWER LIMIT OF EXCLUSION	UPPER LIMIT OF EXCLUSION
**BIOCHEMISTRY**		
Potassium [mmol/L]	<3.2	>5.5
Sodium [mmol/L]	<132	>148
Urea[mmol/L]	N/A	>9
Creatinine [μmol/L]	N/A	>180
Albumin [g/L]	<30	N/A
Tot. Bilirubin [μmol/L]	N/A	>25
ALT [IU/L]	N/A	>60
ALP [IU/L]	N/A	>350
**HAEMATOLOGY**		
Haemoglobin [g/dL]	Male: < 11.5<br>Female: < 10.5	Male: > 18<br>Female: >17.5
White Cell Count [x 10^9/L]<br>Neutrophil count [x 10^9/L]	<3.0<br>< 1.5	>14.0
Platelet Count [x 10^9/L]	<130	>500

Urine analysis (using MULTISTIX * 10 SG Bayer Diagnostics)	
Protein [g/L]	> 0.3
Glucose [mmol/L]	> 5.5

APPENDIX B: TEXT FOR RADIO ADVERTISEMENT

Voice over: *'More than two million people die each year from malaria and an effective vaccine is desperately needed. New vaccines against the disease are currently being tested at Oxford University.*

'We're now looking for healthy adult volunteers who are willing to help test the vaccines. Volunteers must be aged 18 to 50, live in the Oxford area and must not have had malaria before. Expenses, travel and time will be compensated.

'For more details call the Malaria Vaccine Study Group on Oxford 857401'*

Approved by the Oxfordshire Research Ethics Committee.
* pronounced 'eight five seven four oh one'

APPENDIX C: INVESTIGATOR STANDARD OPERATING PROCEDURES

C.1 Visit Procedures

Entry

Subjects who match all inclusion and exclusion criteria and are available for the follow up and challenge study will be invited to take part.

Day 0 : Day of vaccination - P1 (All groups)

- An interim history will be taken and if considered necessary a basic physical examination emphasizing any acute complaints will be performed.
- A β-HCG urine test will be performed for women. Vaccination will not begin until the test is completed and reported as negative
- Blood will be collected for "exploratory immunology" tests, haematology and biochemistry.
- The vaccine candidate PEV3A will be administered intramuscularly as described in Section 5.3.
- *Group 2 only:* The vaccine candidate FP9 ME-TRAP will be administered intradermally as described in Section 5.3.
- Vital signs will be checked at 30 minutes (±10min) post injection. The injection site will be inspected and the largest diameter of any hardness or redness as well as the degree of pain will be documented into the CRF. An assessment for possible systemic AE's will be carried out.
- A diary card for the first week after vaccination and thermometer will be handed out to each volunteer. Full training on how to complete it and when to take the temperature will be given.

Day 2 (± 1 day): Attendance P1A (All groups)

- An interim history will be taken and, if considered necessary a basic physical examination emphasizing any acute complaints will be performed.
- Vital signs will be checked and documented
- The injection site will be inspected and the largest diameter of any hardness or redness present, as well as the degree of pain reported for the time of the visit will be documented into the CRF. Solicited adverse events (local and general) that may have occurred since the volunteer was seen on the day of vaccination will be documented in the CRF.
- Entries in the diary card will be reviewed

Day 7 (± 3 days): Attendance P1B (Groups 1 & 2) and F1 (Group 3)

- An interim history will be taken and, if considered necessary a basic physical examination emphasizing any acute complaints will be performed.
- Vital signs will be checked and documented
- The injection site will be inspected and the largest diameter of any hardness or redness present, as well as the degree of pain reported for the time of the visit will be documented into the CRF. Solicited adverse events (local and general) that may have occurred since the volunteer was seen on day 2 post vaccination will be documented in the CRF
- Blood will be collected for haematology, biochemistry, and exploratory immunology.
- Entries in the diary card will be reviewed and the card will be collected.
- *Group 3 only:* All women will undergo a β-HCG urine test.

- *Group 3 only:* Volunteers will receive their second vaccination, FP9 ME-TRAP, as described in Section 5.3.
- *Group 3 only:* Vital signs will be checked at 30 minutes (±10min) post injection. The injection site will be inspected and the largest diameter of any hardness or redness as well as the degree of pain will be documented into the CRF. An assessment for possible systemic AE's will be carried out.
- *Group 3 only:* A diary card for the week after vaccination will be handed out to each volunteer in group 3. Full training on how to complete it and when to take the temperature will be given.

Day 9 (± 1 day): Attendance F1A (Group 3 only)

- An interim history will be taken and, if considered necessary a basic physical examination emphasizing any acute complaints will be performed.
- Vital signs will be checked and documented
- The injection site will be inspected and the largest diameter of any hardness or redness present, as well as the degree of pain reported for the time of the visit will be documented into the CRF. Solicited adverse events (local and general) that may have occurred since the volunteer was seen on the day of vaccination will be documented in the CRF.
- Entries in the diary card will be reviewed

Day 14 (± 3 days): Attendance F1B (Group 3 only)

- An interim history will be taken and, if considered necessary a basic physical examination emphasizing any acute complaints will be performed.
- Vital signs will be checked and documented
- The injection site will be inspected and the largest diameter of any hardness or redness present, as well as the degree of pain reported for the time of the visit will be documented into the CRF. Solicited adverse events (local and general) that may have occurred since the volunteer was seen on day 2 post vaccination will be documented in the CRF.
- Blood will be taken for haematology, biochemistry and exploratory immunology
- Entries in the diary card will be reviewed and the card will be collected.

Day 28 (± 7 days): Attendance P2 (All groups)

- An interim history will be taken and if considered necessary a basic physical examination emphasizing any acute complaints will be performed.
- A β-HCG urine test will be performed for women. Vaccination will not begin until the test is completed and reported as negative
- Blood will be collected for "exploratory immunology" tests, haematology and biochemistry.
- The vaccine candidate PEV3A will be administered intramuscularly as described in Section 5.3.
- Vital signs will be checked at 30 minutes (±10min) post injection. The injection site will be inspected and the largest diameter of any hardness or redness as well as the degree of pain will be documented into the CRF. An assessment for possible systemic AE's will be carried out.
- A diary card for the first week after vaccination and thermometer will be handed out to each volunteer. Full training on how to complete it and when to take the temperature will be given.

This schedule will be repeated, so that visits on day 30 and day 58 will mirror that for day 2, the visits on day 35 and day 63 will be the same as for day 7, visits on day 37 and day 65 will be the same as day 9, visits on day 42 and 70 will be the same as for day 14, and visits on day 56 will be the same as day 28.

Day 77 (±7 days): Day of Challenge

Providing that sporozoite culture is successful, day 77 will also be the day of challenge. If this is so, blood samples may be taken up to 48 hours before the challenge.

- An interim history will be taken and if considered necessary a basic physical examination emphasizing any acute complaints will be performed.
- A β-HCG urine test will be performed for women volunteers
- Blood will be collected for "exploratory immunology" tests, haematology and biochemistry, analysis of gene expression and a baseline PCR sample.

If there are difficulties with sporozoite culture, subjects will be seen again before the challenge, and further blood samples may be taken again for exploratory immunology and PCR baseline.

For the challenge, subjects will travel to London with the investigators. The volunteers will be divided into two groups for challenge at Imperial College on separate days, which will be a maximum of four days apart. The allocation of volunteers to each of the challenge days will take into account each volunteer's preference but will maintain a balance in each group between groups all vaccination groups.

A Medic-Alert type card will be issued to each volunteer with information including Riamet®-sensitivity of malaria, study physician contact details and asking for volunteer to be brought to John Warin ward if they are found unwell. Each subject will also be issued with an accurate oral thermometer. If the subject does not have their own mobile telephone they will be issued with one for the duration of the study, and counselled about the importance of keeping it switched on or checking for messages regularly. In addition full contact details for each subject will be documented, including home address, home and work land-line telephone numbers where available, and next-of-kin address and telephone numbers. Subjects must also provide the investigators with the name and 24 hour telephone number of a close friend, relative or housemate who lives nearby and will be kept informed of their whereabouts for the duration of the study.

Early symptoms of malaria include a flu-like illness, fever, chills, headache, muscle pains diarrhoea and vomiting. Subjects will be encouraged to contact a trial physician on the 24 hour emergency pager if they develop such symptoms between the regular reviews, and the investigator will consider an extra clinical review and thick film if the symptoms are moderate or severe. The pager will operate from the day of challenge until their participation in the study is completed. If volunteers are unwell as a result of malaria infection and unable to attend the CCVTM for a visit, they will be visited at home by one of the Investigators. In previous challenge studies most, but not all, of the volunteers did experience some of the above symptoms. Symptoms can persist after treatment has commenced but usually last between 1 and 3 days. This is consistent with other challenge studies in the USA [57].

Day 83 - Day 91 (= Day 6 – Day 14 post challenge)

From the evening of Day 83 until Day 91, all volunteers will attend for twice daily review. All volunteers will be seen by one of the investigators at every visit. At each visit vital signs will be checked and the subject will be questioned for any symptoms of malaria, any other symptoms and any medication taken (*e.g.* paracetamol, ibuprofen)

Three mL of blood for thick film, PCR and cytokine analysis will be collected at each visit (approximately 12 hourly). The blood samples will be examined by thick-film blood smear examination immediately (see Appendix C.9) and analysed by PCR.

Day 92 – Day 100 (= Day 15 – Day 23 post challenge)

From Day 92 to Day 100 the volunteers attend for clinical review and blood sampling once daily. As the PCR technique is still under development, it cannot be used as a primary endpoint in this study, but the PCR data will be used by the Principal Investigator to provide additional safety in the assessment of volunteers post-challenge. Once a volunteer becomes slide-positive for malaria parasites treatment will be commenced as described in Section 7.

Day 112 (±7 days) (= Day 35 post challenge)

- An interim history will be taken and, if considered necessary a basic physical examination emphasizing any acute complaints will be performed.
- Vital signs will be checked and documented
- Blood will be taken for haematology, biochemistry and exploratory immunology
- After the late challenge, this will be the final visit.

Day 174 (± 14 days)

- An interim history will be taken and, if considered necessary a basic physical examination emphasizing any acute complaints will be performed.
- Vital signs will be checked and documented
- Blood will be taken for haematology, biochemistry, exploratory immunology, antibody measurements, and gene expression studies.
- All volunteers eligible for participation in the late challenge will be given information sheets regarding this, and asked to consider taking part. If they are interested they will be asked to contact the investigators to arrange a 're-screening visit'
- For all unprotected volunteers, and those not keen in taking part in the late challenge, this will be the final visit.

Late Challenge

All volunteers who are protected from malaria in the first challenge will be invited to participate in a second, late challenge. This will take place approximately 6 months (4-12 months) after the first challenge. Volunteers willing to participate will be given the information sheet describing this part of the trial and asked to attend a 're-screening visit', where eligibility will be reassessed and safety bloods taken. Volunteers will not be excluded if they have travelled to a malaria endemic area since the first challenge, as long it is not within 3 months of the late challenge, and any chemoprophylaxis they may have taken during that time satisfies the relevant exclusion criterion. These and any other issues relating to their participation in the re-challenge will be discussed with them at this visit, along with a detailed description of the study. They will also be asked to sign another consent form to indicate their willingness to take part. This challenge will be conducted according to the same procedure as the first (described in sections 6 & 7 of this protocol), and the visits will follow the same SOPs outlined in Appendix C. The termination visit for those taking part in this stage of the trial will be 35 days after the day of challenge, not 90 days as for the early challenge.

This extra challenge will therefore comprise 1 re-screening visit, and 28 challenge visits. A total volume of 399mL of blood will be taken during the late challenge.

C.2 Investigator Archiving

The ICH Guidelines for GCP state that the essential documents should be retained for at least 2 years after the last approval of a marketing application in an ICH region and until there are no pending or contemplated marketing applications in an ICH region or at least 2 years have elapsed since the formal discontinuation of clinical development of the investigational product.

C.3 Communication with Ethics Committee and National Agency

Before initiating a trial, the investigator will have written and dated approval/favourable opinion from the IRB/IEC for the trial protocol, written informed consent form, consent form updates, subject recruitment procedures (*e.g.*, advertisements), and any other written information to be provided to subjects.

As part of the investigator's written application to the IRB/IEC, the investigator will provide the IRB/IEC with a current copy of the Investigator's Brochure. If the Investigator's Brochure is updated during the trial, the investigator will supply a copy of the updated Investigator's Brochure to the IRB/IEC.

During the trial the investigator/institution should provide to the IRB/IEC all documents subject to review.

C.4 Screening and recruitment of study subjects

Subjects will have telephone screening before they are scheduled for the screening visit, to check inclusion and exclusion criteria. The screening visit may take place up to 60 days prior to vaccination. At the screening visit, before screening is undertaken, the screening process will be reviewed in detail with each prospective volunteer and all questions about the screening process answered. Each potential subject will be fully informed so that they understand the following general principles:

- Participation in the study is entirely voluntary
- Refusal to participate involves no penalty or loss of medical benefits
- The subject may withdraw from the study at any time
- The subject is free to ask questions at any time to allow him or her to understand the purpose of the study and the procedures involved
- The study involves research of an investigational vaccine
- There is no direct benefit for participating
- The volunteer's GP will be contacted to corroborate their medical history

The aims of the study and all tests to be carried out will be explained by the investigator or the experienced Clinical Research Nurse. Potential subjects will be informed that there may be leftover samples of their blood (after all testing for this study is completed), and that such samples may be stored up to a maximum of 15 years for possible future research (exploratory immunology assays). Subjects will be able to decide if they will permit such future use of any leftover samples. If a subject

elects not to permit this, all of that subject's leftover samples will be discarded after the required period of storage to meet Good Clinical Practice (GCP) and regulatory requirements.

If the volunteer agrees to undergo screening, written informed consent will be obtained. A baseline medical history and physical examination will be performed. Inclusion and exclusion criteria will be checked using a tabulated format. Vital signs will be checked. Subjects will be counselled by one of the investigators for HIV, Hepatitis B and Hepatitis C testing. Subjects will have the opportunity to ask questions about the implications of HIV testing and will be invited to read and sign the consent form for HIV testing. 20mL of blood will be drawn for full blood count (including platelet count), electrolytes, urea, creatinine, liver function tests, HIV, Hepatitis B and Hepatitis C testing, typing for HLA variants, and other genetic variants that may be relevant to the immune response to vaccines. Urinalysis, to exclude glycosuria and significant proteinuria, and a urinary pregnancy test (women only) will be performed.

The subject's general practitioner will be contacted with the written permission of the subject after satisfactory screening as notification that the subject is taking part in the study and to ascertain any significant medical history.

C.5 Obtaining informed consent

The information sheet will be made available to the volunteer at least 24 hours prior to the screening visit. At the screening visit the investigator or clinical research nurse will fully inform the subject of all aspects of the trial, going over the written information sheet and consent form with them. The subject will be given the opportunity to ask about details of the trial, and will then have time to consider whether or not to participate. If they do decide to participate, they will sign and personally date two copies of the consent form, one for them to take away and keep, and one for the Investigator. These forms will also be signed and dated by the investigator or clinical research nurse.

C.6 Suspected drug users

It is the opinion of the investigators and the sponsor that suspected intravenous drug abusers should not be included in the study. Subjects will therefore be excluded if there is any history of drug abuse, or if any of the following clinical signs of intravenous drug abuse are present at screening: multiple puncture marks over veins (in the absence of any reason for repeated venesection), the presence of track marks over veins, unexplained abscesses at injection sites, or anything that in the opinion of the investigator indicates suspected drug abuse.

C.7 Data handling

Each participant will be allocated a unique study number for the duration of the trial. This will take the form M1030### where M=Malaria, 1=Oxford, 030=local trial identifier and ### is a three digit participant code, allocated sequentially from 001.

There will be two separate databases, one which will be confidential to the investigators and clinical research nurse, will contain all the demographic details – name, date of birth, contact information etc. This database will link the volunteers' details to their unique study number, and access will be restricted to the immediate clinical team only. The second database will be more widely accessible, although only the investigators or clinical research nurse will be able to make entries or changes. In this database, volunteers will be identifiable only by their unique study number.

The Investigator has generated case report forms (CRFs) which will contain source data. These are in electronic format, forming part of a StudyBuilder™ database. This database is compliant with FDA 21 CRF part 11 regulations. It is also recommended by the WHO for use in clinical trials. It has been approved for use in clinical trials by the Oxfordshire Research Ethics Committee.

Data may only be entered into these electronic forms by one of the investigators or clinical research nurse. Access will be restricted by use of usernames and passwords. If a wrong entry is made into the forms, then these can be changed immediately or at a later date. The database allows for this, and stores the original entry, any subsequent changes and the date and time of those changes. The identity of the person making the changes will be recorded as their username.

At each visit, the form relating to that visit will be completed, collecting all protocol required information. This will include details of physical examination, vital signs, the presence or absence of any solicited or unsolicited symptoms, details of each vaccine given (type, dose, route, vial number, lot), concomitant medications and all adverse events. All information will be directly entered onto the database. Any corrections will be entered as described above.

In the event of a server shutdown or power failure, or of the need for the investigator to visit the subject away from the CCVTM, the required forms will be downloaded onto a personal computer and data entered as usual. This data will then be uploaded onto the server, as soon as the investigator returns to the office, or access to the server is returned. No data will be stored on personal computer after the database has been updated.

Source documents such as signed consent forms, copies of blood results, diary cards etc, will be stored in a file in a locked filing cabinet.

The database is stored on a secure server at the Wellcome Trust Centre. It is backed up daily. Access to the database will be via a secure website. The investigators and clinical research nurses will have full access to both databases, and will be able to enter and correct data on both. Our monitor and sponsors will have read only access. This will also be provided to the MHRA, ethics committees, or any other regulatory body for the purposes of audit.

C.8 Preparation and Feeding of Infected Mosquitoes at Imperial College

Authors: Geoff Butcher & Robert Sinden, Imperial College.

The *P. falciparum* used to challenge the volunteers is the 3D7 clone of strain NF54, isolated by Prof. D. Walliker.
Work carried out at the London School of Hygiene and Tropical Medicine has shown that 3D7 is reasonably sensitive in vitro to both lumefantrine and artemether and a marked synergism between lumefantrine and artemether has been observed (Prof. Warhurst, personal communication). The parasites are maintained in a continuous culture system in a medium containing 10% v/v human AB serum (from the Blood Transfusion Service, Colindale) heat inactivated at 56 °C for 30 min. The red cells used for culture come from four donors, all of whom have been screened (at Oxford) as for blood donation. *Anopheles stephensi* (originally obtained from Nijmegen -strain SF500) are infected with the cultured material 17 days from the beginning of gametocyte culture when sufficient mature gametocytes are present, as indicated by the ability of microgametocytes to exflagellate. The mosquitoes are fed *via* a membrane feeder containing parasite material plus fresh red cells from one of the donors and the AB human serum.

Twenty-four hours after feeding, the unfed mosquitoes are removed and the remainder maintained on a fructose/paba solution. Seven to 9 days after the infective feed, a sample of the mosquitoes are checked for the presence of oocysts. Several days before challenge of the volunteers samples are checked again for the presence of sporozoites in the salivary glands. They are then transferred to small pots containing 1 - 5 mosquitoes for feeding on the volunteers.
Infected mosquitoes are held in a secure room within the insectary and each pot of mosquitoes is kept in a strong transparent plastic storage box. All manipulations of mosquitoes are done in a secure Perspex cabinet. The small pots of mosquitoes brought out for challenge of the volunteers are held in a second pot with a second layer of netting to minimise any possibility of escape.

C.9 Thick Film Preparation

Microscope slides labelled with the subject's uniqe identification number, the date and the time will be provided with each blood sample for thick film examination.

- Put a few drops of blood onto each end of the labelled glass microscope slide and make a thick smear using an additional glass slide. Allow the film to dry as quickly as possible.
- Make up fresh Giemsa stain each day by adding four parts of phosphate buffer (Sigma) to one part of Giemsa stain (BDH).
- Pour Giemsa over the thick film.
- Allow to stain for 5 - 10 minutes.
- Wash by dipping the film into a beaker of distilled water. Air dry.
- Once the film is completely dry, examine under oil immersion (Microil, BDH). 200 fields at high power must be read before a film is declared negative.

C.10 Work involving genetically modified organisms

Information before carrying out activities involving the Contained Use of Genetically Modified Organisms* (GMOs)

SOP Ref No CCVTM 02
Version No. **1** Date of Issue **01/08/03**
Author(s) Lucy Dorrell
Author(s) role(s) Clinician Scientist

Aim: All staff using GMOs for the first time within the Trust must be informed of relevant local procedures and local rules.

<u>Procedure</u>

> 'Activity involving contained use of GMOs includes any activity in which organisms are genetically modified or in which GMOs are cultured, stored, transported, destroyed, disposed of or used in any other way'.

1. All staff who will be carrying out activities involving GMOs must:

Be given a copy of this SOP, shown where SOPs are filed locally and be trained in local procedures for issuing

> No person shall undertake any activity involving genetic modification of micro-organisms unless, before commencing that activity, he has ensured that a suitable and sufficient assessment of the risks created to human health and the environment has been carried out (HSE: Guide to GMO (contained use) regulations 2000).
> It is the management's ultimate responsibility to ensure that the working environment is safe. However, employees do have a duty to take responsible care for both their own health and safety and that of others who may be affected by their acts or omissions at work (HSE: ACGM compendium of guidance 2000 part 1, 20 & part 2, 5)

SOPs.

Be trained appropriately including understanding current legislation, what GMOs are, the main duties under the legal regulations, classification of activities involving GMOs and sources of other information and guidance especially ACGM guidance. The Health and Safety Executive produce a booklet on Contained Use of Genetically Modified Organisms. Single copies are available free from HSE books (PO Box 1999, Sudbury, Suffolk, CO10 2WA Tel 01787 881155) or it can be downloaded from their web site. www.hsebooks.co.uk. The ACGM compendium of Guidance and newsletters are available on http://www.hse.gov.uk/a-z/ The Genetically Modified Organisms (Contained Use) Regulations 2000 can be obtained for The Stationary Office http://www.tso.co.uk/ and a guide to these regulations can be obtained from HSE books.

Ensure that the Local Biological Safety Officer (*Dr Helen McShane)* is aware of the work to be carried out.

2. Where it is proposed to carry out any activity involving GMOs*, in an area for which the Trust is responsible, where no such activity has been carried out previously, the Trust Biological Safety Officer (BSO) must be contacted as soon as possible. The Trust BSO will need to inform the HSE that new premises are to be used before that work starts.

3. Before starting any new activity the Trust form 'Risk assessment under GMO (Contained Use) Regulations 2000' must be completed and sent to the BSO who will arrange assessment by the Trust Genetic Manipulation Safety Committee (GMSC). In most cases the work will have already been reviewed by an external GMSC such as at Oxford University. This should take place before the Trust assessment. It does not remove the need for approval by the Trust GMSC. The Trust committee meets quarterly. Submission deadlines and committee dates are available from the Trust BSO.

4. Risk-assessments should be reviewed regularly taking into account new information from scientific literature. Information derived from the work itself as it progresses, which has a bearing on risk, needs to be fed back into the risk assessment.

5. Records of risk assessments should be kept for 10 years from the date the work covered by those assessments finished *(HSE Guidance 2000 Part 1'57).*

6. Health surveillance for workers using GMOs is no longer absolute requirement under the contained use regulations although other more general legislation may require it *e.g.* COSHH (Committee on Substances Hazardous to Health) and / or MHSWR (Management of Health and Safety at Work Regulations 1999). The need for health surveillance would be considered by the GMSC in its assessment of any project. However they could be additional personal reasons for

certain staff to seek advice on health risks. If this is the case Occupational Health should be contacted for advice. Considerations could include:

Evidence of defective barriers to infection (disorders of skin, respiratory tract or alimentary canal)
Immune competence
Treatment with antibiotics involved in the experimental programme, therapeutic use of steroids and some self-medication which could influence resistance to infection.
Relevant medical history (*e.g.* recurrent infections, asthma,)
Pregnancy or breast feeding

C.11 Vaccine Handling

	SOP Title	Vaccine Handling	
SOP No	CCVTM 03		
Author	Lucy Dorrell	Principal Investigator	
Version no.	1	Issue date	01/08/03

INFORMATION FOR PHARMACY STAFF

The Principal Investigator must ensure that the pharmacy staff have been briefed on the protocol and understand the study requirements relating to storage of study materials, dispensing of study product and maintenance of study records.

STUDY DOCUMENTATION

The following documentation must be provided to the pharmacist prior to delivery of the study products:

Study protocol
Certificate of Analysis of study products
Dispensing records (if applicable)
Information regarding expiry date and storage conditions of study products
List of study contact details

VACCINE HANDLING

Requirement for following Contained Use Regulations

Because the MVA / Fowlpox vaccine is a genetically modified organism its handling and use are regulated by legislation. The Department of the Environment has confirmed that the present clinical trial may be undertaken under so-called "Contained Use" regulations (as distinct from "Deliberate Release" regulations) provided that certain procedures are followed, as specified below.

Rationale for Contained Use Categorisation

The virus to be used has been classified as a group 1 micro-organism. It is believed to be incapable of replication in humans and thus there should be no viral shedding from the vaccinated individuals. The vaccine and dressings in contact with the immunisation site will be handled so as to avoid any environmental contamination by the MVA / Fowlpox vaccine.

Potential Routes of Environmental Contamination

There is a risk of environmental contamination through breakage of a vaccine vial during handling or transport; through accidental spillage during immunisation procedures; through leakage from the vaccination site after immunisation; through incorrect disposal of dressings used to cover the immunisation site.

Overall Assessment of Risk to Environment

By observing the measures listed in this procedure the risk of any release of MVA / Fowlpox to the environment will be extremely low. Even if this were to occur the probability of any replication of the virus occurring in the absence of special tissue culture conditions is very small. Therefore the overall probability of any adverse environmental effect is assessed as negligible.

Delivery and storage of the vaccine(s)

The vaccine is to be delivered and stored in a labelled sealable container, either in a designated -80°C freezer in the Oxford Haemophilia Centre (OHC), Churchill Hospital, Oxford or in a designated -20°C freezer in the Pharmacy Dept., Churchill Hospital. Both freezers must have an incorporated alarm system and a system for recording the temperature so that this information can be made available to the PI.

The freezer in which the material is stored must be locked at all times. Only designated study site personnel will have access to the freezer. In addition, the Laboratory Manager of OHC must have access to the material in case there is a power or generator failure and the vaccine needs to be moved to another freezer.

Transport of vaccines within the Churchill site

The vaccine vial must be collected from the OHC / Pharmacy freezer by the trial physician / nurse and carried in a rigid, impermeable, sealable container to the CCVTM outpatient clinic. The clinic will be reached by going outside the building and re-entering through the main entrance, avoiding the office area.

Thawing and administration of the vaccine(s)

The vaccine will be prepared for administration in the CCVTM outpatient clinic, either in the treatment room, or in one of the consultation rooms. The trial physician / nurse must ensure that the room to be used for vaccination is equipped with a spill kit (see SOP 05). The subject to be vaccinated must be seated on a plastic chair. In the case of any spillage the contaminated surface must be cleaned according to local disinfection policy (see SOP 05 spillage).

For use as an intradermal injection at each vaccination time point the vial which has been removed from the freezer will be allowed to thaw at room temperature, avoiding shaking of the vial. The vaccine will be used as supplied by the manufacturer with no further preparation.

Disposal of vaccines and all materials after immunisation

Reconstituted vaccine must be used within the recommended period, according to the manufacturer's instructions. Once opened, vials must not be re-used.

If vials are to be disposed of they must be placed within a sharps box, which will be autoclaved the same day (see SOP 07). If used vials are to be stored these must be collected into a sealable plastic bag / container and taken in a secondary container to the –80°C freezer in the CCVTM laboratories for storage until the end of the study, following the route described in the section entitled Transport of vaccines within the Churchill site.

All materials that have been in contact with the vaccine or vaccination site (*e.g.* dressings) must be punctiliously disposed of into an autoclave bag or suitable container for autoclaving. All sharps must be disposed of immediately after use into a sharps bin for autoclaving. Waste bags and sharps boxes must be placed in a robust container suitable for transport to the autoclave. The trial physician will be responsible for ensuring that this waste is autoclaved in the CCVTM laboratories or OHC.
A record of autoclaved waste will be kept in the CCVTM laboratories, by filing autoclave printouts in a designated log book with the date, time, name and signature of the person responsible for autoclaving the waste (see SOP 07).

C.12 Procedures in the event of accidental exposure to GMOs

SOP Ref No CCVTM 04
Version No. **1** Date of Issue **01/08/03**
Author(s) Lucy Dorrell
Author(s) role(s) Clinician Scientist

Aim: To minimise the consequences of accidental exposure to GMOs.

<u>Procedures</u>

Needle-stick

1. Encourage bleeding of wound.
2. Wash puncture site thoroughly with soap and water.
3. Dry area using disposable material.
4. Cover with waterproof dressing.
5. Contact senior staff, the local Biological Safety Officer and Occupational Health for advice.
6. As a minimum careful observation within reach of medical attention should be maintained for at least three hours
7. All materials exposed to GMO including materials used to clean and dry the site must be disposed of as GM waste, see SOP CCVTM 07.
8. The Local Biological Safety officer or other appropriate staff member must refer to SOP CCVTM 06 'Reporting of Incidents involving Category I GMOs' and follow through the reporting procedure.

Skin splash

1. Wash area thoroughly with soap and water.
2. Dry area using disposable material.
3. Cover with waterproof dressing.
4. Contact senior staff, the local Biological Safety Officer and occupational health for advice.
5. If broken or damaged skin was exposed, or if there is any evidence of skin being or becoming irritated follow items 6-8 above as for needle-stick.
6. If the person exposed was a staff or patient not involved in the work follow items 6-8 above as for needle-stick.

Eye splash

1. Irrigate eye immediately with sterile eye wash solution if available or water.
2. Follow items 5-8 above as for needle-stick.

Mouth splash

1. Wash out mouth immediately with water several times.
2. Follow items 5-8 above as for needle-stick.

Inhalation

1. Follow items 5-8 above as for needle-stick.

C.13 Decontamination

SOP Title **Decontamination of small spills of liquid containing Category I Genetically Modified Organisms**

SOP Ref No CCVTM 05

Version No. 1 Date of Issue **01/08/03**

Author(s) Lucy Dorrell

Author(s) role(s) Clinician Scientist

Aim: To clear up liquid spills of GMOs with minimal exposure of operator in order to maintain containment and minimise risk to people and the environment.

Procedure

A spill kit must be available at all times when GMOs are being used. This should contain Virkon Powder sachets, disposable gloves, disposable apron and eye protection. If GMOs which are potentially infectious by inhalation or ocular exposure *(e.g. adenovirus vectors)* are in use on the premises eye goggles and disposable masks must be included in the spill kit.

1. Wearing appropriate protective clothing pour sufficient Virkon powder on to the spill to absorb all the liquid.
2. Leave for 5 minutes.
3. Clear up with absorbent material.
4. Thoroughly wash the affected area first with 2% Virkon, followed by thorough washing with water. Do not use other disinfectants or alcohol at this stage as it is likely to cause frothing or smearing which may be difficult to remove. If the surface is to be left sterile it may be washed with 70% alcohol or other sterilising solution AFTER the water washes.
5. Dispose of all materials as in SOP CCVTM 03 (Vaccine handling)

Caution – Virkon is the disinfectant of choice due to its broad-spectrum activity against micro-organisms and low toxicity to humans and animals. However prolonged exposure will damage metal surfaces causing pitting which in turn makes the surface difficult to clean in the future. Virkon will decontaminate the area in a few minutes, longer exposure does not increase hygiene whilst risking damaging surfaces.

C.14 Reporting of incidents

SOP Title **Reporting of Incidents Involving Genetically Modified Organisms**

Version No. **1** **Date of Issue** **01/08/03**

Author(s) Lucy Dorrell
Author(s) role(s) Clinician Scientist

Aim: To ensure incidents involving GMOs are reported through appropriate lines to facilitate staff care, reduce the risk of recurrence and to support the needs of the Trust's clinical governance obligations.

Policies available on the ORH Intranet
http://orhweb.orh.nhs.uk/clinicalgovernance/
Risk Management Policy
Incidents, hazards and near misses (will replace Incident Reporting Policy)
Needle-stick and splash incidents
http://orhweb.orh.nhs.uk/estates/ (under non-clinical risk)
COSHH policy
Waste management policies and procedures
http://orhweb.orh.nhs.uk/infectioncontrol/
Policies relating to patients and staff

Procedure

1. All staff carrying out work involving GMOs must be aware of the biological risks of those GMOs before commencing work.

2. All accidents or incidents (including near misses) must be reported as soon as possible to each of the following in the first instance

- The Principal Investigator
- The Project Manager if different from above
- The Chair or Vice Chair of the Trust GM Safety Committee
- The Local Biological Safety Officer

3. The following must also be informed as soon as is practical, usually by either the principal investigator or the local Biological Safety Officer

- The Trust Biological Safety Officer
- Safety Officers in any other organisation involved in the work, typically the University of Oxford Safety Officer and any commercial sponsor
- The Trust Occupational Health Service if not already involved
- Clinical Risk Management via the Trust's incident reporting system.

4. Records must be kept of all persons contacted, their response, any action taken and any follow-up actions.

Typical incidents which would require reporting

- Needle stick injuries
- Eye splashes
- Spillage of whole vials – rather than just a minor splash during manipulation
- Dropped samples
- Accidental exposure of staff or patients not involved in the work

If in doubt about whether an incident is reportable the Biological Safety Officer should be contacted for advice.

C.15 Deactivation of GMO waste by autoclaving

SOP Ref No **CCVTM 07**

Version No. **1** Date of Issue **01/08/03**

Author(s) Lucy Dorrell

Author(s) role(s) Clinician Scientist

Aim: To ensure deactivation of waste generated by work involving genetically modified organisms by autoclaving prior to disposal.

Background

The Contained Use Regulations 2000 and the Environmental Protection Act 1990 require risk assessments for work involving GMOs to cover environmental risk. Under part IV of the Environmental Protection Act it is an offence to deliberately release and GMO into the environment or to allow it to escape without prior consent of the Secretary of State.

All waste generated from contained use activities must be completely deactivated using validated means prior to disposal. Once deactivated and disposed of it no longer falls under the Contained Use Regulations 2000 but it will be subject to the waste and pollution legislation that applies to any waste.

Waste containing GMOs MUST NOT enter the Trust waste system, the contractors who remove trust waste for incineration, heat treatment or any other method are not registered for handling GMOs and such routes of disposal would be an offence under the above legislation. Once autoclaved the waste may be disposed of as clinical waste or general waste as appropriate.

Procedures

1. All waste generated must be immediately placed in a suitable receptacle, *e.g.* sharps into a sharps box, swabs and dressings into a hazard waste bag.
2. Sharps boxes and waste bags must be placed into a robust container suitable both for transport to the autoclave and for the autoclaving process *e.g.* a metal box with a lid and handles which is large enough to contain the waste but small enough to fit into the autoclave.
3. Sealed boxes (such as linbins) are not suitable for autoclaving, the container must allow steam penetration for effective deactivation of the GMO waste.
4. The boxes must be transported to the autoclave and autoclaved by staff trained in working with GMOs.
5. The route taken must minimise the risk of potential exposure to personnel *i.e.* transport from the CCVTM clinical area to the CCVTM laboratories or the Oxford Haemophilia Centre should be by going outside the building and re-entering the corridor, avoiding the office area.
6. Waste containing GMOs must be deactivated on the day it is produced, in exceptional circumstances when it has to be stored overnight (*e.g.* autoclave failure) it must be stored such that persons not trained in working with GMOs are not at risk of exposure. (*e.g.* cat 3 lobby area).
7. Records must be kept of date generated, person responsible, method of disposal, date and time of disposal, evidence of effective deactivation (*e.g.* autoclave printout).

NOTE: Waste which has been fully chemically deactivated does not require further deactivation by autoclaving, *e.g.* swabs and paper towels used to mop up GMO spills using Virkon should still be treated as GM waste, items fully immersed in 2% Virkon do not need to be further deactivated prior to disposal.

C.16 Multiple use of vaccine vials containing more than a single dose

SOP Ref No **CCVTM 09**

Version No. **1** **Date of issue: 27. Oct 2003**

Author(s) **Michael Walther**

Author(s) role(s) **Clinical Research Fellow**

Aim **To regulate the GMO specific issues related to multiple use of vaccine vials**

Introduction
Due to the nature of the manufacturing process recombinant viral vaccines are filled into the vials at different concentrations. Depending on the volume per vial and the dose to be administered the amount of vaccine in a vial may be sufficient for 2 or more doses.
When the same type of vaccine is given to more than 1 individual on the same day, it is possible to draw up the syringes for all subjects from the same vaccine vial.

Procedure
1. After the vaccine has been allowed to adapt to room temperature, wipe the top of the injection port with a sterile swab containing alcohol.
2. Allow the alcohol to evaporate for at least 30 seconds.
3. Use a 0.3 mL syringe to draw up the volume needed to vaccinate the first subject via the injection port.
4. If the second dose will have to be taken out of this vial more than 30 min later, transfer the vial into a sealed polystyrene container suitable for biological specimens (*e.g.* Sarstedt EN 829), and store in the alarmed, temperature controlled fridge of the Oxford Vaccine Group at 4-8°C for up to 8 hours in the CCVTM. The fridge is placed in room 12 on the same corridor as the outpatient clinic rooms where the vaccine is administered.
5. Subsequent doses of the vaccine must be drawn up as described in point 1-3 of these instructions.
6. At the end of a vaccination day dispose of the vial of as described in SOP No CCVTM 07
7. Handle the vaccine and associated procedures are described in SOPs No CCVTM 1-8. This SOP only refers to multiple use of vaccine vials containing more than 1 dose per vial.

C.17 Protocol compliance

The investigator should conduct the trial in compliance with the protocol agreed to by the sponsor and the regulatory authority and which was given approval/favourable opinion by the IRB/IEC. The investigator/institution and the sponsor should sign the protocol, or an alternative contract, to confirm agreement.

The investigator should not implement any deviation from, or changes of the protocol without agreement by the sponsor and prior review and documented approval/favourable opinion from the IRB/IEC of an amendment, except where necessary to eliminate an immediate hazard(s) to trial subjects, or when the change(s) involves only logistical or administrative aspects of the trial (*e.g.*, change in monitor(s), change of telephone number(s)).

The investigator, or person designated by the investigator, will document and explain any deviation from the approved protocol in the protocol deviation file.

The investigator may implement a deviation from, or a change of, the protocol to eliminate an immediate hazard(s) to trial subjects without prior IRB/IEC approval/favourable opinion. As soon as possible, the

implemented deviation or change, the reasons for it, and, if appropriate, the proposed protocol amendment(s) should be submitted:

(a) to the IRB/IEC for review and approval/favourable opinion,
(b) to the sponsor for agreement and, if required,
(c) to the regulatory authority(ies).

BIBLIOGRAPHICAL REFERENCES

1. WHO, *Malaria Fact Sheet*. 1996. **94**.
2. Snow, R.W., et al., *The global distribution of clinical episodes of Plasmodium falciparum malaria.* Nature, 2005. **434**(7030): p. 214-7.
3. Webster, D., et al., *Safety of recombinant fowlpox strain 9 and modified vaccinia virus Ankara vaccines against liver-stage P. falciparum malaria non-immune volunteers.* In press (Vaccine), 2005.
4. Webster, D.P., et al., *Enhanced T cell-mediated protection against malaria in human challenges by using the recombinant poxviruses FP9 and modified vaccinia virus Ankara.* Proc Natl Acad Sci U S A, 2005. **102**(13): p. 4836-41.
5. Bejon, P., et al., *Calculation of Liver-to-Blood Inocula, Parasite Growth Rates, and Preerythrocytic Vaccine Efficacy, from Serial Quantitative Polymerase Chain Reaction Studies of Volunteers Challenged with Malaria Sporozoites.* J Infect Dis, 2005. **191**(4): p. 619-26.
6. Nussenzweig, R.S., et al., *Protective immunity produced by the injection of x-irradiated sporozoites of plasmodium berghei.* Nature, 1967. **216**(111): p. 160-2.
7. Weiss, W.R., et al., *Cytotoxic T cells recognize a peptide from the circumsporozoite protein on malaria-infected hepatocytes.* J Exp Med, 1990. **171**(3): p. 763-73.
8. Wipasa, J., et al., *Identification of T cell epitopes on the 33-kDa fragment of Plasmodium yoelii merozoite surface protein 1 and their antibody-independent protective role in immunity to blood stage malaria.* J Immunol, 2002. **169**(2): p. 944-51.
9. Aidoo, M., et al., *Identification of conserved antigenic components for a cytotoxic T lymphocyte-inducing vaccine against malaria.* Lancet, 1995. **345**(8956): p. 1003-7.
10. Hill, A.V., et al., *Molecular analysis of the association of HLA-B53 and resistance to severe malaria.* Nature, 1992. **360**(6403): p. 434-9.
11. Malik, A., et al., *Human cytotoxic T lymphocytes against the Plasmodium falciparum circumsporozoite protein.* Proc Natl Acad Sci U S A, 1991. **88**(8): p. 3300-4.
12. Plebanski, M., et al., *Precursor frequency analysis of cytotoxic T lymphocytes to pre-erythrocytic antigens of Plasmodium falciparum in West Africa.* J Immunol, 1997. **158**(6): p. 2849-55.
13. Flanagan, K.L., et al., *Broadly distributed T cell reactivity, with no immunodominant loci, to the pre-erythrocytic antigen thrombospondin-related adhesive protein of Plasmodium falciparum in West Africans.* Eur J Immunol, 1999. **29**(6): p. 1943-54.
14. Hill, A.V., et al., *Common west African HLA antigens are associated with protection from severe malaria.* Nature, 1991. **352**(6336): p. 595-600.
15. Aidoo, M., et al., *Cytotoxic T-lymphocyte epitopes for HLA-B53 and other HLA types in the malaria vaccine candidate liver-stage antigen 3.* Infect Immun, 2000. **68**(1): p. 227-32.
16. Plebanski, M., et al., *Altered peptide ligands narrow the repertoire of cellular immune responses by interfering with T-cell priming.* Nat Med, 1999. **5**(5): p. 565-71.
17. Plebanski, M., et al., *Protection from Plasmodium berghei infection by priming and boosting T cells to a single class I-restricted epitope with recombinant carriers suitable for human use.* Eur J Immunol, 1998. **28**(12): p. 4345-55.
18. Gilbert, S.C., et al., *Ty virus-like particles, DNA vaccines and Modified Vaccinia Virus Ankara; comparisons and combinations.* Biol Chem, 1999. **380**(3): p. 299-303.
19. Schneider, J., et al., *Enhanced immunogenicity for CD8+ T cell induction and complete protective efficacy of malaria DNA vaccination by boosting with modified vaccinia virus Ankara.* Nat Med, 1998. **4**(4): p. 397-402.
20. Sedegah, M., et al., *Boosting with recombinant vaccinia increases immunogenicity and protective efficacy of malaria DNA vaccine.* Proc Natl Acad Sci U S A, 1998. **95**(13): p. 7648-53.
21. Schneider, J., et al., *A prime-boost immunisation regimen using DNA followed by recombinant modified vaccinia virus Ankara induces strong cellular immune responses against the Plasmodium falciparum TRAP antigen in chimpanzees.* Vaccine, 2001. **19**(32): p. 4595-602.
22. McShane, H., et al., *Enhanced immunogenicity of CD4(+) t-cell responses and protective efficacy of a DNA-modified vaccinia virus Ankara prime-boost vaccination regimen for murine tuberculosis.* Infect Immun, 2001. **69**(2): p. 681-6.

23. McShane, H., et al., *Recombinant modified vaccinia virus Ankara expressing antigen 85A boosts BCG-primed and naturally acquired antimycobacterial immunity in humans.* Nat Med, 2004. **10**(11): p. 1240-4.
24. Hanke, T., et al., *Enhancement of MHC class I-restricted peptide-specific T cell induction by a DNA prime/MVA boost vaccination regime.* Vaccine, 1998. **16**(5): p. 439-45.
25. Sullivan, N.J., et al., *Development of a preventive vaccine for Ebola virus infection in primates.* Nature, 2000. **408**(6812): p. 605-9.
26. Amara, R.R., et al., *Control of a mucosal challenge and prevention of AIDS by a multiprotein DNA/MVA vaccine.* Science, 2001. **292**(5514): p. 69-74.
27. Reece, W.H., et al., *A CD4(+) T-cell immune response to a conserved epitope in the circumsporozoite protein correlates with protection from natural Plasmodium falciparum infection and disease.* Nat Med, 2004. **10**(4): p. 406-10.
28. Moorthy, V.S., et al., *Safety of DNA and modified vaccinia virus Ankara vaccines against liver-stage P. falciparum malaria in non-immune volunteers.* Vaccine, 2003. **21**(17-18): p. 2004-11.
29. Moorthy, V., M. Pinder, and W. Reece, *Safety and immunogenicity of DNA and modified vaccinia virus Ankara malaria vaccination in African adults*, in *J Infect Dis*. 2003.
30. McConkey, S.J., et al., *Enhanced T-cell immunogenicity of plasmid DNA vaccines boosted by recombinant modified vaccinia virus Ankara in humans.* Nat Med, 2003. **9**(6): p. 729-35.
31. Mayr, A., et al., *[The smallpox vaccination strain MVA: marker, genetic structure, experience gained with the parenteral vaccination and behavior in organisms with a debilitated defence mechanism (author's transl)].* Zentralbl Bakteriol [B], 1978. **167**(5-6): p. 375-90.
32. Mahnel, H. and A. Mayr, *[Experiences with immunization against orthopox viruses of humans and animals using vaccine strain MVA].* Berl Munch Tierarztl Wochenschr, 1994. **107**(8): p. 253-6.
33. Stickl, H., et al., *[MVA vaccination against smallpox: clinical tests with an attenuated live vaccinia virus strain (MVA) (author's transl)].* Dtsch Med Wochenschr, 1974. **99**(47): p. 2386-92.
34. Akira, S., K. Takeda, and T. Kaisho, *Toll-like receptors: critical proteins linking innate and acquired immunity.* Nat Immunol, 2001. **2**(8): p. 675-80.
35. Chakrabarti, S., K. Brechling, and B. Moss, *Vaccinia virus expression vector: coexpression of beta-galactosidase provides visual screening of recombinant virus plaques.* Mol Cell Biol, 1985. **5**(12): p. 3403-9.
36. Mayr, A. and K. Malicki, *[Attenuation of virulent fowl pox virus in tissue culture and characteristics of the attenuated virus].* Zentralbl Veterinarmed B, 1966. **13**(1): p. 1-13.
37. Somogyi, P., J. Frazier, and M.A. Skinner, *Fowlpox virus host range restriction: gene expression, DNA replication, and morphogenesis in nonpermissive mammalian cells.* Virology, 1993. **197**(1): p. 439-44.
38. Brown, M., et al., *Dendritic cells infected with recombinant fowlpox virus vectors are potent and long-acting stimulators of transgene-specific class I restricted T lymphocyte activity.* Gene Ther, 2000. **7**(19): p. 1680-9.
39. Laidlaw, S.M. and M.A. Skinner, *Comparison of the genome sequence of FP9, an attenuated, tissue culture-adapted European strain of Fowlpox virus, with those of virulent American and European viruses.* J Gen Virol, 2004. **85**(Pt 2): p. 305-22.
40. Qingzhong, Y., et al., *Protection against turkey rhinotracheitis pneumovirus (TRTV) induced by a fowlpox virus recombinant expressing the TRTV fusion glycoprotein (F).* Vaccine, 1994. **12**(6): p. 569-73.
41. Gluck, R., et al., *Immunopotentiating reconstituted influenza virus virosome vaccine delivery system for immunization against hepatitis A.* J Clin Invest, 1992. **90**(6): p. 2491-5.
42. Zurbriggen, R., *Immunostimulating reconstituted influenza virosomes.* Vaccine, 2003. **21**(9-10): p. 921-4.
43. Zurbriggen, R., et al., *IRIV-adjuvanted hepatitis A vaccine: in vivo absorption and biophysical characterization.* Prog Lipid Res, 2000. **39**(1): p. 3-18.
44. Holzer, B.R., et al., *Immunogenicity and adverse effects of inactivated virosome versus alum-adsorbed hepatitis A vaccine: a randomized controlled trial.* Vaccine, 1996. **14**(10): p. 982-6.
45. Moorthy, V.S., et al., *A Randomised, Double-Blind, Controlled Vaccine Efficacy Trial of DNA/MVA ME-TRAP Against Malaria Infection in Gambian Adults.* Plos Med, 2004. **1**(2): p. e33.
46. Moorthy, V.S., et al., *Phase 1 evaluation of 3 highly immunogenic prime-boost regimens, including a 12-month reboosting vaccination, for malaria vaccination in Gambian men.* J Infect Dis, 2004. **189**(12): p. 2213-9.
47. Mueller, M.S., et al., *Induction of parasite growth-inhibitory antibodies by a virosomal formulation of a peptidomimetic of loop I from domain III of Plasmodium falciparum apical membrane antigen 1.* Infect Immun, 2003. **71**(8): p. 4749-58.
48. Narum, D.L., et al., *Immunization with parasite-derived apical membrane antigen 1 or passive immunization with a specific monoclonal antibody protects BALB/c mice against lethal Plasmodium yoelii yoelii YM blood-stage infection.* Infect Immun, 2000. **68**(5): p. 2899-906.
49. Deans, J.A., et al., *Vaccination trials in rhesus monkeys with a minor, invariant, Plasmodium knowlesi 66 kD merozoite antigen.* Parasite Immunol, 1988. **10**(5): p. 535-52.

50. Amante, F.H., et al., *A cryptic T cell epitope on the apical membrane antigen 1 of Plasmodium chabaudi adami can prime for an anamnestic antibody response: implications for malaria vaccine design.* J Immunol, 1997. **159**(11): p. 5535-44.
51. Stoute, J.A., et al., *A preliminary evaluation of a recombinant circumsporozoite protein vaccine against Plasmodium falciparum malaria. RTS,S Malaria Vaccine Evaluation Group.* N Engl J Med, 1997. **336**(2): p. 86-91.
52. Alonso, P.L., et al., *Efficacy of the RTS,S/AS02A vaccine against Plasmodium falciparum infection and disease in young African children: randomised controlled trial.* Lancet, 2004. **364**(9443): p. 1411-20.
53. Moreno, R., et al., *Exploiting conformationally constrained peptidomimetics and an efficient human-compatible delivery system in synthetic vaccine design.* Chembiochem, 2001. **2**(11): p. 838-43.
54. Kirkwood, B.R., *Essentials of medical statistics.* 1988: p. 185.
55. van Vugt, M., et al., *Artemether-lumefantrine for the treatment of multidrug-resistant falciparum malaria.* Trans R Soc Trop Med Hyg, 2000. **94**(5): p. 545-8.
56. Edwards, I.R. and C. Biriell, *Harmonisation in pharmacovigilance.* Drug Saf, 1994. **10**(2): p. 93-102.
57. Church, L.W., et al., *Clinical manifestations of Plasmodium falciparum malaria experimentally induced by mosquito challenge.* J Infect Dis, 1997. **175**(4): p. 915-20.
