# Supplementary material for: Evidence of Blood Stage Efficacy with a Virosomal Malaria Vaccine in a Phase IIa Clinical Trial
Source: PLoS One. 2008 Jan 30;3(1):e1493. doi: 10.1371/journal.pone.0001493 (PMC2204057; doi:10.1371/journal.pone.0001493)
Supplement: Addendum S1 — Trial Protocol Addendum (0.02 MB PDF) [file pone.0001493.s005.pdf]

VAC030

ADDENDUM TO PROTOCOL

Assessment of protection against malaria by sporozoite challenge of healthy adults vaccinated with the virosomal vaccine PEV3A and FP9-MVA ME-TRAP

CTA number 21584/0210/001-0001
Eudract number 2005-001041-42

18 January 2006

This malaria vaccine study was originally designed to assess the safety, immunogenicity and efficacy of two candidate malaria vaccine regimens, PEV3A and FFM ME-TRAP. Three main study groups are outlined in the protocol, Group 1 received PEV3A alone, Group 2 received both PEV3A and FFM ME-TRAP on the same day in different arms, and Goup 3 was to receive both vaccines in different arms, but given one week apart. We started the study with groups 1 and 2, and planned to subsequently recruit and commence group 3.

The results of the challenge obtained with groups 1 and 2 were not as good as expected, showing no complete protection, although some delay to diagnosis of malaria.

We do not believe that continuing with the study as planned and recruiting and challenging volunteers in group 3 would any extra information. It would therefore be unethical to undertake this part of the study, and expose healthy volunteers to a potentially dangerous disease for no benefit.

Group 3 as described in the protocol has been dropped, and this part of the study has been abandoned.
