# Supplementary material for: Evidence of Blood Stage Efficacy with a Virosomal Malaria Vaccine in a Phase IIa Clinical Trial
Source: PLoS One. 2008 Jan 30;3(1):e1493. doi: 10.1371/journal.pone.0001493 (PMC2204057; doi:10.1371/journal.pone.0001493)
Supplement: Ethics Approval Letter S1 — (0.45 MB PDF) [file pone.0001493.s002.pdf]

VAC 030	2	1	9

NHS

Oxfordshire REC A
Room 13, Manor House
John Radcliffe Hospital
Headley Way, Headington
Oxford
OX3 9DU

Telephone: 01865 222758
Facsimile: 01865 222699
Email: gordon.riddell@orh.nhs.uk

10 June 2005

Prof Adrian V. S. Hill
Professor of Human Genetics
University of Oxford
Wellcome Trust Centre for Human Genetics
Roosevelt Drive
Oxford
OX3 7BN

Dear Prof Hill

**Full title of study:**	**Assessment of protection against malaria by sporozoite challenge of healthy adults vaccinated with the virosomal vaccine PEV3A and FP9-MVA ME-TRAP**
**REC reference number:**	**05/Q1604/69**
**Protocol number:**	**1.1**
**EudraCT number:**	**2005-001041-42**

Thank you for your letter of 09 May 2005, responding to the Committee's request for further information on the above research and submitting revised documentation.

The further information has been considered on behalf of the Committee by the Chair.

Confirmation of ethical opinion

On behalf of the Committee, I am pleased to confirm a favourable ethical opinion for the above research on the basis described in the application form, protocol, consent form and supporting documentation as revised.

Conditions of approval

The favourable opinion is given provided that you comply with the conditions set out in the attached document. You are advised to study the conditions carefully.

Approved documents

The final list of documents reviewed and approved by the Committee is as follows:

Document	*Version*	*Date*
Application	4.1	16 March 2005
Investigator CV	Professor Adrian Hill	(None Specified)
Protocol	1.1	16 March 2005
Covering Letter		16 March 2005
Summary/Synopsis	2 (Groups 1 - 3)	10 May 2005
Peer Review	Referees 1 to 4	(None Specified)

Compensation Arrangements	Indemnity and clinical trial insurance	16 March 2005
Sample Diary/Patient Card		(None Specified)
Copies of Advertisements	Poster	(None Specified)
Copies of Advertisements	1 (Text for radio advertisement)	16 March 2005
Copies of Advertisements	http://www.malaria-vaccines.org.uk/10.shmtl	16 March 2005
GP/Consultant Information Sheets	2	09 May 2005
GP/Consultant Information Sheets	2 (Late Challenge)	09 May 2005
GP/Consultant Information Sheets	1.0 (March 05)	(None Specified)
GP/Consultant Information Sheets	2 (GP response)	09 May 2005
Participant Information Sheet	2 (Late Challenge)	09 May 2005
Participant Information Sheet	2 (Controls)	09 May 2005
Participant Information Sheet	2 (Groups 1 - 3)	09 May 2005
Participant Consent Form	2 (Controls)	09 May 2005
Participant Consent Form	1 (Groups 1 - 3)	15 March 2005
Participant Consent Form	2 (Late Challenge)	09 May 2005
Investigator's Brochure	Malaria Vaccine IB	20 August 2003
Investigator's Brochure	Clinical IB	16 March 2005
Response to Request for Further Information		09 May 2005
Book Chapter		(None Specified)

Management approval

The study should not commence at any NHS site until the local Principal Investigator has obtained final management approval from the R&D Department for the relevant NHS care organisation.

Membership of the Committee

The members of the Ethics Committee who were present at the meeting are listed on the attached sheet.

Notification of other bodies

The Committee Administrator will notify the research sponsor and the Medicines and Healthcare products Regulatory Agency that the study has a favourable ethical opinion.

Statement of compliance

This Committee is recognised by the United Kingdom Ethics Committee Authority under the Medicines for Human Use (Clinical Trials) Regulations 2004, and is authorised to carry out the ethical review of clinical trials of investigational medicinal products.

The Committee is fully compliant with the Regulations as they relate to ethics committees and the conditions and principles of good clinical practice.

The Committee is constituted in accordance with the Governance Arrangements for Research Ethics Committees (July 2001) and complies fully with the Standard Operating Procedures for Research Ethics Committees in the UK.

With the Committee's best wishes for the success of this project,

Yours sincerely

J Franklin

pp **Mr B Shine**
Chair

Enclosures:

Standard approval conditions
Site approval form (SF1)
